# The EH-binding protein EHBP1 operates in a ciliary functional module affected by INPP5E dysfunction

**DOI:** 10.1242/jcs.264091

**Published:** 2026-07-21

**Authors:** Kae R. Whiting, Mariam Aslanyan, Imke Peters, Stef Letteboer, Ideke J. C. Lamers, Mohamed-Ali Jarboui, Tina Beyer, Katrin Dahlke, Karsten Boldt, Ronald Roepman

**Affiliations:** ^1^ Department of Human Genetics, Research Institute for Medical Innovation, Radboud University Medical Center, 6525 GA, Nijmegen, Netherlands; ^2^ Eberhard Karls University of Tübingen, Core Facility for Medical Proteomics, 72074 Tübingen, Germany; ^3^ Eberhard Karls University of Tübingen, Institute for Ophthalmic Research, 72074 Tübingen, Germany

**Keywords:** INPP5E, EHBP1, Proximity proteomics, BioID, Primary cilium

## Abstract

Inositol polyphosphate-5-phosphatase E (*INPP5E*) encodes the ciliary protein INPP5E, which plays an important role in regulating the phospholipid membrane makeup of the primary cilium. Here, we utilize proximity labeled proteomics of INPP5E to broaden the number of known functional modules that work in close proximity to the protein. In doing so, we identified the EH-binding protein EHBP1 as a ciliary protein that localizes to the basal body and ciliary compartment of the primary cilium in human-derived fibroblasts and RPE cells. Additionally, we show that EHBP1 localizes to the rudimentary outer segment membrane of developing photoreceptors in retinal organoids. Dysfunction of INPP5E – either due to pathogenic variants in human fibroblasts or CRISPR/Cas9-generated loss-of-function variants in human retinal organoids – causes the localization of EHBP1 to be altered. Our data suggest that EHBP1 functions at the primary cilium and photoreceptors, where it is regulated by INPP5E. This provides further insights into the disease pathogenesis of retinal ciliopathies caused by pathogenic variants in *INPP5E*, and suggests that *EHBP1* might be a candidate gene for retinal ciliopathies.

## Teaser

**Summary:** EHBP1, an actin dynamics and endocytosis-linked protein, localizes to the primary cilium, where it operates in a ciliary protein module affected by loss of INPP5E function.

## INTRODUCTION

Inositol polyphosphate-5-phosphatase E (*INPP5E*) encodes the protein INPP5E, a phosphatase important for regulating the phosphatidylinositol makeup of the plasma membrane. INPP5E localizes to the primary cilium (Bielas et al., 2009; Jacoby et al., 2009), a microtubule-based sensory organelle present on most quiescent cells that plays an essential role in regulating signaling pathways during organism development and tissue homeostasis (Mill et al., 2023). INPP5E hydrolyzes the 5′ phosphate group from phosphatidylinositol 4,5-bisphosphate [PI(4,5)P_2_] to form phosphatidylinositol 4-phosphate [PI(4)P] in the cilium, thus giving the ciliary membrane a unique identity compared to that of the plasma membrane (Chávez et al., 2015; Garcia-Gonzalo et al., 2015; Phua et al., 2017). By regulating the membrane identity, INPP5E affects downstream regulation of the cell response to different stimuli including the regulation of signaling pathways (Constable et al., 2020; Dyson et al., 2017; Zhang et al., 2022), the initiation of ciliary disassembly and cell cycle re-entry (Phua et al., 2017), and neural patterning during early brain development (Hasenpusch-Theil et al., 2020; Schembs et al., 2022).

Genetic loss-of-function variants in *INPP5E* lead to a range of disorders in patients, from non-syndromic retinitis pigmentosa (RP) (Sangermano et al., 2021; Whiting et al., 2024), where only the retina is affected to multi-organ syndromic conditions including Joubert syndrome (JS) (Bielas et al., 2009; Shetty et al., 2017) and MORM syndrome (Jacoby et al., 2009). Although the majority of the pathogenic variants cluster in the phospho-catalytic domain of *INPP5E*, and when tested affect its localization to the cilium (Whiting et al., 2024), there is no clear genotype–phenotype correlation between location or type of variants and phenotypic outcome in individuals with ciliopathies (Sangermano et al., 2021; Whiting et al., 2024). This suggests that there are other factors involved that cause the different presentations in ciliopathies, such as loss of association to other proteins within a protein complex or signaling module. Previous studies have identified several direct interactors of INPP5E including the RP-associated RPGR (Rao et al., 2016; Vössing et al., 2021), the small GTPase ARL13B (Humbert et al., 2012; Nozaki et al., 2017; Qiu et al., 2021), and the prenyl-binding protein PDE6δ (Fansa et al., 2016; Humbert et al., 2012; Thomas et al., 2014), but a comprehensive proteomics study of INPP5E has not been completed to date.

Here, we utilize a comprehensive proximity labeling proteomics approach to broaden the proximity proteome of INPP5E and identify new proteins and functional modules that INPP5E might be regulating, which in turn could be affected in individuals with variants in *INPP5E*. In doing so, we generated a dataset of 332 highly significant proteins, including known interacting partners of INPP5E, known ciliary proteins and several proteins not yet linked to the cilia or INPP5E. From this list of proteins, we identified the endocytic adaptor EH-domain binding protein 1 (EHBP1) owing to its high significance in the dataset and its previous implication in RP (Borràs et al., 2013), suggesting that this is a novel ciliary protein, which could provide insights into the retinal pathology. Next, we used human cell lines and retinal organoids derived from induced pluripotent stem cells (iPSCs) (Johnstone et al., 2018; Schembs et al., 2022; Selvaraj et al., 2018; Vasistha et al., 2019) to determine the localization of EHBP1 in different primary cilia. In doing so, we show that EHBP1 localizes to the basal body and ciliary compartment of the primary cilium and to the rudimentary outer segment membrane of photoreceptors. Dysfunction of INPP5E, either due to pathogenic variants in fibroblasts from individuals with non-syndromic RP or loss-of-function variants in retinal organoids, causes mislocalization of EHBP1 in primary cilia and photoreceptors. Taken together, these findings suggest that EHBP1 acts in a ciliary functional module, also in human photoreceptors, that is regulated by INPP5E.

## RESULTS

### Identification of EHBP1 as a potential novel regulator of INPP5E

**Fig. 1.**
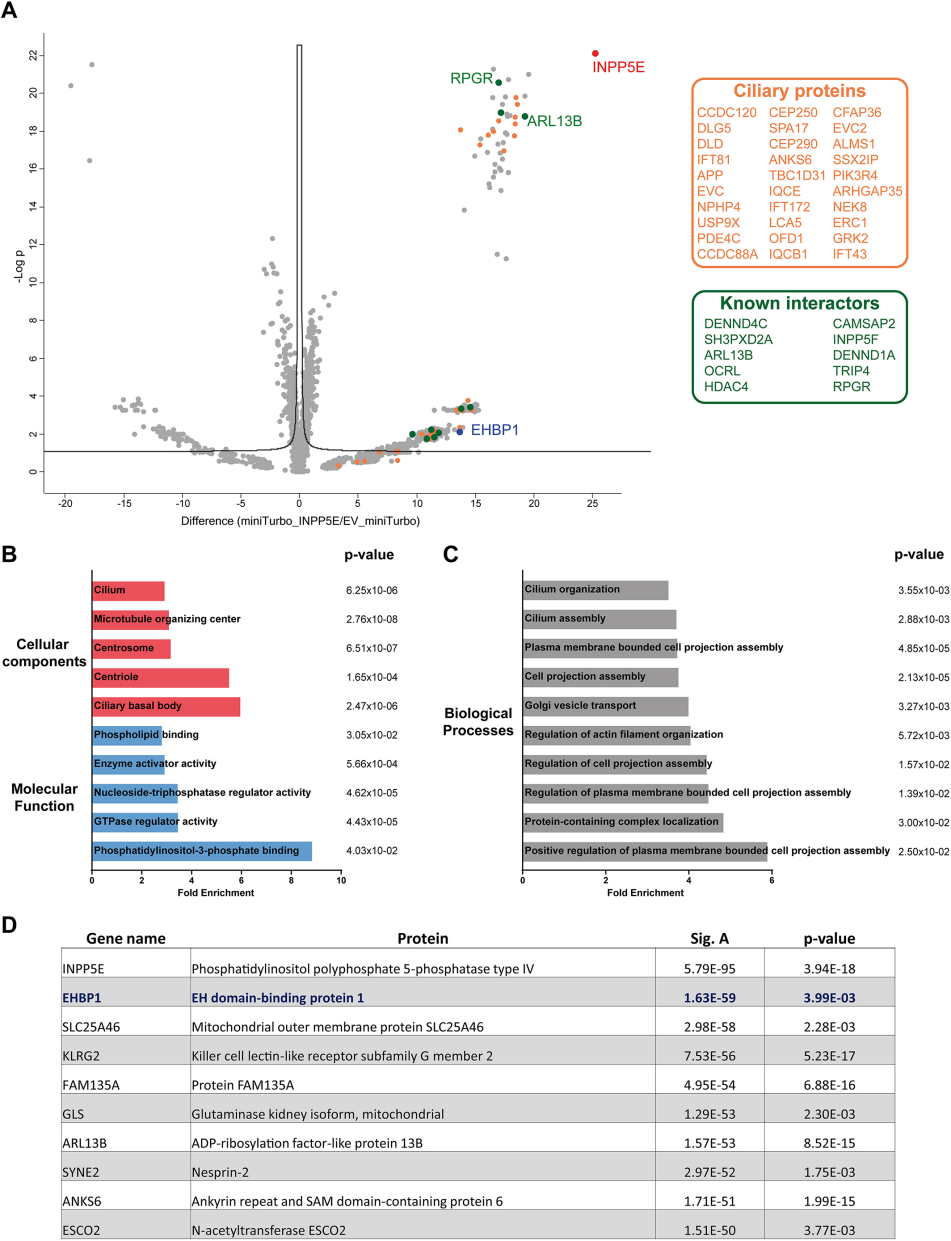
**Identification of EHBP1 through proximity proteomics of INPP5E.** (A) Volcano plot visualizing significantly enriched proteins in the miniTurbo-INPP5E dataset, plotted as the difference of miniTurbo-INPP5E and EV-miniTurbo against the negative logarithmic *P*-value of a one-tailed unpaired Student's *t*-test. Proteins of interest are highlighted including the bait protein (INPP5E, red), known ciliary proteins (orange), known interactors of INPP5E (green) and proteins of interest (EHBP1, blue). Results from six replicates. (B,C) Gene Ontology enrichment analysis for cellular components (B), molecular function (B) and biological processes (C) was performed to identify the top 5 or 10 significantly enriched terms in each category. (D) Top 10 Tier 1 enriched proteins in the miniTurbo-INPP5E dataset.

To gain further insights into the function of INPP5E and to generate a comprehensive interactome of INPP5E, we performed miniTurbo-based proximity-labeling proteomics. To do this, we first generated IMCD3 Flp-In cells that stably expressed either miniTurbo-INPP5E or the miniTurbo-empty vector (EV) plasmid, which were then subjected to biotin labeling, streptavidin pulldown and mass spectrometry. As we are only interested in proteins in close proximity to INPP5E, any proteins significantly enriched in the EV control line were discarded. The identified proteins were further classified into tiers based on significance. Tier 1 proteins had corrected *P*-values of <0.01 as well as a significance A (SignA) outlier test of <0.01. Tier 2 proteins included proteins that only had SignA test of <0.01. This resulted in a final dataset of 332 highly significant proteins, consisting of 212 Tier 1 proteins and 121 Tier 2 proteins (Table S2). Within the dataset, several known interactors of INPP5E were present, including ARL13B, RPGR, OCRL, INPP5F and HDAC4. Additionally, 30 known ciliary proteins were highly significant in the dataset, including CEP290, NPHP4, OFD1, ALMS1 and IFT172 (Fig. 1A).

Gene ontology (GO) enrichment analysis was performed to confirm both the functional role of INPP5E, through Biological Process (GO-BP) and Molecular Function (GO-MF) GO enrichment, and the localization of INPP5E, using Cellular Component (GO-CC) enrichment. In the top 5 GO-CC terms, ‘ciliary basal body’, ‘centrosome’ and ‘cilium’ were significantly enriched, and ‘phosphatidylinositol-3-phosphate binding’ and ‘phospholipid binding’ were present in the top 5 GO-MF terms (Fig. 1B). Similarly, many expected terms associated with known ciliary properties of INPP5E were present in the top 10 GO-BP terms, including ‘regulation of plasma membrane bound cell projection assembly’, ‘Golgi vesicle transport’, ‘cilium organization’ and ‘regulation of actin filament organization’ (Fig. 1C). Altogether, the results from the proximity labeling experiment yielded a high-confidence dataset that was consistent with previously known functions and interactors of INPP5E.

Interestingly, the most significantly enriched protein in the dataset was the EH-binding protein 1 (EHBP1) (Fig. 1D), a protein which has been described to bind PI(4,5)P_2_ (Wang et al., 2016) and to regulate vesicle trafficking by coupling endocytic vesicles to the actin cytoskeleton (Guilherme et al., 2004). Moreover, recent studies have shown that EHBP1 is an effector molecule for several RAB8 family members (Rai et al., 2016, 2020), including RAB8A, a protein important for ciliogenesis (Knödler et al., 2010), and RAB10, a ciliary-associated protein that plays a role in vesicle trafficking and protein transport (Babbey et al., 2010; Liu et al., 2025; Wang et al., 2016). However, to date, EHBP1 has not been studied in the context of the primary cilium.

### EHBP1 localizes to the primary cilium in human-derived cell lines

**Fig. 2.**
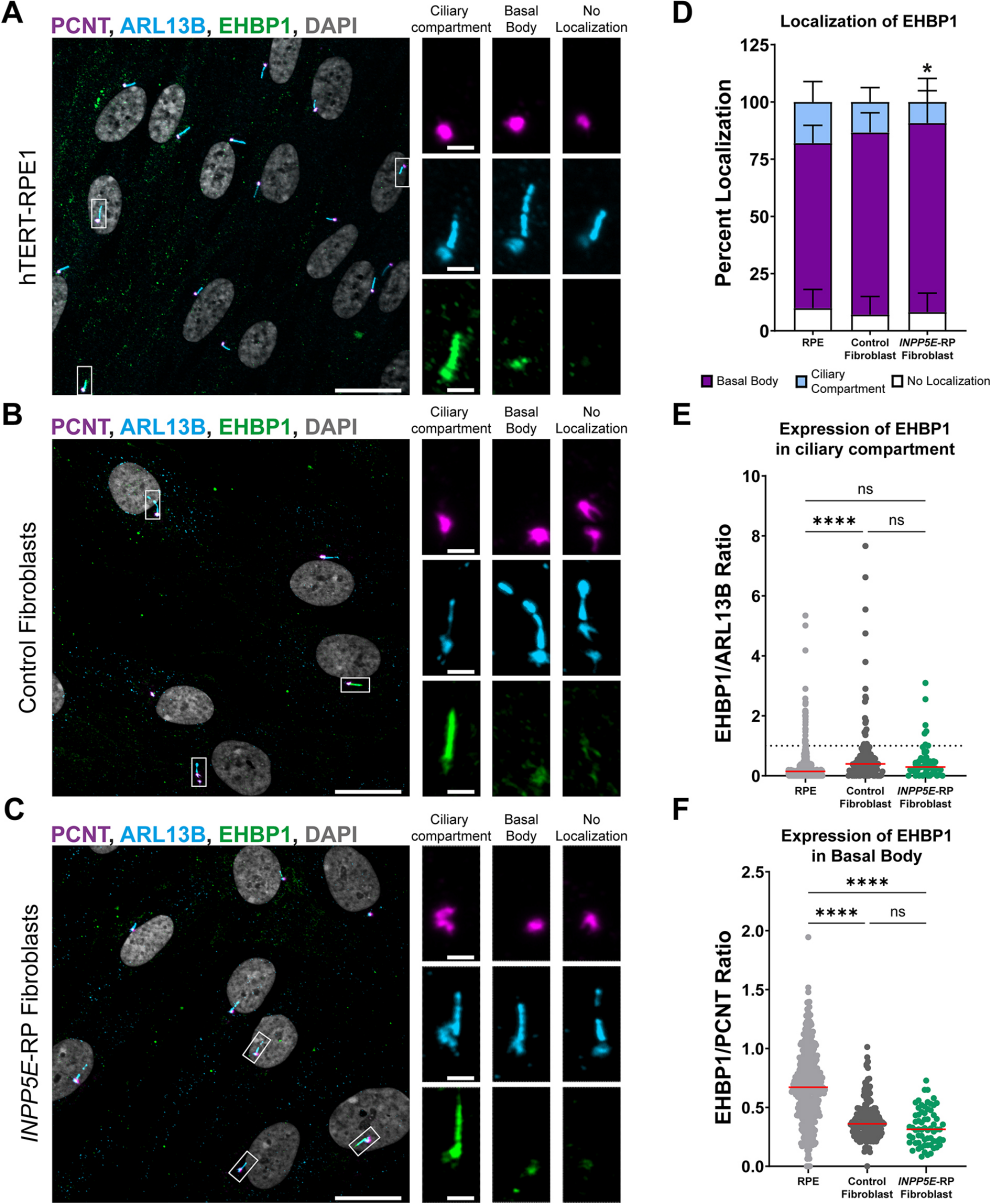
**EHBP1 localizes to the basal body and ciliary compartment of the primary cilium.** (A–C) Immunofluorescence of RPE, control fibroblasts and *INPP5E*-RP fibroblasts stained with antibodies against pericentrin (PCNT, magenta), ARL13B (blue), EHBP1 (green) and DAPI (gray). Right-hand panels show enlarged cilia representative of EHBP1 expression at the ciliary compartment, basal body, or no ciliary localization from the areas marked by the boxes. Scale bars: 20 µm (main images); 2 µm (enlargements). (D) Quantification of EHBP1 localization within the cilium as percentage localized to the basal body (purple), ciliary compartment (green) or no localization to the cilium (white). *n*=20 images over three replicates. (E) Expression of EHBP1 in the ciliary compartment of cilia as the ratio of EHBP1 intensity/ARL13B intensity. *n*=at least 100 cilia over three replicates. (F) Expression of EHBP1 at the basal body as a ratio of EHBP1 intensity/PCNT intensity. *n*=at least 100 cilia over three replicates. All error bars represent s.d. Significance was determined by one-way ANOVA with a post-hoc Tukey's test (*P=0.0150; ****P<0.0001; ns, not significant).

To validate EHBP1 as a potential ciliary protein, we utilized immunocytochemistry and immunofluorescence in human skin-derived fibroblast cells, human RPE cells and mouse IMCD3 cells. In both RPE and fibroblast cells, EHBP1 is present at the primary cilium, localizing primarily to the basal body (RPE, 75% ARL13B^+^ cilia; fibroblasts, 80% ARL13B^+^ cilia) and less frequently to the ciliary compartment (RPE, 16% ARL13B^+^ cilia; fibroblasts, 13% ARL13B^+^ cilia), with the remainder of cilia having no EHBP1 expression (RPE, 9% ARL13B^+^ cilia; fibroblasts, 7% ARL13B^+^ cilia) (Fig. 2A,B,D). Owing to the high abundance of EHBP1 when it was present in the ciliary compartment, the intensity of EHBP1 at the basal body and within the cilium was measured and analyzed as a ratio compared to the intensity of the ciliary markers PCNT or ARL13B, respectively. RPE cells had a significant increase of EHBP1 expression at the basal body (RPE, 0.68±0.31 EHBP1/PCNT ratio; Fibroblasts, 0.40±0.17 EHBP1/PCNT ratio; mean±s.d.) (Fig. 2F), although expression within the ciliary compartment was less than in fibroblast cells (RPE, 0.36±0.64 EHBP1/ARL13B ratio; fibroblasts, 0.73±1.2 EHBP1/ARL13B ratio) (Fig. 2E).

In fibroblasts, the difference of intensity when EHBP1 enters the cilium is more dramatic than that of RPE cells. Moreover, EHBP1 does not ubiquitously move to the cilium in all cell types. In mouse IMCD3 cells, EHBP1 is expressed at the cell membrane, with no ciliary localization seen (Fig. S1D). Additionally, western blot analysis of EHBP1 in IMCD3 cells shows decreased expression and increase in non-specific binding compared to human RPE cells (Fig. S1E). To confirm the specificity of EHBP1 localization in human RPE cells, siRNA knockdown of EHBP1 was performed. Expression of EHBP1 was decreased in the ciliary compartment and basal body of siRNA treated cells compared to control samples, demonstrating the specificity of the EHBP1 antibody in human cells (Fig. S2).

### Ciliary localization of EHBP1 is not dependent on sonic hedgehog signaling

We next sought to determine the stimulus that causes EHBP1 to move from the basal body to the ciliary compartment. As INPP5E directly regulates sonic hedgehog (SHH) signaling by restricting PI(4,5)P_2_ levels in the ciliary membrane (Garcia-Gonzalo et al., 2015), and because EHBP1 has been shown to associate with PI(4,5)P_2_-enriched membranes (Wang et al., 2016), we hypothesized that EHBP1 localization in the ciliary compartment occurs upon stimulation of SHH signaling. To assess this, we utilized the SHH agonist SAG to stimulate the pathway in RPE cells. When SHH is stimulated, the G-protein-coupled receptor smoothened (SMO) moves from the base of the cilium to the ciliary compartment. Cells treated with vehicle (DMSO) had 18.5% of cilia with SMO expression in the ciliary compartment, whereas 73% of cilia had SMO expression after SAG treatment (Fig. S3). However, stimulation of SAG did not affect the localization of EHBP1 in the cilium (Fig. S3).

### Treatment with Cytochalasin D affects EHBP1 ciliary localization and expression

**Fig. 3.**
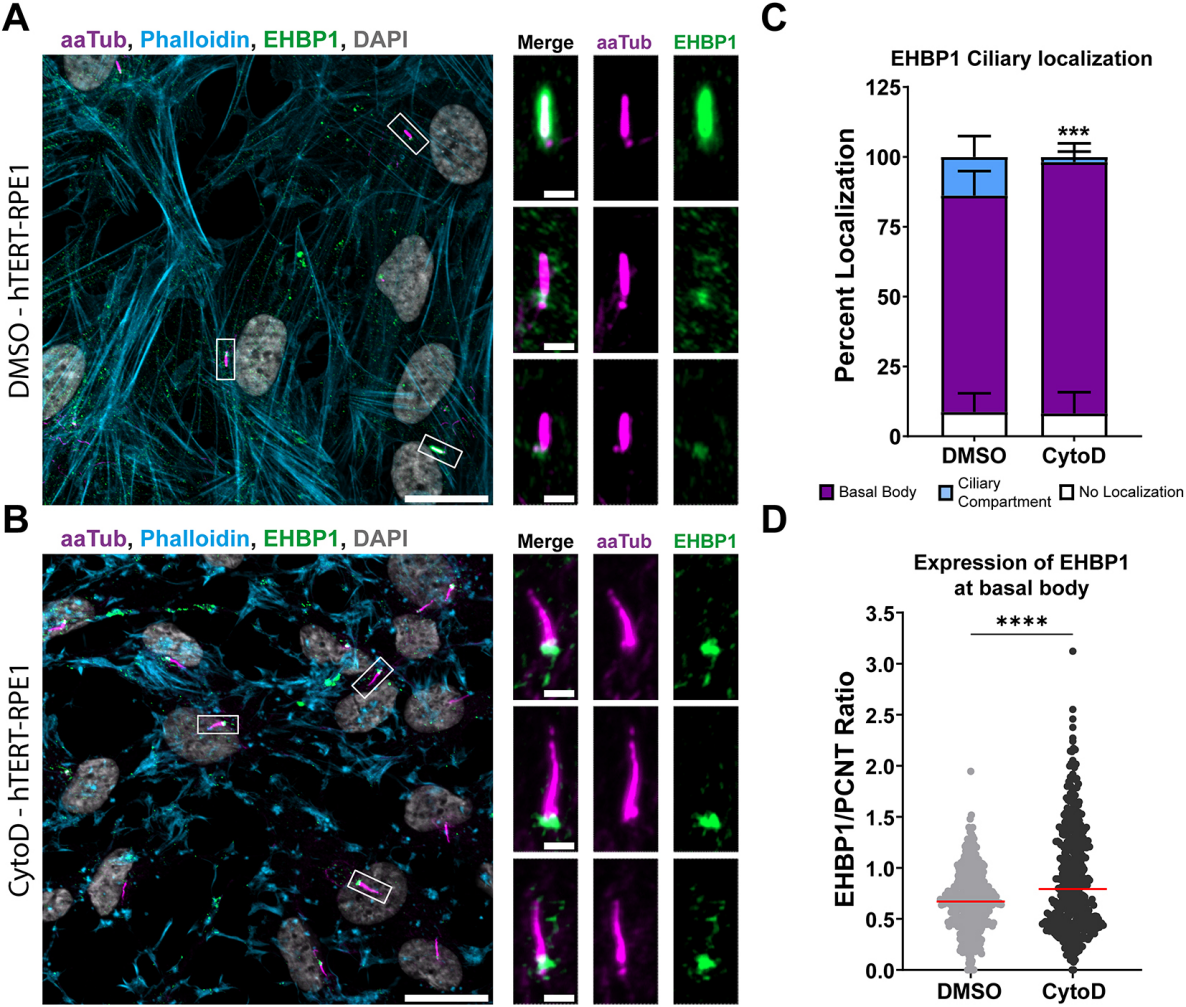
**Treatment with CytoD affects EHBP1 ciliary localization.** (A,B) Immunofluorescence of RPE cells treated with DMSO vehicle (A) or 10 µM Cytochalasin D (B). Cells are stained against acetylated α-tubulin (aaTub, magenta), phalloidin (blue), EHBP1 (green), and DAPI (gray). Scale bars: 20 µm and 2 µm (cutouts). (C) Quantification of EHBP1 ciliary localization in the basal body and ciliary compartment (*n*=8 images over two replicates). (D) Quantification of EHBP1 expression at the ciliary basal body for cells treated with DMSO or CytoD, measured as the ratio of EHBP1 intensity/PCNT intensity. *n*=at least 100 cilia over three replicates. All error bars represent s.d. Significance was determined by one-way ANOVA with a post-hoc Tukey's test (***P=0.0009; ****P<0.0001).

As EHBP1 is known to bind actin via its central CH domain, and has been shown to play an important role in regulating the actin cytoskeleton (Rai et al., 2020), we hypothesized that its localization and expression would be altered by treating RPE cells with the actin poison Cytochalasin D (CytoD). Cilia in CytoD-treated cells increased in length, as previously reported (Fig. S4) (Kim et al., 2015) and the actin cytoskeleton structure was significantly altered after CytoD treatment (Fig. 3A). Moreover, EHBP1 localization and expression was significantly altered when actin dynamics were affected by CytoD, causing EHBP1 to accumulate at the basal body (DMSO: 77% ARL13B^+^ cilia, CytoD: 90% ARL13B^+^ cilia) and rarely enter the cilium (DMSO: 13% ARL13B^+^ cilia, CytoD: 1.8% ARL13B^+^ cilia) (Fig. 3B). Interestingly, the expression of EHBP1 at the ciliary basal body was also significantly increased in CytoD-treated cells (DMSO, 0.68±0.31 EHBP1/PCNT ratio; CytoD, 0.89±0.52 EHBP1/PCNT ratio; mean±s.d.) (Fig. 3C,D). To determine whether the change in localization and expression were exclusive to EHBP1, we analyzed the expression of INPP5E and PI(4,5)P_2_ – a known interactor of EHBP1 – after CytoD treatment. However, no alteration in localization or expression was seen for either protein (Fig. S4).

### EHBP1 localizes to the rudimentary outer segment membrane of photoreceptors in mature retinal organoids

**Fig. 4.**
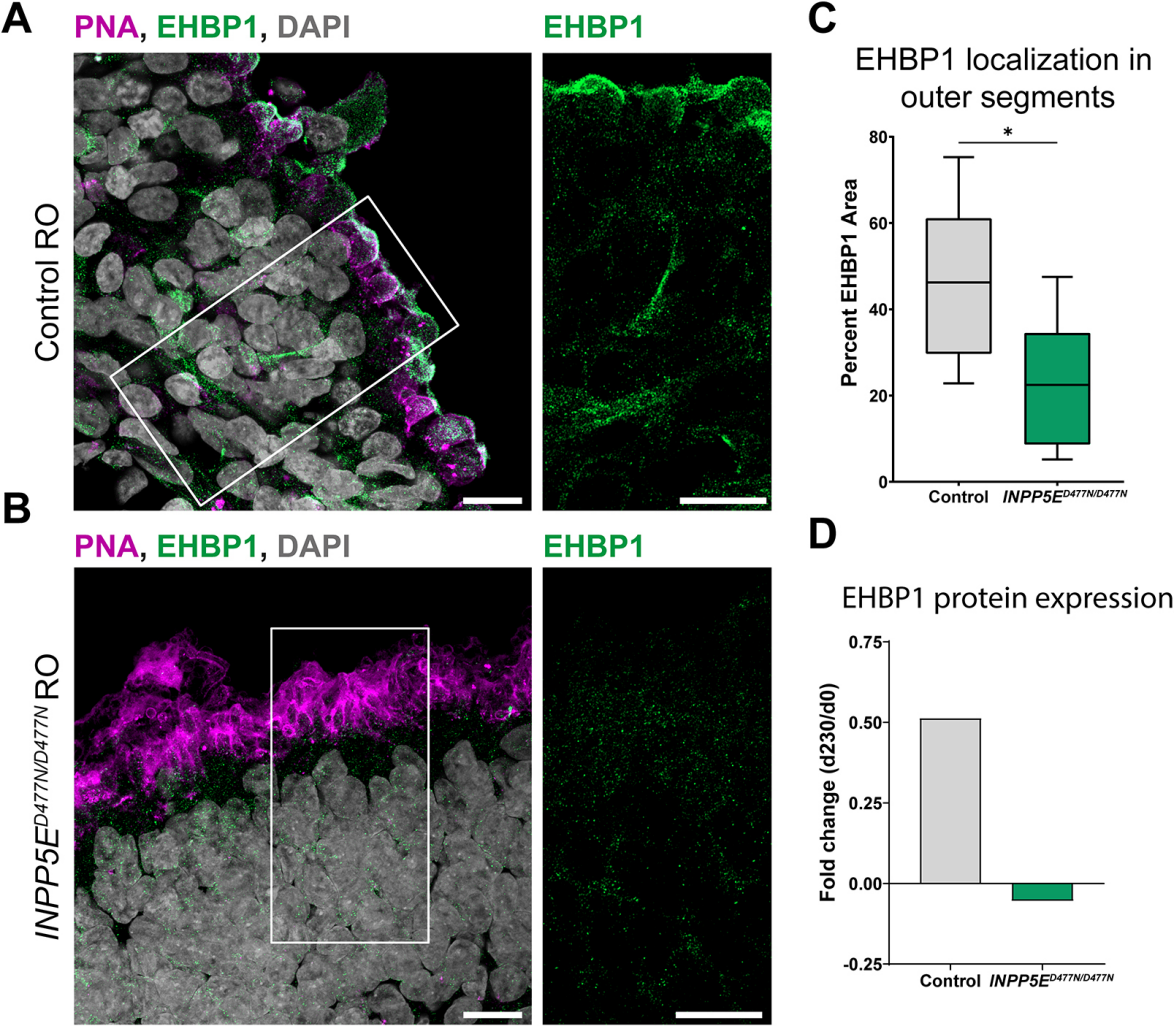
**EHBP1 localizes to the photoreceptor outer membrane of d230 retinal organoids.** (A,B) Immunofluorescence of d230 control and *INPP5E^D477N/D477N^* retinal organoids, stained against antibodies for the photoreceptor outer membrane (PNA, magenta), EHBP1 (green) and DAPI (gray). Scale bars: 20 µm (main images); 10 µm (enlargements). (C) Quantification of EHBP1 expression in photoreceptor outer membranes, as the percentage EHBP1-positive area in PNA stained outer segment (*n*=6 organoids over two differentiations). The box represents the 25–75th percentiles, and the median is indicated. The whiskers show the minimum to maximum values. (D) Fold change of EHBP1 protein expression in d230 compared to undifferentiated iPSCs, as measured by mass spectrometry (mean; *n*=8–10 organoids). Significance was determined by an unpaired two-tailed *t*-test (*P=0.0366).

Recent work has highlighted the importance of INPP5E in regulating protein trafficking within the photoreceptors (Gupta et al., 2025), a specialized sensory cilium with a highly modified axoneme. EHBP1 has, to date, not been studied in the context of the retina; however, a previous study identified a variant in EHBP1 in an individual with autosomal dominant RP (Borràs et al., 2013), suggesting a potential role of EHPB1 in photoreceptors. We utilized control retinal organoids to determine the localization of EHBP1 in the developing retina (Whiting et al., 2026). In mature [day 230 (d230)] retinal organoids, photoreceptors with rudimentary outer segments develop along the outer edge of the organoid (Gonzalez-Cordero et al., 2017). Here, EHBP1 localizes primarily to the outermost membrane of the photoreceptors, colocalizing with the photoreceptor outer segment membrane marker peanut agglutinin (PNA) (Fig. 4A), and shows no clear colocalization with ARL13B (Fig. S6).

During early stages of retinal development [day 35 (d35)], EHBP1 is expressed along the outer edge of the developing organoids. In rare instances, EHBP1 colocalizes with ARL13B in the inner nuclear layer of the developing organoids, however due to the low numbers this cannot be quantified (Fig. S5).

### Alterations in INPP5E affect EHBP1 expression in primary cilia and retinal organoids

As EHBP1 was identified in the proximity proteome of INPP5E, we next sought to understand the extent INPP5E interacts with EHBP1. A co-immunoprecipitation (co-IP) of INPP5E and EHBP1 showed that there was no direct interaction between the two proteins (Fig. S1); however, it is possible that INPP5E interacts with EHBP1 indirectly though shared functional protein modules. For this we utilized fibroblasts derived from an individual with non-syndromic RP containing compound heterozygous variants in *INPP5E* (p.Q506* and p.A283T, *INPP5E*-RP), described previously (Whiting et al., 2024) to determine whether INPP5E is involved in EHBP1 ciliary localization. In these cells, EHBP1 localization was slightly affected, localizing to the basal body in 68% of ARL13B^+^ cilia, the ciliary compartment in 7% cilia, while 25% of ARL13B^+^ cilia showed no EHBP1 compared to control fibroblasts (basal body, 80%; ciliary compartment, 13%; no ciliary localization, 7%) (Fig. 2C,D). Together, this suggests a mild localization defect of EHBP1 when INPP5E is affected. To determine whether *INPP5E* variants also affected the expression levels of EHBP1, the intensity of EHBP1 expression at the basal body and within the ciliary compartment was measured. At the basal body, EHBP1 expression was not altered compared to control cells (control, 0.40±0.17 EHBP1/PCNT ratio; *INPP5E*-RP, 0.34±0.15 EHBP1/PCNT ratio; mean±s.d.) (Fig. 2F). In the ciliary compartment there is a slight, but not significant, difference between control and *INPP5E*-RP cells (control, 0.73±1.2 EHBP1/ARL13B ratio; *INPP5E*-RP, 0.48±0.60 EHBP1/ARL13B ratio) (Fig. 2E). Together, these results suggest that INPP5E does affect the localization of EHBP1 to and within the cilium, but not the expression levels of EHBP1.

To determine whether INPP5E also regulates EHBP1 localization in photoreceptors, immunofluorescence in *INPP5E^D477N/D477N^* organoids was performed. In loss-of-function organoids, EHBP1 no longer localizes to the rudimentary outer segment membrane of photoreceptors, with the percentage of positive area of EHBP1 expression in PNA^+^ outer segment membranes decreasing from 46.5±18% in control retinal organoids to 22.9±15% (mean±s.d.) in *INPP5E^D477N/D477N^* retinal organoids (Fig. 4B,C). However, EHBP1 expression within the outer nuclear layer (ONL) and inner nuclear layer (INL) was not affected by the loss of INPP5E (Fig. S6). Additionally, protein expression of EHBP1 in d230 organoids was increased 0.5-fold in control d230 organoids compared to undifferentiated iPSCs. In *INPP5E^D477N/D477N^* organoids, EHBP1 expression was marginally downregulated at d230 compared to undifferentiated iPSCs (Fig. 4D).

At early stages of organoid development (d35), there was no significant alteration in EHBP1 localization in the developing ONL (control, 10.7% EHBP1^+^ area; *INPP5E^D477N/D477N^*, 6.4% EHBP1^+^ area) or INL (control, 2.8% EHBP1^+^ area; *INPP5E^D477N/D477N^*, 4.3% EHBP1^+^ area) (Fig. S5). Similarly, no difference was seen in intensity of EHBP1 in ARL13B^+^ cilia [control, 470.2 arbitrary units (A.U.); *INPP5E^D477N/D477N^*, 365.1 A.U.], or in PCNT^+^ basal bodies (control, 433.1 A.U.; *INPP5E^D477N/D477N^*: 357.5 A.U.) (Fig. S5). However, unlike control organoids, EHBP1 did not appear to move to the cilium in any instances in *INPP5E^D477N/D477N^* retinal organoids.

## DISCUSSION

In this study, we utilized proximity labeling proteomics to generate a comprehensive proximity proteome of INPP5E in order to identify new proteins and functional modules of the protein. In doing so, we identified the endocytic vesicle trafficking protein EHBP1 as a new ciliary protein that might be regulated by INPP5E.

EHBP1 is a vesicle-associated protein that has been previously shown to play an important role in GLUT4 transport through endocytosis and which connects endocytic vesicles to the actin cytoskeleton through direct binding to its CH domain (Guilherme et al., 2004). Moreover, EHBP1 acts in a complex with RAB10 and EHD2 to regulate lipid droplet degradation during lipophagy (Li et al., 2016), and directly interacts with several phosphoinositides, including PI(3)P, PI(5)P and PI(4,5)P_2_ (Rai et al., 2020; Wang et al., 2016). Additionally, EHBP1 has been identified as an interactor with the intraflagellar transport (IFT) protein IFT57 (Huttlin et al., 2021), which is important for anterograde trafficking of proteins within the primary cilium. Previous work has identified the protein EH binding protein 1-like 1 (EHBP1L1) as a ciliary protein that localizes to the ciliary sheath and plays a role in regulating cilia length through the regulation of the actin network (Iwano et al., 2023). As EHBP1L1 and EHBP1 share several domain structures and have a 66% similarity, we sought to determine whether EHBP1 also localizes to the cilium.

Using antibody-based immunofluorescence, we determined that EHBP1 localizes to the basal body of cilia 70–80% of the time, with 10–20% of cilia having expression within the ciliary compartment in human fibroblast and RPE cells. However, the intensity and expression patterns of EHBP1 at both the basal body and ciliary compartment differ between fibroblasts and RPE cells. In mouse IMCD3 cells, EHBP1 did not localize to the basal body or the cilium but rather to the cell membrane. This is likely due to differences in antibody specificity between the mouse and human cells, as western blot analysis showed an increase of non-specific binding in mouse cells when compared to human cell types. Taken together, these data suggest a tissue-specific role for EHBP1 within the cilium. Moreover, the multiple localizations of EHBP1 at and within the cilium suggests a complex function of EHBP1. It is possible that EHBP1 is accumulating at the basal body until a specific signal triggers the transport of EHBP1 into the cilium. Here, we showed that EHBP1 localization is not affected by the activation of the SHH pathway; however, disruption of the actin cytoskeleton by CytoD causes EHBP1 accumulation at the basal body and prevents entry into the ciliary compartment. Previous work has shown that actin disruption via CytoD causes cilia length to increase (Drummond et al., 2018; Kim et al., 2015; Smith et al., 2020), and hypothesized that actin is regulating cilia length by providing a diffusion barrier that affects vesicle trafficking and anterograde transport. The increased accumulation of EHBP1 at the base of the cilium could lead to an increase of vesicle trafficking into the cilium, thus leading to longer cilia.

Although EHBP1 has previously been studied in the context of cell function, its localization and function in specialized tissues remains unclear. A previous study of individuals with autosomal dominant RP identified a variant in *EHBP1* classified as probably damaging (Borràs et al., 2013); however, EHBP1 has not been studied extensively in the context of the human retina. In mature retinal organoids, EHBP1 is expressed at the rudimentary outer segment membrane of the developing photoreceptors. Although the function remains unclear, it is possible that EHBP1 is important for regulating the membrane structure and biogenesis of photoreceptor outer segments by regulating the interaction between PI(4,5)P_2_-rich photoreceptor membranes and the actin cytoskeleton. This hypothesis would match very recent analysis of a conditional rod-specific *Inpp5e*-knockout mouse model, where loss of Inpp5e was found to disrupt the actin cytoskeleton in the retina (Gupta et al., 2025).

As EHBP1 was identified in the proximity proteome of INPP5E, we next sought to understand the relationship between the two proteins beyond merely being in close vicinity to each other, as they both act on PI(4,5)P_2_. In dermal fibroblasts derived from individuals with *INPP5E-*linked RP (Whiting et al., 2024), EHBP1 still localizes largely to the cilium; however, EHBP1 localizes less frequently to the ciliary compartment in the *INPP5E*-RP fibroblast cells than in controls. This could indicate that INPP5E is not involved in recruitment of EHBP1 to the basal body but does affect the localization of EHBP1 into the ciliary compartment. However, as the expression of EHBP1 is not completely ablated by the dysfunction of INPP5E, multiple factors are likely involved in regulating the expression of EHBP1 in the ciliary compartment.

In *INPP5E^D477N/D477N^* organoids, EHBP1 localization is not affected during early stages of retinal organoid development, but in mature organoids EHBP1 expression in the photoreceptors is almost completely ablated. Previous work has hypothesized that INPP5E plays an important role in regulating protein trafficking in the connecting cilium of photoreceptors (Gupta et al., 2025). Additionally, it has been shown that retrograde trafficking of IFT is defective in mouse photoreceptors containing a conditional knockout of *Inpp5e* (Sharif et al., 2021). It is possible that INPP5E plays a role in trafficking EHBP1 to the photoreceptor, and that the loss of photoreceptor connecting cilium-localized INPP5E leads to the mislocalization of EHBP1 in the photoreceptor membrane. Furthermore, *INPP5E^D477N/D477N^* organoids have longer photoreceptor outer segments compared to control organoids at d230 (Whiting et al., 2026). If EHBP1 is important in regulating the stability of the photoreceptor outer segment membrane, the alteration in localization could be playing a role in the lengthening of photoreceptors in *INPP5E^D477N/D477N^* organoids.

In conclusion, through proximity labeling proteomics of INPP5E, we identified EHBP1 as a new ciliary protein with localization at both the base and the ciliary compartment, and with cell line-specific expression. Moreover, EHBP1 localizes at the rudimentary photoreceptor outer segment membrane of mature retinal organoids, suggesting that EHBP1 functions within the photoreceptor sensory cilium and that its function is regulated by INPP5E. Together, this provides further insights into disease pathogenesis of retinal ciliopathies caused by mutations in *INPP5E*, and implicates EHBP1 as a candidate protein for retinitis pigmentosa and other retinal ciliopathies.

## MATERIALS AND METHODS

### Cell culture

Murine inner medullary collecting duct 3 (IMCD3; a kind gift from Dr Maxence Nachury, University of California San Francisco, USA) and human TERT-immortalized retinal pigment epithelial 1 (RPE; ATCC) cells were cultured in Dulbecco's modified Eagle's medium (DMEM, Sigma-Aldrich, D0819) with F12 (Ham's Nutrient Mixture F12, Sigma-Aldrich, N6658) in a 1:1 ratio supplemented with 10% fetal bovine serum (FBS, Gibco, 10270106), 1% sodium pyruvate (Sigma-Aldrich, S8636) and 1% penicillin-streptomycin (Sigma-Aldrich, P4333). IMCD3 Flp-in cells were cultured in the same DMEM:F12 mixture at a 1:1 ratio containing the same supplements with the addition of 100 µg/ml Zeocin (Life Technologies; R25005) prior to transfection or with 400 µg/ml hygromycin (Sigma-Aldrich; H3274) after transfection. Human skin-derived fibroblast cells were cultured in DMEM (DMEM, Sigma-Aldrich, D0819) with 20% FBS, 1% sodium pyruvate (Sigma-Aldrich, S8636) and 1% penicillin-streptomycin (Sigma-Aldrich, P4333). Control fibroblasts and fibroblast lines from individuals with *INPP5E-*linked RP were as described previously (Whiting et al., 2024). iPSC lines containing a homozygous D477N/D477N mutation in INPP5E, an enzymatically null loss-of-function mutation hereby referred to as *INPP5E^D477N/D477N^*, and its corresponding isogenic control iPSC line were a kind gift from Dr Thomas Theil (University of Edinburgh, UK) and characterized previously (Schembs et al., 2022). Cells were cultured in six-well plates coated with Matrigel (Corning, 734-0269) in mTesR1 medium (Stemcell Technologies, 85850) containing 1% penicillin-streptomycin (Sigma-Aldrich, P4333). All cells were incubated at 37°C with 5% CO_2_ and regularly tested for mycoplasma.

### Plasmid construction

The full-length human *INPP5E* (ENSG00000148384), *EHBP1* (ENSG00000115504) and *PDE6D* (ENSG00000156973) were previously cloned into a Gateway^TM^ pDONR^TM^201 Vector according to the manufacturer's instructions (Thermo Fisher Scientific). The plasmid sequences were further subcloned into either pgLAP1-miniTurbo plasmid, p3xFlag plasmid, or pGCS-N2(3xHA) plasmid via LR reactions as described previously (Aslanyan et al., 2023). The GFP–EHBP1 plasmid was a kind gift from Dr Amrita Rai (Department of Structural Biochemistry, Max Planck Institute of Molecular Physiology, Dortmund, Germany).

### Cell line generation

Utilizing the Thermo Fisher Scientific Flp-In^TM^ technology, IMCD3 cells were stably transfected to express miniTurbo-INPP5E according to the manufacturer's protocol (Thermo Fisher Scientific). In short, IMCD3 cells were co-transfected with expression vectors encoding miniTurbo-INPP5E and pOG44. Stably transfected cells were selected using DMEM:F12 selection medium containing 400 µg/ml hygromycin (Sigma-Aldrich; H3274), and the generated cell lines were validated using immunofluorescent microscopy and western blotting.

### Immunofluorescence staining

IMCD3, RPE and fibroblast cells were seeded on 18-mm diameter glass coverslips and grown until 80% confluency. To stimulate ciliogenesis, cells were serum starved for 48 h in their respective medium containing 0% (IMCD3, RPE cells) or 0.2% (fibroblasts) FBS. Cells were then fixed for 20 min in 2% paraformaldehyde (PFA; Thermo Fisher Scientific, J19943.K2) in PBS and permeabilized in 1% Triton X-100 (Sigma-Aldrich, T8787) in PBS for 5 min. Cells were blocked in 2% bovine serum albumin (BSA; Sigma-Aldrich, A6003) and incubated with primary antibodies in 2% BSA for 1.5 h at room temperature (RT) or overnight at 4°C. The cells were washed extensively with PBS before incubation with secondary antibodies in 2% BSA for 45 min at RT. Coverslips were embedded in Fluoromount-G with DAPI (Southern Biotech, 0100-20) on microscopic glass slides. Antibodies and dilutions used in this study are listed in Table S1. Images were taken using a Zeiss Axio Imager Z2 fluorescence microscope equipped with an Apotome or Zeiss LSM900 laser scanning microscope with Airyscan using 63× oil-immersion objective magnification. Final images are represented as maximum projection of *Z*-stacks.

### Sonic hedgehog signaling and actin poison assays

RPE cells were used to assess the effect of sonic hedgehog (SHH) signaling and actin poisons on EHBP1 localization and expression. Cells were seeded on 18-mm diameter glass coverslips and grown until 100% confluent, at which point medium was replaced with 0% FBS starvation medium for 48 h. For SHH activation, cells were incubated overnight in 500 nM of the SHH agonist SAG (Tocris, 4366) for 24 h post starvation. For the actin poison assay, cells were incubated in 1 µM Cytochalasin D (Sigma-Aldrich, C8273) for 1 h. Following incubation, cells were fixed and stained for immunofluorescent analysis as described above.

### siRNA-mediated knockdown of EHBP1

To confirm EHBP1 antibody specificity, RPE cells were seeded in a six-well plate including 12 mm glass coverslips to analyze protein expression using western blot and immunofluorescent analysis. In short, siPOOLS (siTOOLS Biotech) targeting human EHBP1 or the negative control siPOOL [non-targeting (NT) control] were transfected using Lipofectamine RNAiMAX (Thermo Fisher Scientific) according to the instructions from siTOOLS Biotech at 5 nM concentration. The cells were cultured for 72 h to reach sufficient knockdown levels, and medium was replaced with starvation medium containing 0% FBS to induce ciliation 24 h after transfection. The coverslips were fixed for immunofluorescent analysis and cell pellets were collected and lysed for western blot analysis, as described.

### iPSC-derived retinal organoid differentiation

Control and *INPP5E^D477N/D477N^* organoids were differentiated to retinal organoids as previously described (Georgiou et al., 2020; Kuwahara et al., 2015). In brief, cells were dissociated as single cells in accutase (Sigma, A6964), resuspended in mTesR1 medium with 10 µM Y-27632 (ROCK inhibitor, Sigma, Y0503) and seeded at 7000 cells per well in U-bottom ultralow adhesion 96 well plates (Corning, 7007). After 48 h [day 0 (d0) of differentiation], 200 µl differentiation medium containing 41% IMDM (Sigma, I3390), 41% Ham's F12 (Sigma, N6658), 15% KOSR (Gibco, 10828028) 1% GlutaMAX (Gibco 35050061), 1% lipids (Gibco, 11905031), 1% penicillin-streptomycin and 0.2% 1-thioglycerol (Sigma-Aldrich, M6145) was added per well, with subsequent half medium changes every 2 days after. On day 6, a pulse of 2.25 nM BMP4 (Sigma, SRP3016) was added. After 18 days, the medium was changed to maintenance medium, containing DMEM:F12 (Gibco, 31330038), 10% FBS, 1% N2 (Gibco, 17502048), 1% penicillin-streptomycin, 1% GlutaMAX, 0.1 mM taurine (Sigma Aldrich, T0625), 0.25 µg/ml Amphotericin B (Gibco, 15290018) and 0.5 µM 9-cis-retinol (Sigma, R5754). Medium was changed every 3–4 days thereafter. After 120 days of differentiation, 9-cis-retinol was removed from the medium and the medium was changed two times per week until the end of the differentiation.

### Retinal organoid immunofluorescent microscopy

For immunofluorescence, organoids were fixed in 4% PFA at RT for 20 mins and cryoprotected in 30% sucrose overnight at 4°C. 2–4 organoids were embedded in OCT and frozen on dry ice. Immunofluorescence staining was performed on 12 µm cryostat sections. In brief, sections were washed with PBS to remove OCT and incubated in blocking buffer [0.3% Triton X-100, 2.5% normal goat serum (NGS; Invitrogen, 10000C) in PBS] for 1 h, before overnight incubation in primary antibody dilution buffer (0.1% Triton-X, 0.5% NGS in PBS). Slides were washed in PBS and incubated for 1 h in secondary antibody mixes diluted in PBS before mounting with Fluoromount-G. Antibodies used are listed in Table S1. Images were captured with a 63× oil immersion objective on the Zeiss LSM900 laser scanning microscope with Airyscan.

### Cell lysis, co-immunoprecipitation and western blot analysis

Cells were lysed in RIPA buffer (50 mM Tris-HCl pH 7.5, 150 mM NaCl, 1% NP-40, 0.1% SDS, 0.5% sodium deoxycholate and 1 mM EDTA) for 30 min, centrifuged (14,000 ***g***, 30 mins, 4°C), and boiled at 95°C for 5 min in NuPAGE^TM^ LDS Sample Buffer (4×) with 50 mM DTT.

For co-immunoprecipitation, HEK293 T cells (ECACC) were transfected with 3×HA–INPP5E and either 3×Flag–EHBP1, 3×Flag–PDE6D (positive control) or 3×Flag-Empty Vector (negative control) plasmid DNA using Lipofectamine 2000 (Invitrogen, 11668019). After 24 h, cells were lysed in ice-cold immunoprecipitation lysis buffer (50 mM Tris-HCl pH 7.5, 100 mM NaCl, 1% NP-40 and 10% glycerol) with cOmplete protease inhibitor cocktail (Roche, 11836170001). Samples were centrifuged 15 min at 14,000 ***g*** at 4°C, added to pre-washed FLAG beads, and incubated on a rotating wheel for 2 h at 4°C. The beads were washed three times in ice-cold immunoprecipitation lysis buffer, after which, the beads were boiled in NuPAGE^TM^ LDS Sample Buffer (1×) containing 100 mM DTT for 5 min at 95°C.

SDS-PAGE and western blotting were performed as previously described (Faber et al., 2023) using the NuPAGE system (Thermo Fisher Scientific) according to the manufacturer's instructions. Blots were imaged on an Odyssey DLx (Li-cor) and analyzed using the ImageStudio Software (LI-COR Biosciences) using antibodies listed in Table S1. Uncropped images of blots from this paper are shown in Fig. S7.

### MiniTurbo proximity labeling

Proximity labeling was performed in the miniTurbo-INPP5E cell line generated above with a previously generated miniTurbo-empty vector (EV) IMCD3 Flp-In cell line as a control. Six technical replicates per condition were performed. Cells were seeded at 15% confluency and cultured for 24 h in normal DMEM:F12 medium as described above. Ciliogenesis was stimulated for 48 h using starvation medium (0% FBS). 10 µM biotin (Sigma-Aldrich, B4501) was added to induce proximity labeling overnight. The cells were lysed and prepared for mass spectrometry as described previously (Aslanyan et al., 2023). Briefly, cells were lysed in RIPA buffer [50 mM Tris-HCl pH 7.5, 150 mM NaCl, 1% NP-40, 0.1% SDS (Life Technologies, 15553-027)] containing cOmplete protease inhibitor cocktail (Roche, 11836170001). Collected cells were briefly sonicated, and further lysed rotating at 4°C for 1 h. Lysates were centrifuged at 14,000 ***g*** for 30 min at 4°C and collected for immunoprecipitation of biotinylated substrates. Lysates were incubated for 3 h at 4°C with pre-cleared StrepTactin Superflow beads (IBA, 2-1206-025). After incubation, beads were washed with ice-cold TBS (20 mM Tris, 150 mM NaCl) and digested in trypsin digest buffer [2 mM urea, 50 mM Tris-HCl pH 7.5, 5 µg/sample trypsin (Serva, 9002-07-7)] for 1 h at 27°C and 800 rpm. The beads were washed twice in digestion buffer (2 mM urea, 50 mM Tris-HCl pH 7.5 and 1 mM DTT). The eluted peptides were incubated overnight to allow further digestion. Finally, they were snap-frozen and stored at −80°C until mass spectrometry (MS) analysis.

### Bulk proteomics of retinal organoids

Control and *INPP5E^D477N/D477N^* retinal organoids were collected and processed for proteomics at d0 (undifferentiated iPSCs) and d230 (*n*=8–12 organoids). Organoids and cells were collected and washed twice in PBS and lysed in RIPA buffer [50 mM Tris-HCl pH 7.5, 150 mM NaCl, 1% NP-40, 0.1% SDS (Life Technologies, 15553-027), 0.5% sodium deoxycholate, 1 mM UltraPure EDTA pH 8.0 (Invitrogen, 11568896)] containing cOmplete protease inhibitor cocktail (Roche, 11836170001). Samples were homogenized using the Qiagen TissueLyser III and incubated on ice for 10 mins before being centrifuged for 15 mins at 14,000 ***g*** at 4°C. Lysate concentration was measured using the Pierce BCA protein assay (Life Technologies, 23225), and aliquoted in 15 µg aliquots. Lysates were then snap frozen and stored at −80°C until MS analysis.

### Liquid chromatography-mass spectrometry analysis and data analysis

Tryptic peptides from bulk proteomics and miniTurbo proximity labeling were processed for label free high resolution LC-MS/MS approach using data-independent acquisition mode (DIA) on an Orbitrap Tribird Fusion mass spectrometer as previously described (Mattern et al., 2025; Merk et al., 2024). MS raw data files of the samples were analyzed using DIA-NN 1.8.1 (Demichev et al., 2019) in library-free mode against either the mouse database (SwissProt, May 2024, 17,144 proteins) or the human database (Swiss-Prot, May 2024, #20,401 proteins). High-accuracy spectra with a minimum precursor false discovery rate (FDR) of 0.01 and tryptic peptides were used for protein abundance label free quantification.

Statistical analysis of both proximity labeling and bulk proteomic data were calculated using Perseus (version 1.6.15.0) (Tyanova et al., 2016). Significant proteins were identified using a Significance A (SignA) outlier test with a Benjamini–Hochberg FDR correction (SignA<0.01 or SignA<0.05) and a one-tailed unpaired Student's *t*-test with permutation-based FDR (*P*<0.01 or *P*<0.05). Gene Ontology (GO) analysis was performed on Tier 1 and Tier 2 significant proteins using the Gene Ontology Resource (Aleksander et al., 2023; Ashburner et al., 2000; Thomas et al., 2021) for biological processes (BP), molecular functions (MF) and cellular component (CC). Enriched terms were filtered using a Fisher's Exact test and corrected using the Bonferroni correction for multiple testing.

### Quantification and statistical analysis

Quantification of immunofluorescent images was performed using Fiji software (Schindelin et al., 2012). For cell line experiments, five images were acquired across two coverslips, with the researcher doing the analysis not being made aware of the cilia marker (ARL13B, aaTub or GT335) staining, and each experiment was repeated two to three times – giving three biological replicates and two technical replicates per experiment. Cilia length was measured manually based on ARL13B^+^ staining. EHBP1 localization was determined manually, based on colocalization of EHBP1 with PCNT or ARL13B. To measure EHBP1 intensity in the primary cilium of cell lines and retinal organoids, regions of interest (ROI) were generated at the basal body or the ciliary compartment and intensity of EHBP1 and either PCNT or ARL13B was measured. Non-ciliary background was subtracted, and results were expressed as a ratio of EHBP1/PCNT or EHBP1/ARL13B. For intensity analysis of EHBP1 siRNA knockdown cells, the raw intensity of EHBP1 in the basal body or connecting cilium was normalized to the average EHBP1 intensity in the respective compartment of untransfected control cells.

For retinal organoid analysis, three to four organoids over two differentiations were analyzed, with at least two images taken per organoid. Area of EHBP1 in the different regions of the organoid was measured by first generating a ROI for the outer segment (based on PNA^+^ expression), ONL and INL. EHBP1 signal was then manually thresholded and total area was measured for each ROI. Finally, the percentage area of EHBP1 within the total area of each ROI was calculated.

Statistical analysis was performed using Prism (GraphPad, San Diego CA, USA) as one-way ANOVA with a post-hoc Tukey's test or unpaired two-tailed *t*-tests. Statistical significance between groups was taken as *P*<0.05, and significance is indicated with asterisks (*P=0.01–0.05; **P=0.01–0.001; ***P=0.001–0.0001; ****P<0.0001). Error bars are standard deviation (s.d.).

## Supplementary Material



10.1242/joces.264091_sup1 Supplementary information

## References

[JCS264091C1] Aleksander, S. A., Balhoff, J., Carbon, S., Michael Cherry, J., Drabkin, H. J., Ebert, D., Feuermann, M., Gaudet, P., Harris, N. L., Hill, D. P. et al. (2023). The Gene Ontology knowledgebase in 2023. *Genetics* 224, iyad031. 10.1093/genetics/iyad031 36866529 PMC10158837

[JCS264091C2] Ashburner, M., Ball, C. A., Blake, J. A., Botstein, D., Butler, H., Cherry, J. M., Davis, A. P., Dolinski, K., Dwight, S. S., Eppig, J. T. et al. (2000). Gene ontology: tool for the unification of biology. *Nat. Genet.* 25, 25. 10.1038/75556 10802651 PMC3037419

[JCS264091C3] Aslanyan, M. G., Doornbos, C., Diwan, G. D., Anvarian, Z., Beyer, T., Junger, K., van Beersum, S. E. C., Russell, R. B., Ueffing, M., Ludwig, A. et al. (2023). A targeted multi-proteomics approach generates a blueprint of the ciliary ubiquitinome. *Front. Cell Dev. Biol.* 11, 1113656. 10.3389/fcell.2023.1113656 36776558 PMC9908615

[JCS264091C4] Babbey, C. M., Bacallao, R. L. and Dunn, K. W. (2010). Rab10 associates with primary cilia and the exocyst complex in renal epithelial cells. *Am. J. Physiol. Ren. Physiol.* 299, F495-F506. 10.1152/ajprenal.00198.2010 PMC2944301 20576682

[JCS264091C5] Bielas, S. L., Silhavy, J. L., Brancati, F., Kisseleva, M. V., Al-Gazali, L., Sztriha, L., Bayoumi, R. A., Zaki, M. S., Abdel-Aleem, A., Rosti, R. O. et al. (2009). Mutations in INPP5E, encoding inositol polyphosphate-5-phosphatase E, link phosphatidyl inositol signaling to the ciliopathies. *Nat. Genet.* 41, 1032-1036. 10.1038/ng.423 19668216 PMC2746682

[JCS264091C6] Borràs, E., de Sousa Dias, M., Hernan, I., Pascual, B., Mañé, B., Gamundi, M., Delás, B. and Carballo, M. (2013). Detection of novel genetic variation in autosomal dominant retinitis pigmentosa. *Clin. Genet.* 84, 441-452. 10.1111/cge.12151 23534816

[JCS264091C7] Chávez, M., Ena, S., Van Sande, J., de Kerchove d'Exaerde, A., Schurmans, S. and Schiffmann, S. N. (2015). Modulation of ciliary phosphoinositide content regulates trafficking and sonic hedgehog signaling output. *Dev. Cell* 34, 338-350. 10.1016/j.devcel.2015.06.016 26190144

[JCS264091C8] Constable, S., Long, A. B., Floyd, K. A., Schurmans, S. and Caspary, T. (2020). The ciliary phosphatidylinositol phosphatase Inpp5e plays positive and negative regulatory roles in Shh signaling. *Development* 147, dev183301. 10.1242/dev.183301 31964774 PMC7033722

[JCS264091C9] Demichev, V., Messner, C. B., Vernardis, S. I., Lilley, K. S. and Ralser, M. (2019). DIA-NN: neural networks and interference correction enable deep proteome coverage in high throughput. *Nat. Methods* 17, 41-44. 10.1038/s41592-019-0638-x 31768060 PMC6949130

[JCS264091C10] Drummond, M. L., Li, M., Tarapore, E., Nguyen, T. T. L., Barouni, B. J., Cruz, S., Tan, K. C., Oro, A. E. and Atwood, S. X. (2018). Actin polymerization controls cilia-mediated signaling. *J. Cell Biol.* 217, 3255-3266. 10.1083/jcb.201703196 29945904 PMC6122990

[JCS264091C11] Dyson, J. M., Conduit, S. E., Feeney, S. J., Hakim, S., DiTommaso, T., Fulcher, A. J., Sriratana, A., Ramm, G., Horan, K. A., Gurung, R. et al. (2017). INPP5E regulates phosphoinositide-dependent cilia transition zone function. *J. Cell Biol.* 216, 247-263. 10.1083/jcb.201511055 27998989 PMC5223597

[JCS264091C12] Faber, S., Letteboer, S. J. F., Junger, K., Butcher, R., Tammana, T. V. S., van Beersum, S. E. C., Ueffing, M., Collin, R. W. J., Liu, Q., Boldt, K. et al. (2023). PDE6D mediates trafficking of prenylated proteins NIM1K and UBL3 to primary cilia. *Cells* 12, 312. 10.3390/cells12020312 36672247 PMC9857354

[JCS264091C13] Fansa, E. K., Kösling, S. K., Zent, E., Wittinghofer, A. and Ismail, S. (2016). PDE6δ-mediated sorting of INPP5E into the cilium is determined by cargo-carrier affinity. *Nat. Commun.* 7, 11366. 10.1038/ncomms11366 27063844 PMC5512577

[JCS264091C14] Garcia-Gonzalo, F. R., Phua, S. C., Roberson, E. C., Garcia, G., Abedin, M., Schurmans, S., Inoue, T. and Reiter, J. F. (2015). Phosphoinositides regulate ciliary protein trafficking to modulate hedgehog signaling. *Dev. Cell* 34, 400-409. 10.1016/j.devcel.2015.08.001 26305592 PMC4557815

[JCS264091C15] Georgiou, M., Chichagova, V., Hilgen, G., Dorgau, B., Sernagor, E., Armstrong, L. and Lako, M. (2020). Room temperature shipment does not affect the biological activity of pluripotent stem cell-derived retinal organoids. *PLoS ONE* 15, e0233860. 10.1371/journal.pone.0233860 32479513 PMC7263587

[JCS264091C16] Gonzalez-Cordero, A., Kruczek, K., Naeem, A., Fernando, M., Kloc, M., Ribeiro, J., Goh, D., Duran, Y., Blackford, S. J. I., Abelleira-Hervas, L. et al. (2017). Recapitulation of human retinal development from human pluripotent stem cells generates transplantable populations of cone photoreceptors. *Stem Cell Rep.* 9, 820-837. 10.1016/j.stemcr.2017.07.022 PMC5599247 28844659

[JCS264091C17] Guilherme, A., Soriano, N. A., Bose, S., Holik, J., Bose, A., Pomerleau, D. P., Furcinitti, P., Leszyk, J., Corvera, S. and Czech, M. P. (2004). EHD2 and the novel EH domain binding protein EHBP1 couple endocytosis to the actin cytoskeleton. *J. Biol. Chem.* 279, 10593-10605. 10.1074/jbc.M307702200 14676205

[JCS264091C18] Gupta, M., Lewis, T. R., Stuck, M. W., Spencer, W. J., Klementieva, N. V., Arshavsky, V. Y. and Pazour, G. J. (2025). Inpp5e is crucial for photoreceptor outer segment maintenance. *J. Cell Sci.* 138, JCS263814. 10.1242/jcs.263814 39871753 PMC11883294

[JCS264091C19] Hasenpusch-Theil, K., Laclef, C., Colligan, M., Fitzgerald, E., Howe, K., Carroll, E., Abrams, S. R., Reiter, J. F., Schneider-Maunoury, S. and Theil, T. (2020). A transient role of the ciliary gene inpp5e in controlling direct versus indirect neurogenesis in cortical development. *eLife* 9, e58162. 10.7554/eLife.58162 32840212 PMC7481005

[JCS264091C20] Humbert, M. C., Weihbrecht, K., Searby, C. C., Li, Y., Pope, R. M., Sheffield, V. C. and Seo, S. (2012). ARL13B, PDE6D, and CEP164 form a functional network for INPP5E ciliary targeting. *Proc. Natl. Acad. Sci. USA* 109, 19691-19696. 10.1073/pnas.1210916109 23150559 PMC3511769

[JCS264091C21] Huttlin, E. L., Bruckner, R. J., Navarrete-Perea, J., Cannon, J. R., Baltier, K., Gebreab, F., Gygi, M. P., Thornock, A., Zarraga, G., Tam, S. et al. (2021). Dual proteome-scale networks reveal cell-specific remodeling of the human interactome. *Cell* 184, 3022-3040.e28. 10.1016/j.cell.2021.04.011 33961781 PMC8165030

[JCS264091C22] Iwano, T., Sobajima, T., Takeda, S., Harada, A. and Yoshimura, S. (2023). The Rab GTPase-binding protein EHBP1L1 and its interactors CD2AP/CIN85 negatively regulate the length of primary cilia via actin remodeling. *J. Biol. Chem.* 299, 102985. 10.1016/j.jbc.2023.102985 36754282 PMC9986712

[JCS264091C23] Jacoby, M., Cox, J. J., Gayral, S., Hampshire, D. J., Ayub, M., Blockmans, M., Pernot, E., Kisseleva, M. V., Compère, P., Schiffmann, S. N. et al. (2009). INPP5E mutations cause primary cilium signaling defects, ciliary instability and ciliopathies in human and mouse. *Nat. Genet.* 41, 1027-1031. 10.1038/ng.427 19668215

[JCS264091C24] Johnstone, M., Vasistha, N. A., Barbu, M. C., Dando, O., Burr, K., Christopher, E., Glen, S., Robert, C., Fetit, R., Macleod, K. G. et al. (2018). Reversal of proliferation deficits caused by chromosome 16p13.11 microduplication through targeting NFκB signaling: an integrated study of patient-derived neuronal precursor cells, cerebral organoids and in vivo brain imaging. *Mol. Psychiatry* 24, 294-311. 10.1038/s41380-018-0292-1 30401811 PMC6344377

[JCS264091C25] Kim, J., Jo, H., Hong, H., Kim, M. H., Kim, J. M., Lee, J. K., Heo, W. D. and Kim, J. (2015). Actin remodelling factors control ciliogenesis by regulating YAP/TAZ activity and vesicle trafficking. *Nat. Commun.* 6, 6781. 10.1038/ncomms7781 25849865

[JCS264091C26] Knödler, A., Feng, S., Zhang, J., Zhang, X., Das, A., Peränen, J. and Guo, W. (2010). Coordination of Rab8 and Rab11 in primary ciliogenesis. *Proc. Natl. Acad. Sci. USA* 107, 6346-6351. 10.1073/pnas.1002401107 20308558 PMC2851980

[JCS264091C27] Kuwahara, A., Ozone, C., Nakano, T., Saito, K., Eiraku, M. and Sasai, Y. (2015). Generation of a ciliary margin-like stem cell niche from self-organizing human retinal tissue. *Nat. Commun.* 6, 6286. 10.1038/ncomms7286 25695148

[JCS264091C28] Li, Z., Schulze, R. J., Weller, S. G., Krueger, E. W., Schott, M. B., Zhang, X., Casey, C. A., Liu, J., Stöckli, J., James, D. E. et al. (2016). A novel Rab10-EHBP1-EHD2 complex essential for the autophagic engulfment of lipid droplets. *Sci. Adv.* 2, e1601470. 10.1126/sciadv.1601470 28028537 PMC5161429

[JCS264091C29] Liu, S., Wei, J., Zhong, L., Hai, S., Song, S., Xie, C., Huang, Z., Cheng, Z., Zhang, J., Du, A. et al. (2025). RAB-10 cooperates with EHBP-1 to capture vesicular carriers during post-Golgi exocytic trafficking. *J. Cell Biol.* 224, e202410003. 10.1083/jcb.202410003 39982707 PMC11844438

[JCS264091C30] Mattern, S., Hollfoth, V., Bag, E., Ali, A., Riemenschneider, P., Jarboui, M. A., Boldt, K., Sulyok, M., Dickemann, A., Luibrand, J. et al. (2025). An AI-assisted morphoproteomic approach is a supportive tool in esophagitis-related precision medicine. *EMBO Mol. Med.* 17, 441. 10.1038/s44321-025-00194-7 39901020 PMC11903792

[JCS264091C31] Merk, D. J., Tsiami, F., Hirsch, S., Walter, B., Haeusser, L. A., Maile, J. D., Stahl, A., Jarboui, M. A., Lechado-Terradas, A., Klose, F. et al. (2024). Functional screening reveals genetic dependencies and diverging cell cycle control in atypical teratoid rhabdoid tumors. *Genome Biol.* 25, 301. 10.1186/s13059-024-03438-w 39617889 PMC11610224

[JCS264091C32] Mill, P., Christensen, S. T. and Pedersen, L. B. (2023). Primary cilia as dynamic and diverse signalling hubs in development and disease. *Nat. Rev. Genet.* 24, 421-441. 10.1038/s41576-023-00587-9 37072495 PMC7615029

[JCS264091C33] Nozaki, S., Katoh, Y., Terada, M., Michisaka, S., Funabashi, T., Takahashi, S., Kontani, K. and Nakayama, K. (2017). Regulation of ciliary retrograde protein trafficking by the Joubert syndrome proteins ARL13B and INPP5E. *J. Cell Sci.* 130, 563-576. 10.1242/jcs.197004 27927754

[JCS264091C34] Phua, S. C., Chiba, S., Suzuki, M., Su, E., Roberson, E. C., Pusapati, G. V., Setou, M., Rohatgi, R., Reiter, J. F., Ikegami, K. et al. (2017). Dynamic remodeling of membrane composition drives cell cycle through primary cilia excision. *Cell* 168, 264-279.e15. 10.1016/j.cell.2016.12.032 28086093 PMC5660509

[JCS264091C35] Qiu, H., Fujisawa, S., Nozaki, S., Katoh, Y. and Nakayama, K. (2021). Interaction of INPP5E with ARL13B is essential for its ciliary membrane retention but dispensable for its ciliary entry. *Biol. Open* 10, bio057653. 10.1242/bio.057653 33372066 PMC7860134

[JCS264091C36] Rai, A., Oprisko, A., Campos, J., Fu, Y., Friese, T., Itzen, A., Goody, R. S., Gazdag, E. M. and Müller, M. P. (2016). Bmerb domains are bivalent rab8 family effectors evolved by gene duplication. *eLife* 5, e18675. 10.7554/eLife.18675 27552051 PMC5026484

[JCS264091C37] Rai, A., Bleimling, N., Vetter, I. R. and Goody, R. S. (2020). The mechanism of activation of the actin binding protein EHBP1 by Rab8 family members. *Nat. Commun.* 11, 4187. 10.1038/s41467-020-17792-3 32826901 PMC7442826

[JCS264091C38] Rao, K. N., Zhang, W., Li, L., Ronquillo, C., Baehr, W. and Khanna, H. (2016). Ciliopathy-associated protein CEP290 modifies the severity of retinal degeneration due to loss of RPGR. *Hum. Mol. Genet.* 25, 2005-2012. 10.1093/hmg/ddw075 26936822 PMC5062589

[JCS264091C39] Sangermano, R., Deitch, I., Peter, V. G., Ba-Abbad, R., Place, E. M., Zampaglione, E., Wagner, N. E., Fulton, A. B., Coutinho-Santos, L., Rosin, B. et al. (2021). Broadening INPP5E phenotypic spectrum: detection of rare variants in syndromic and non-syndromic IRD. *NPJ Genomic Med.* 6, 53. 10.1038/s41525-021-00214-8 PMC8242099 34188062

[JCS264091C40] Schembs, L., Willems, A., Hasenpusch-Theil, K., Cooper, J. D., Whiting, K., Burr, K., Bøstrand, S. M. K., Selvaraj, B. T., Chandran, S. and Theil, T. (2022). The ciliary gene INPP5E confers dorsal telencephalic identity to human cortical organoids by negatively regulating Sonic hedgehog signaling. *Cell Rep.* 39, 110811. 10.1016/j.celrep.2022.110811 35584663 PMC9620745

[JCS264091C41] Schindelin, J., Arganda-Carreras, I., Frise, E., Kaynig, V., Longair, M., Pietzsch, T., Preibisch, S., Rueden, C., Saalfeld, S., Schmid, B. et al. (2012). Fiji: an open-source platform for biological-image analysis. *Nat. Methods* 9, 676-682. 10.1038/nmeth.2019 22743772 PMC3855844

[JCS264091C42] Selvaraj, B. T., Livesey, M. R., Zhao, C., Gregory, J. M., James, O. T., Cleary, E. M., Chouhan, A. K., Gane, A. B., Perkins, E. M., Dando, O. et al. (2018). C9ORF72 repeat expansion causes vulnerability of motor neurons to Ca2+-permeable AMPA receptor-mediated excitotoxicity. *Nat. Commun.* 9, 347. 10.1038/s41467-017-02729-0 29367641 PMC5783946

[JCS264091C43] Sharif, A. S., Gerstner, C. D., Cady, M. A., Arshavsky, V. Y., Mitchell, C., Ying, G., Frederick, J. M. and Baehr, W. (2021). Deletion of the phosphatase INPP5E in the murine retina impairs photoreceptor axoneme formation and prevents disc morphogenesis. *J. Biol. Chem.* 296, 100529. 10.1016/j.jbc.2021.100529 33711342 PMC8047226

[JCS264091C44] Shetty, M., Ramdas, N., Sahni, S., Mullapudi, N. and Hegde, S. (2017). A homozygous missense variant in INPP5E associated with Joubert syndrome and related disorders. *Mol. Syndromol.* 8, 313-317. 10.1159/000479673 29230161 PMC5701266

[JCS264091C45] Smith, C. E. L., Lake, A. V. R. and Johnson, C. A. (2020). Primary cilia, ciliogenesis and the actin cytoskeleton: a little less resorption, a little more actin please. *Front. Cell Dev. Biol.* 8, 622822. 10.3389/fcell.2020.622822 33392209 PMC7773788

[JCS264091C46] Thomas, S., Wright, K. J., Le Corre, S., Micalizzi, A., Romani, M., Abhyankar, A., Saada, J., Perrault, I., Amiel, J. et al. (2014). A homozygous PDE6D mutation in Joubert syndrome impairs targeting of farnesylated INPP5E protein to the primary cilium. *Hum. Mutat.* 35, 137-146. 10.1002/humu.22470 24166846 PMC3946372

[JCS264091C47] Thomas, P. D., Ebert, D., Muruganujan, A., Mushayahama, T., Albou, L. P. and Mi, H. (2021). PANTHER: making genome-scale phylogenetics accessible to all. *Protein Sci.* 31, 8. 10.1002/pro.4218 34717010 PMC8740835

[JCS264091C48] Tyanova, S., Temu, T., Sinitcyn, P., Carlson, A., Hein, M. Y., Geiger, T., Mann, M. and Cox, J. (2016). The Perseus computational platform for comprehensive analysis of (prote)omics data. *Nat. Methods* 13, 731-740. 10.1038/nmeth.3901 27348712

[JCS264091C49] Vasistha, N. A., Johnstone, M., Barton, S. K., Mayerl, S. E., Thangaraj Selvaraj, B., Thomson, P. A., Dando, O., Grünewald, E., Alloza, C., Bastin, M. E. et al. (2019). Familial t(1;11) translocation is associated with disruption of white matter structural integrity and oligodendrocyte–myelin dysfunction. *Mol. Psychiatry* 24, 1641-1654. 10.1038/s41380-019-0505-2 31481758 PMC6814440

[JCS264091C50] Vössing, C., Atigbire, P., Eilers, J., Markus, F., Stieger, K., Song, F. and Neidhardt, J. (2021). The major ciliary isoforms of rpgr build different interaction complexes with inpp5e and rpgrip1l. *Int. J. Mol. Sci.* 22, 3583. 10.3390/ijms22073583 33808286 PMC8037643

[JCS264091C51] Wang, P., Liu, H., Wang, Y., Liu, O., Zhang, J., Gleason, A., Yang, Z., Wang, H., Shi, A. and Grant, B. D. (2016). RAB-10 promotes EHBP-1 bridging of filamentous actin and tubular recycling endosomes. *PLoS Genet.* 12, e1006093. 10.1371/journal.pgen.1006093 27272733 PMC4894640

[JCS264091C52] Whiting, K. R., Haer-Wigman, L., Florijn, R. J., van Beek, R., Oud, M. M., Plomp, A. S., Boon, C. J. F., Kroes, H. Y. and Roepman, R. (2024). Utilization of automated cilia analysis to characterize novel INPP5E variants in patients with non-syndromic retinitis pigmentosa. *Eur. J. Hum. Genet.* 32, 1412-1418. 10.1038/s41431-024-01627-6 38806661 PMC11576733

[JCS264091C53] Whiting, K. R., Aslanyan, M. G., van Summeren, L., Beyer, T., Dahlke, K., Theil, T., Boldt, K. and Roepman, R. (2026). Loss of INPP5E affects photoreceptor outer segment membrane biogenesis in iPSC-derived human retinal organoids. *J. Cell Sci.* 139, jcs264431. 10.1242/jcs.264431 42374981 PMC13450897

[JCS264091C54] Zhang, R., Tang, J., Li, T., Zhou, J. and Pan, W. (2022). INPP5E and coordination of signaling networks in Cilia. *Front. Mol. Biosci.* 9, 885592. 10.3389/fmolb.2022.885592 35463949 PMC9019342

