# Supplementary material for: The EH-binding protein EHBP1 operates in a ciliary functional module affected by INPP5E dysfunction
Source: J Cell Sci. 2026 Jul 21;139(14):jcs264091. doi: 10.1242/jcs.264091 (PMC13450894; doi:10.1242/jcs.264091)
Supplement: Supplementary file 1 [file joces-139-264091-s1.pdf]

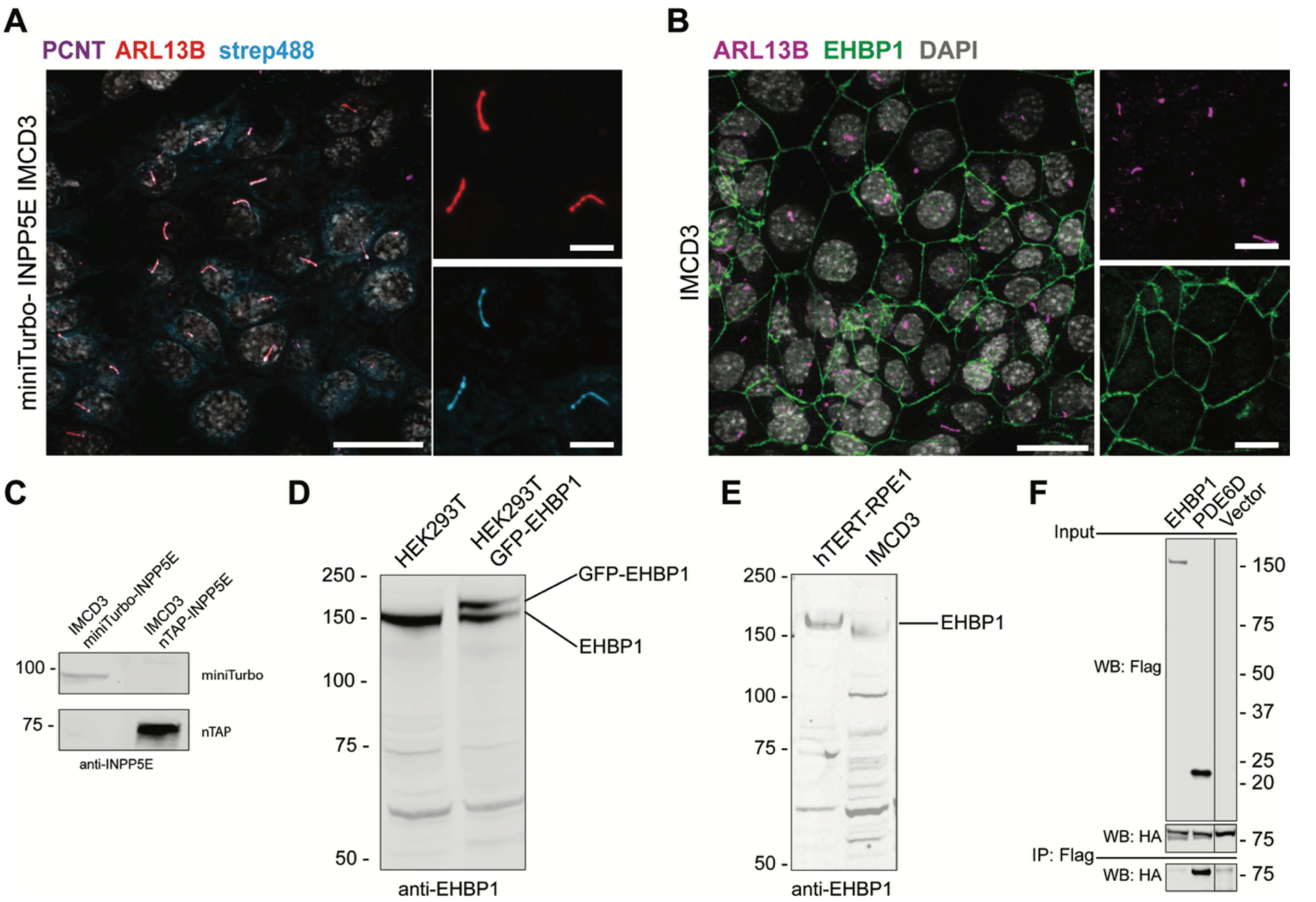

Fig. S1. Validation of miniTurbo-INPP5E Flp-In cell lines and EHBP1 antibody specificity. (A) Immunofluorescence of miniTurbo-INPP5E Flp-In IMCD3 cells after 24h biotin incubation. Cells are stained against pericentrin (PCNT, magenta), ARL13B (red), and biotin (strep488, blue). Scale bars: 25 µm and 5 µm. (B) Immunofluorescence of EHBP1 in mouse IMCD3 cells shows no ciliary localization. Stained against ARL13B (magenta), EHBP1 (green), and DAPI (grey). Scale bars: 20 µm and 5 µm. (C) Western blot analysis of miniTurbo-INPP5E Flp-In cells and a positive control nTAP-INPP5E, stained against INPP5E. (D) Western blot analysis to confirm EHBP1 antibody specificity. Control HEK293T cells and cells transfected with human GFP-EHBP1 plasmid DNA were lysed, and bands for endogenous EHBP1 (150kDa) and GFP-EHBP1 (176kDa) are visualized using antibodies against EHBP1. (E) Western blot analysis of endogenous EHBP1 expression in hTERT-RPE1 and IMCD3 cells using antibodies against EHBP1. (F) 3xHA-INPP5E was coexpressed with 3xFlag-EHBP1, 3xFlag-PDE6D, and a 3xFlag-Empty Vector (Vector) in HEK293T cells. Immunoprecipitation was performed with anti-FLAG beads, followed by western blot analysis with the antibodies indicated.

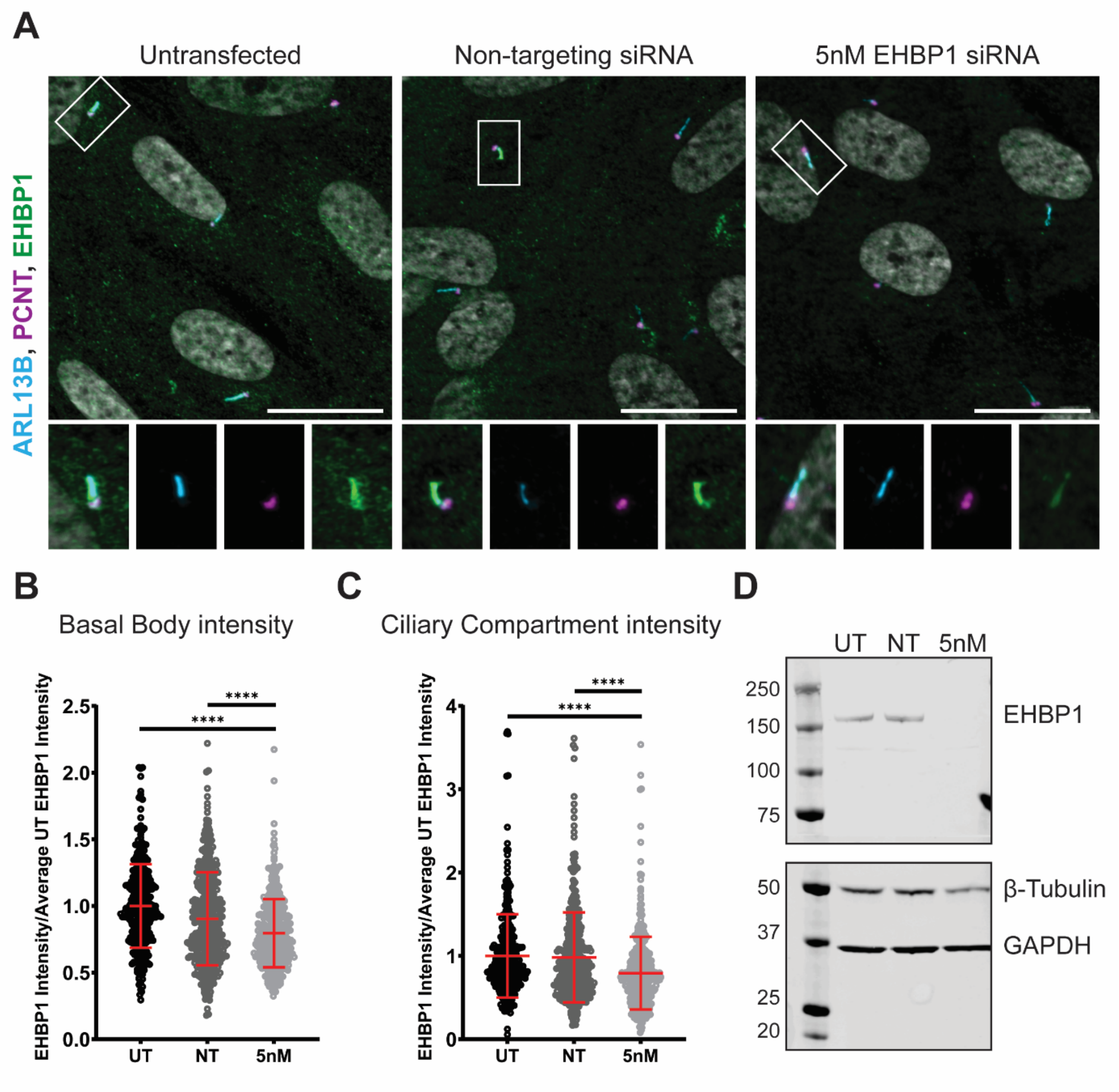

Fig. S2. siRNA knock-down of EHBP1 confirms antibody specificity. (A) Representative immunofluorescent images of untransfected (UT) control, non-targeting siRNA control (NT), and 5nM EHBP1 siRNA RPE cells, stained against ARL13B (blue), pericentrin (PCNT, magenta), EHBP1 (green), and DAPI (grey). Scale bar: 20µm. (B-C) Quantification of EHBP1 intensity, measured as the raw EHBP1 mean intensity fluorescent normalized to the average UT intensity in the (B) basal body or the (C) ciliary compartment (n=2 experiments). Statistical significance was determined by unpaired two-tailed t-test (****, $p<0.0001$). (D) Western blot analysis confirms knock-down of EHBP1, visualized with anti-EHBP1 antibodies (150 kDa) in cells transfected with 5nM EHBP1 siRNA compared to UT and NT control cells. β-tubulin (50kDa) and GAPDH (36 kDa) were used as loading controls.

Journal of Cell Science • Supplementary information

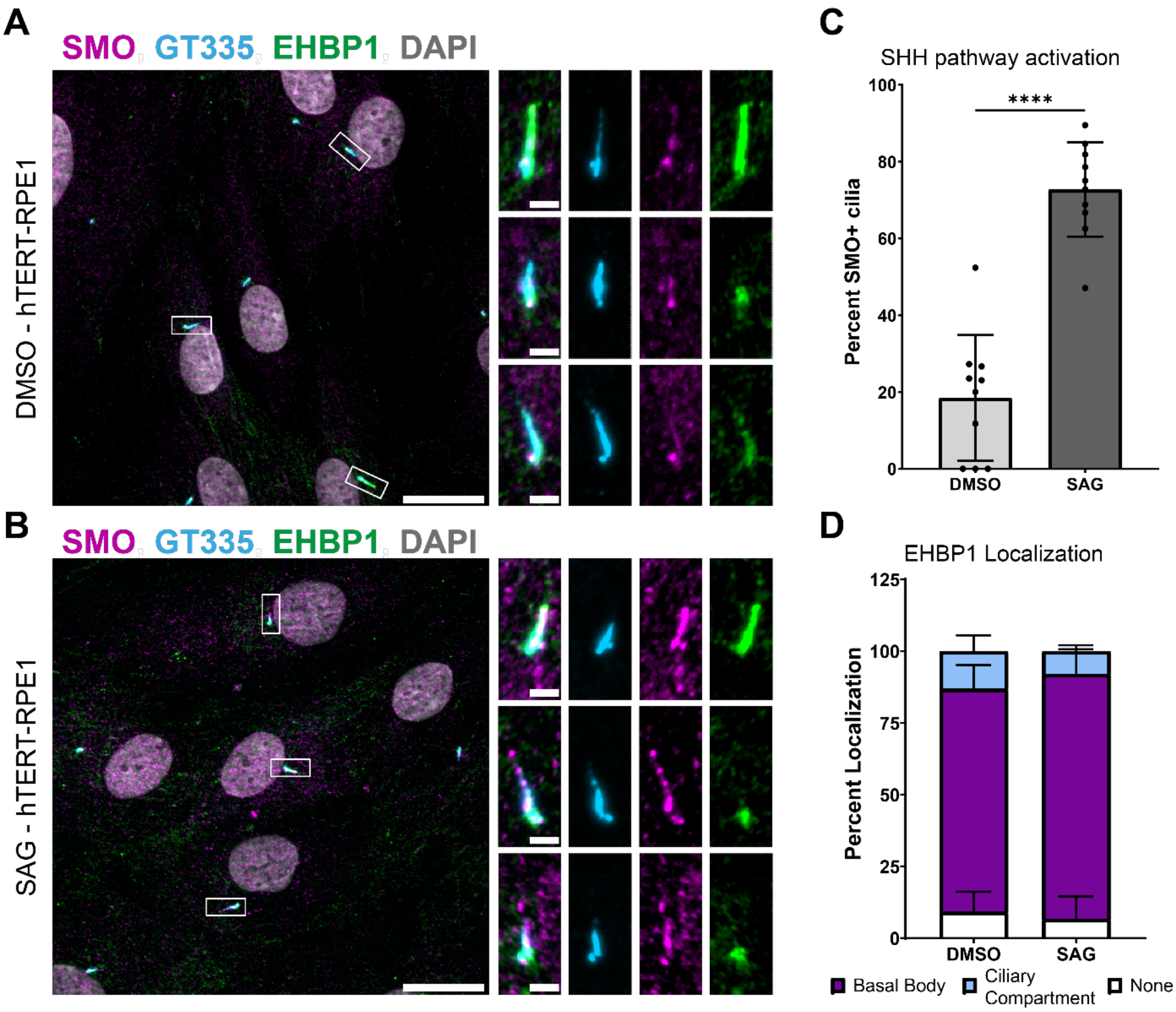

Fig. S3. SHH pathway activation does not alter EHBP1 ciliary localization

(A,B) Representative immunofluorescence of fibroblasts treated by vehicle (DMSO, A) or SHH agonist SAG (B). Images are stained against GT335 (blue), Smoothened (SMO, magenta), EHBP1 (green), and DAPI (grey). Scale bars: 20 µm and 5 µm. (C) Quantification of SHH pathway stimulation as the percent of SMO positive cilia. (D) Quantification of EHBP1 localization in the cilium (n=2 experiments). Statistical significance was determined by unpaired two-tailed t-test (****, p<0.0001).

Journal of Cell Science • Supplementary information

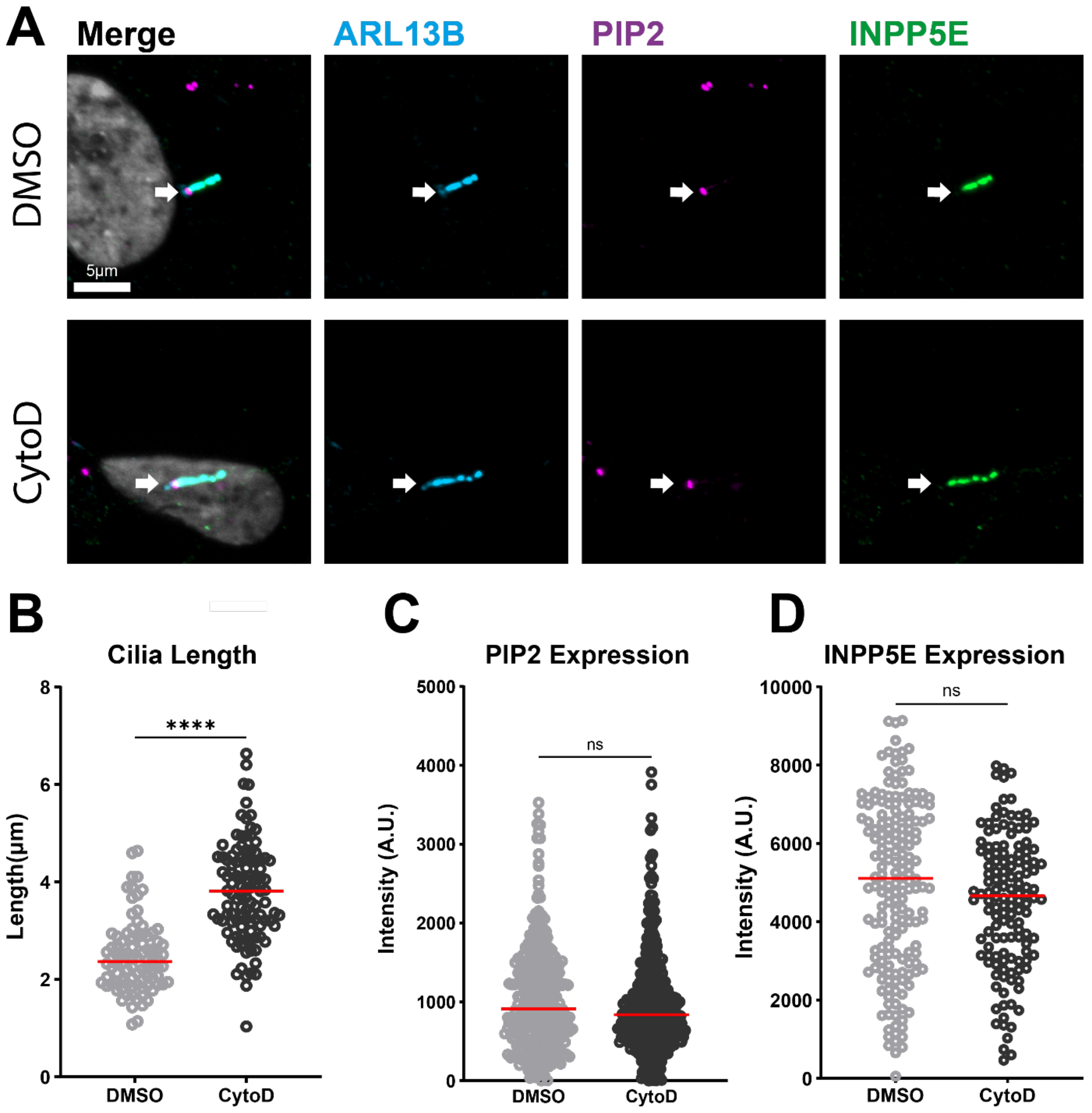

Fig. S4. Disruption of actin does not affect INPP5E or PIP2 expression.
(A) Immunofluorescence of RPE cells treated with DMSO vehicle or 10 µM CytoD for 1 hr, stained against ARL13B (blue), PIP2 (magenta) and INPP5E (green). Scale bars: 5 µm. (B-D) Quantification of cilia length (B), PIP2 expression at the ciliary base (C), and INPP5E expression in the ciliary compartment (D) in RPE cells after 1 hr treatment with either DMSO or 10 µM CytoD (n=2 experiments). Significance was determined with an unpaired two-tail t-test (****, $p<0.0001$).

Journal of Cell Science • Supplementary information

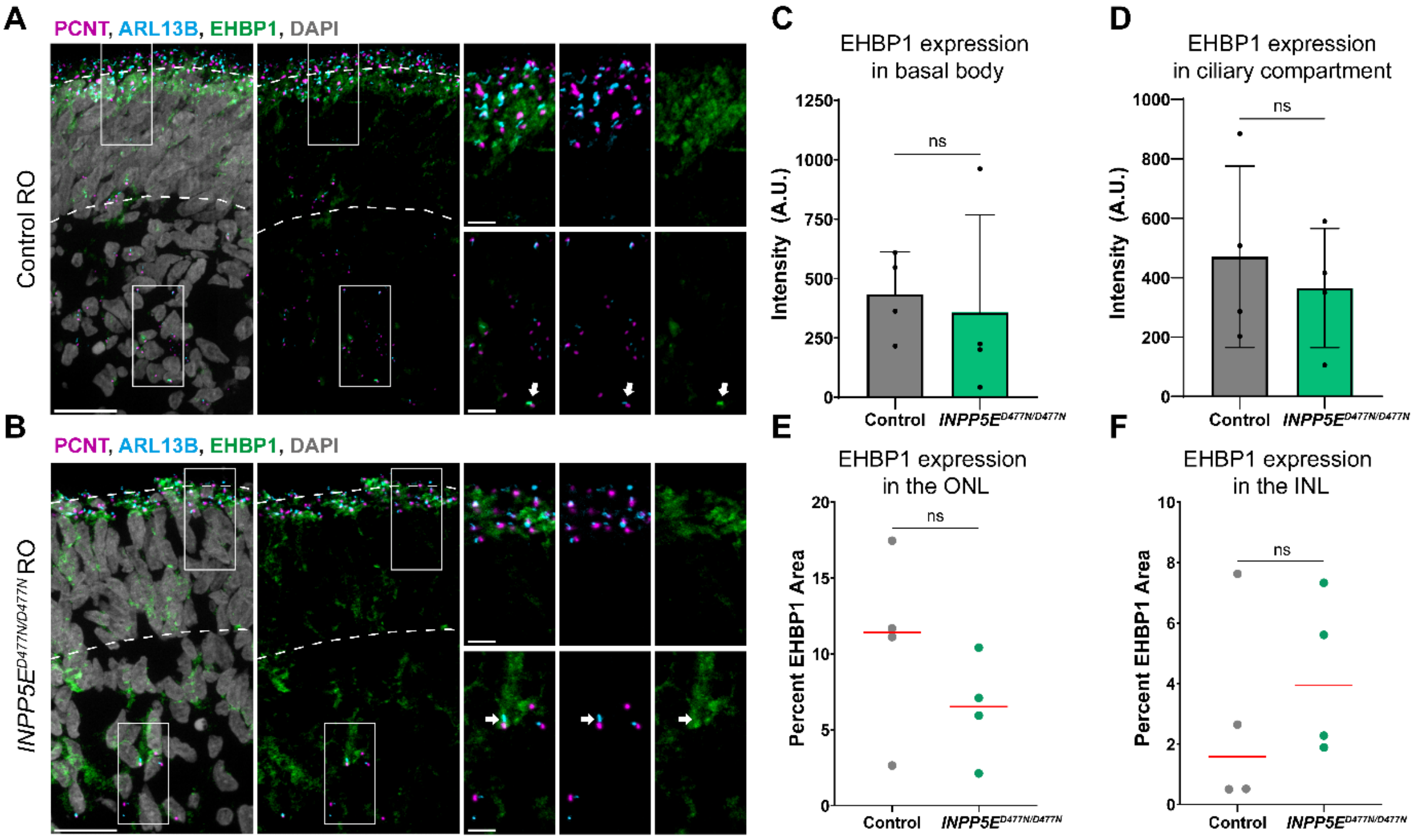

Fig. S5. EHBP1 is not altered in *INPP5E*$^{D477N/D477N}$ organoids at d35.

(A,B) Representative images of control (A) and *INPP5*$^{D477N/D477N}$ (B) retinal organoids at d35, stained against pericentrin (PCNT, magenta), ARL13B (blue), EHBP1 (green), and DAPI (grey). Dotted lines separate the ONL and INL. Scale bars: 20µm and 2µm. (C-F) Quantification of EHBP1 expression as the normalized intensity of EHBP1 in the basal body (C) and ciliary compartment (D), and the percent EHBP1 area in the outer nuclear layer (ONL) (E) and inner nuclear layer (F) (n=3 organoids). Statistical analysis performed as an unpaired two-tail t-test.

Journal of Cell Science • Supplementary information

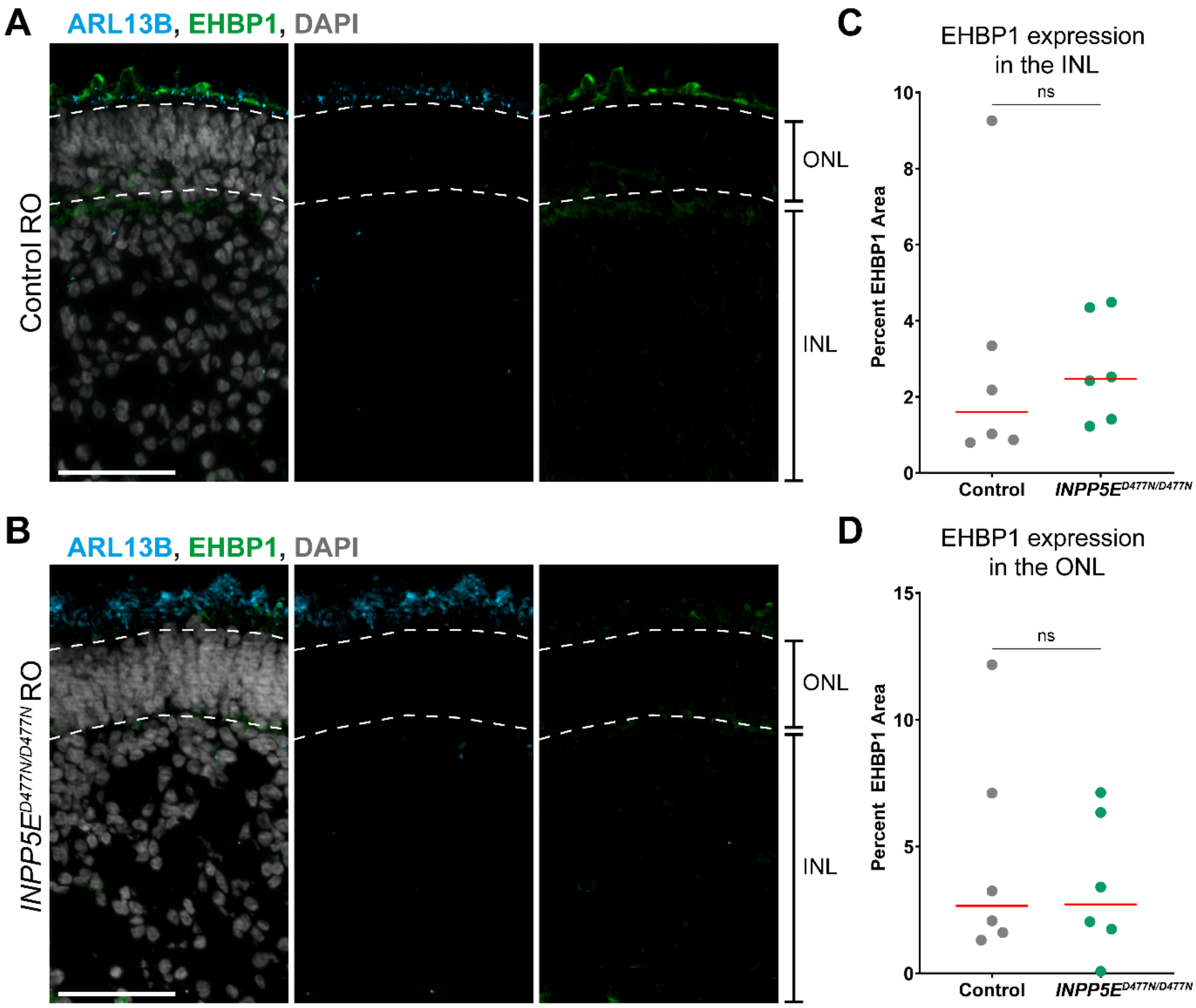

Fig. S6. Localization of EHBP1 in deep RO layers is not affected by the loss of INPP5E at d230. (A,B) Immunofluorescence of d230 control (A) and *INPP5E*$^{D477N/D477N}$ (B) ROs, stained against ARL13B (blue), EHBP1 (green), and DAPI (grey). Dotted lines denote the ONL and INL; scale bars: 20µm. (C-D) Quantification of EHBP1 expression as the percent EHBP1 positive area in the INL (C) and ONL (D) (n= 6 organoids). Statistical analysis performed as an unpaired two-tail t-test.

Journal of Cell Science • Supplementary information

From Fig. S1C

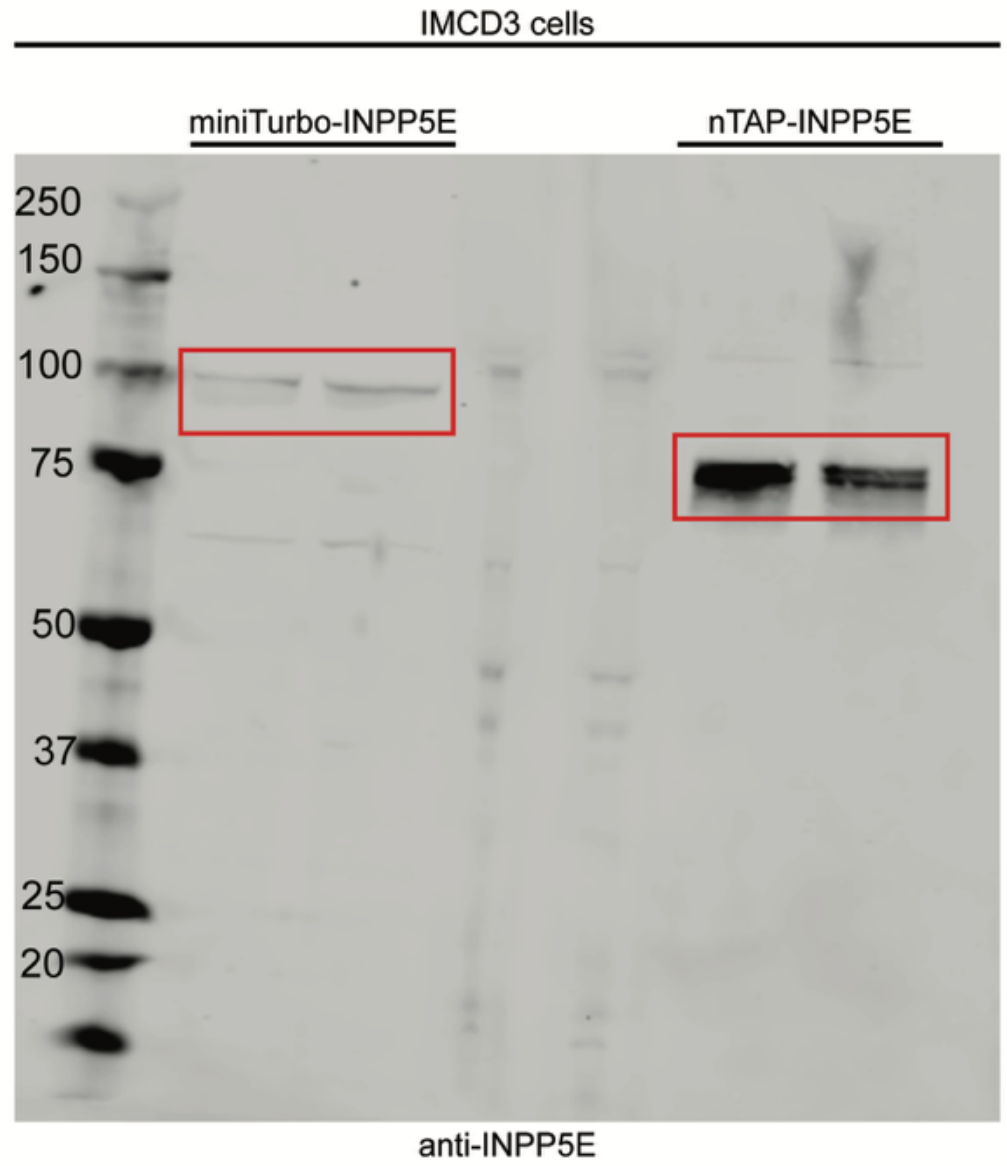

From Fig. S1D

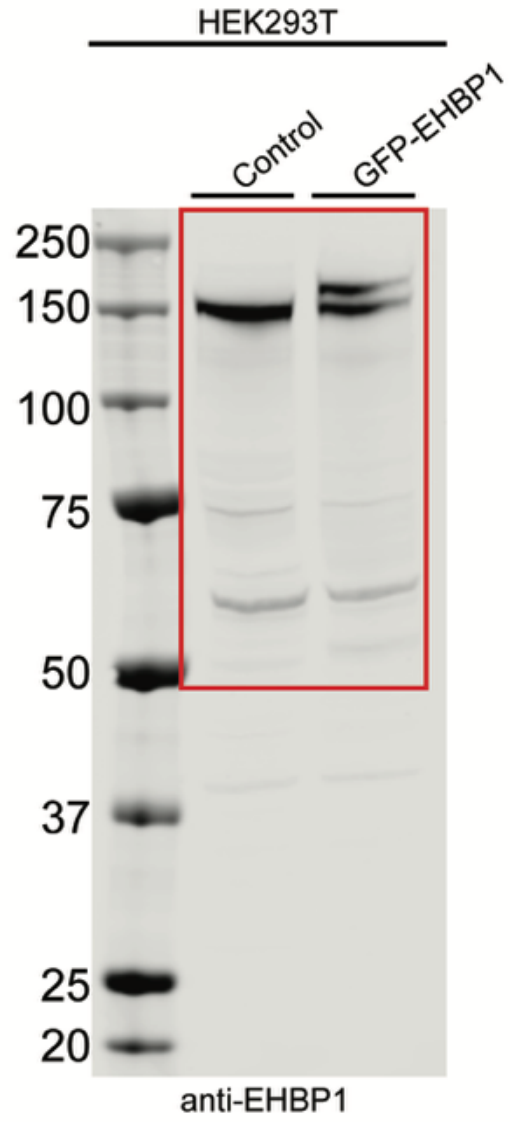

From Fig. S1E

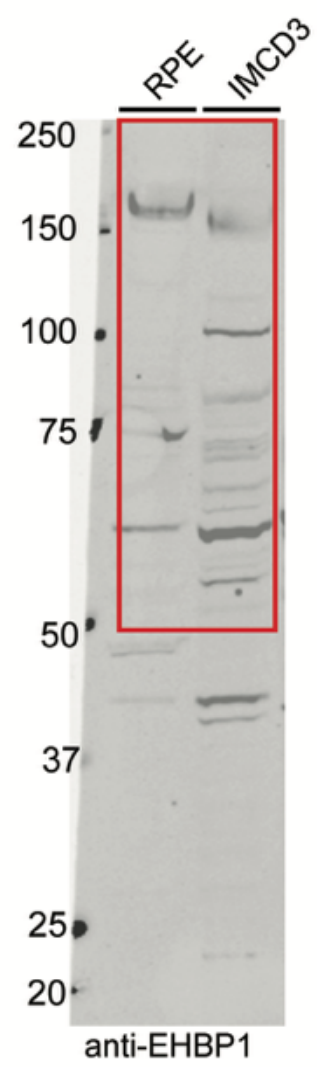

From Fig. S1F

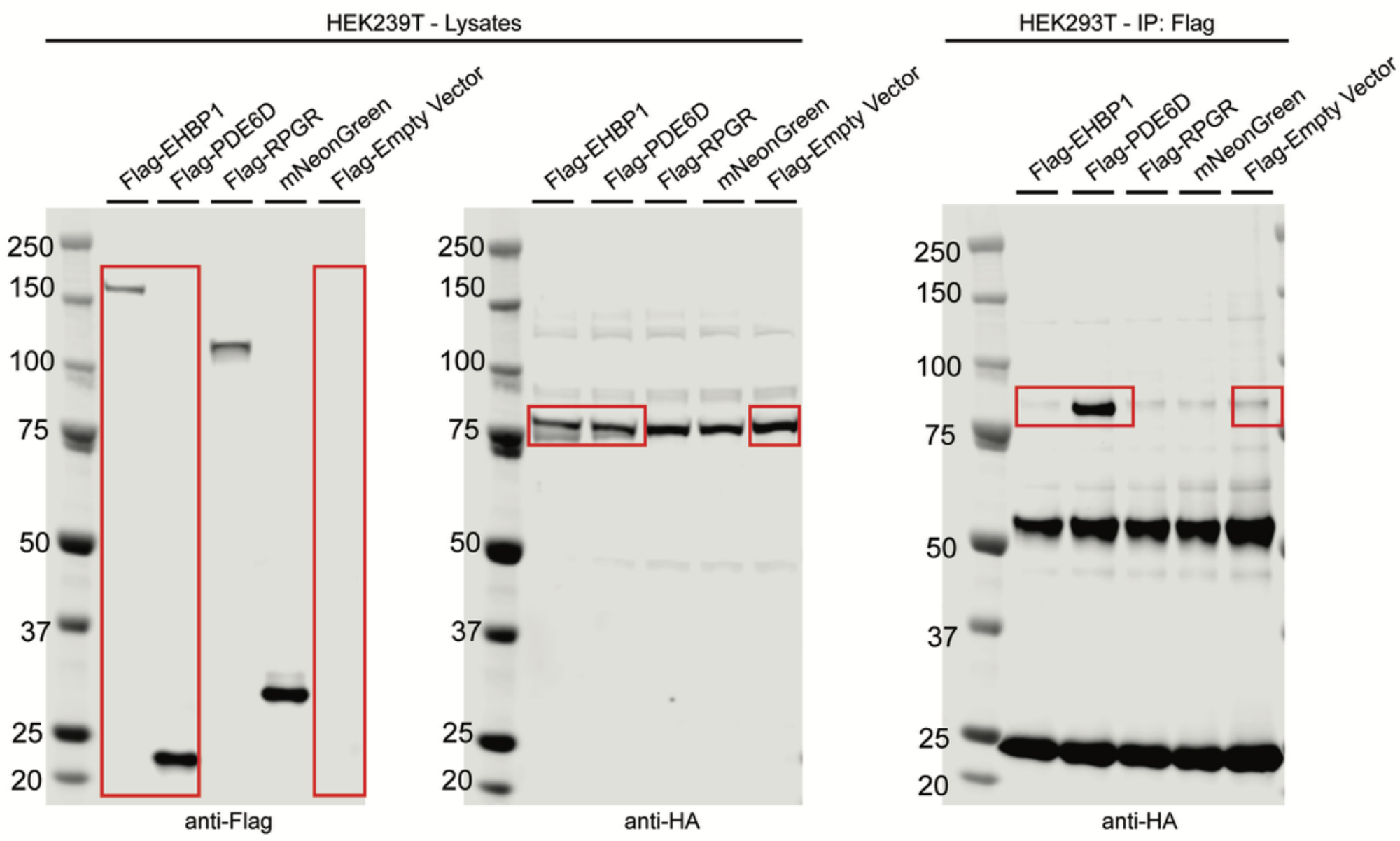

From Fig. S2D

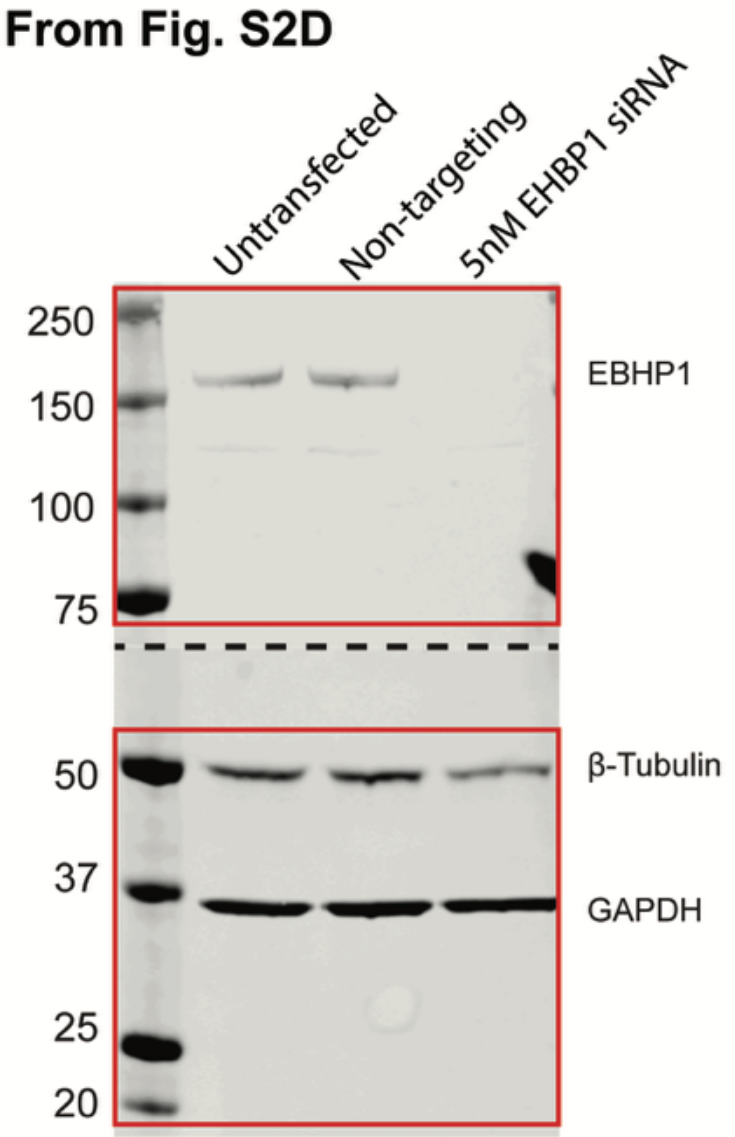

Journal of Cell Science • Supplementary information

Fig. S7. Western blot transparency – Full, uncropped membranes from western blot analysis used in Figs 1 and 2. Red boxes where each membrane was cropped for visualization in the figures. The dotted line in Fig. S2D indicates where the membrane was manually cut for optimization of loading control (β-Tubulin and GAPDH) expression.

Journal of Cell Science • Supplementary information

Table S1. Antibodies used for immunofluorescence and western blot

Antibody	Dilution	Source	Catalog number
Anti-Arl13B (Monoclonal mouse, IgG2a)	1:300	NeuroMab	75-287
Anti-ARL13B (Rabbit)	1:500	Proteintech	17711
Anti-ARL13B (Guinea pig)	1:1000	Proteintech	30332
Anti-EHBP1 (Rabbit)	1:500 (IF, WB)	Proteintech	17637
Anti-INPP5E (Rabbit)	1:500 (IF); 1:1000 (WB)	Proteintech	17797
Anti-Pericentrin (Monoclonal mouse, IgG1)	1:1,000	Abcam	Ab28144
Anti-PNA (568-conjugated)	1:100	Thermo Fisher Scientific	L32458
Anti-aaTub (Monoclonal Mouse, IgG2b)	1:1,000	Sigma Aldrich	T6793
Anti-Phalloidin (568-conjugated)	1:500	Invitrogen	A12380
Anti-streptavidin (488-conjugated)	1:1,000	Thermo Fisher Scientific	S32354
Anti-GT335 (monoclonal mouse, IgG1)	1:500	AdipoGen Life Sciences	AG-20B-0020-C100
Anti-SMO (monoclonal mouse, IgG2a)	1:500	Santa Cruz Biotechnology	Sc-166685
Anti-PIP2 (monoclonal mouse, IgM)	1:500	Santa Cruz Biotechnology	Sc-53412
Anti-β tubulin (Rabbit)	1:1000	Abcam	AB15568
Anti-GAPDH (Mouse)	1:1000	Invitrogen	11335232
Anti-Mouse IgG1 (Goat)	1:500	Invitrogen	A21121
Anti-Mouse IgG2a (Goat)	1:500	Thermo Fisher Scientific	A21241
Anti-Mouse IgG2b (Goat)	1:500	Thermo Fisher Scientific	A21144
Anti-Mouse (Goat)	1:500	Life Technologies	A11031
Anti-Rabbit (Goat)	1:500	Molecular Probes	A11008
Anti-Rabbit (Goat)	1:500	Molecular Probes	A110011
Anti-Guinea pig (Goat)	1:500	Molecular Probes	A21450
Anti-Mouse (Goat, 800)	1:10,000	Li-COR	LI 926-32211
Anti-Rabbit (Goat, 680)	1,10,000	Molecular Probes	A21076
Anti-Rabbit (Goat, 800)	1,10,000	Li-COR	LI 926-32211
DAPI	1:100	Thermo Fisher Scientific	D1306

Table S2. Significant proteins identified through proximity proteomics of INPP5E.

Protein	Protein Description	INPP5E/Empty Vector	Student's T-test p-value	Significance A	Tier
INPP5E	Phosphatidylinositol polyphosphate 5-phosphatase type IV	252,503	3.94E-18	5.79E-95	1
EHBP1	EH domain-binding protein 1	20,177	0,00399415	1.63E-59	1
SLC25A46	Mitochondrial outer membrane protein SLC25A46	199,727	0,00227839	2.98E-58	1
KLRG2	Killer cell lectin-like receptor subfamily G member 2	195,778	5.23E-17	7.53E-56	1
FAM135A	Protein FAM135A	192,738	6.88E-16	4.95E-54	1
GLS	Glutaminase kidney isoform, mitochondrial	192,034	0,00299626	1.29E-53	1
ARL13B	ADP-ribosylation factor-like protein 13B	19,189	8.52E-15	1.57E-53	1
SYNE2	Nesprin-2	189,715	0,00174655	2.97E-52	1
ANKS6	Ankyrin repeat and SAM domain-containing protein 6	186,678	1.99E-15	1.71E-51	1
ESCO2	N-acetyltransferase ESCO2	186,771	0,00377208	1.51E-50	1
PALM	Paralemmin-1	186,285	0,00783243	2.87E-50	1
POLR2E	DNA-directed RNA polymerases I, II, and III subunit RPABC1	185,578	0,00485974	7.29E-50	1
LCA5	Lebercilin	185,477	8.96E-16	8.32E-50	1
NEK8	Serine/threonine-protein kinase Nek8	184,629	8.85E-15	2.53E-49	1
IFT172	Intraflagellar transport protein 172 homolog	184,067	2.09E-14	5.28E-49	1
ATG2B	Autophagy-related protein 2 homolog B	184,012	0,00301074	5.67E-49	1
CYTH1	Cytohesin-1	183,548	0,000269533	1.04E-48	1
RPGRIP1L	Protein fantom	182,928	8.71E-14	2.32E-48	1
PAN2	PAN2-PAN3 deadenylation complex catalytic subunit Pan2	182,899	0,000268819	2.41E-48	1
MCL1	Induced myeloid leukemia cell differentiation protein Mcl-1 homolog	182,877	0,00348722	2.48E-48	1
CRTC1	CREB-regulated transcription coactivator 1	182,387	0,00295041	4.68E-48	1
NIPSNAP2	Protein NipSnap homolog 2	181,846	0,00447512	9.40E-48	1
ATP5PF	ATP synthase-coupling factor 6, mitochondrial	180,642	0,000346238	4.41E-47	1
PTGR3	Prostaglandin reductase-3	179,583	0,00359782	1.71E-46	1
PALD1	Paladin	179,446	0,000173417	2.03E-46	1
GCDH	Glutaryl-CoA dehydrogenase, mitochondrial	179,412	7.89E-15	2.12E-46	1
NDUFS2	NADH dehydrogenase [ubiquinone] iron-sulfur protein 2, mitochondrial	179,259	0,000136636	2.57E-46	1
ICA1	Islet cell autoantigen 1	178,408	9.76E-17	7.58E-46	1
PRADC1	Protease-associated domain-containing protein 1	178,226	0,000299123	9.53E-46	1
NIBAN2	Protein Niban 2	178,204	8.01E-12	9.79E-46	1
GLUD1	Glutamate dehydrogenase 1, mitochondrial	178,023	0,00497558	1.23E-45	1
IARS2	Isoleucine--tRNA ligase, mitochondrial	177,999	0,000143333	1.27E-45	1
DENND4C	DENN domain-containing protein 4C	177,939	0,00442494	1.37E-45	1
UNC45A	Protein unc-45 homolog A	177,688	0,00647582	1.88E-45	1
GAK	Cyclin-G-associated kinase	177,443	0,0055042	2.56E-45	1
CRYBG3	Beta/gamma crystallin domain-containing protein 3	177,439	8.54E-15	2.57E-45	1

EPS15L1	Epidermal growth factor receptor substrate 15-like 1	177,427	6.26E-15	2.61E-45	1
ASAP1	Arf-GAP with SH3 domain, ANK repeat and PH domain-containing protein 1	177,073	0,00303246	4.07E-45	1
PMPCB	Mitochondrial-processing peptidase subunit beta	176,936	7.64E-14	4.83E-45	1
NUDCD2	NudC domain-containing protein 2	176,726	0,000260434	6.29E-45	1
CFAP36	Cilia- and flagella-associated protein 36	176,043	0,000269468	1.48E-44	1
PIK3R2	Phosphatidylinositol 3-kinase regulatory subunit beta	17,589	7.99E-16	1.79E-44	1
NDUFV2	NADH dehydrogenase [ubiquinone] flavoprotein 2, mitochondrial	175,842	6.37E-14	1.90E-44	1
TRIP4	Activating signal cointegrator 1	173,718	0,000202251	2.64E-44	1
NRIP2	Nuclear receptor-interacting protein 2	17,347	6.02E-13	3.58E-44	1
TMEM209	Transmembrane protein 209	175,249	0,00426199	3.97E-44	1
ARAF	Serine/threonine-protein kinase A-Raf	175,048	0,002929	5.10E-44	1
FKBP5	Peptidyl-prolyl cis-trans isomerase FKBP5	17,49	0,000159799	6.12E-44	1
ARHGEF4 0	Rho guanine nucleotide exchange factor 40	174,375	0,0037725	1.17E-43	1
NDUFS5	NADH dehydrogenase [ubiquinone] iron-sulfur protein 5	174,303	0,00514077	1.28E-43	1
CISD1	CDGSH iron-sulfur domain-containing protein 1	174,271	1.96E-13	1.33E-43	1
DLD	Dihydrolipoyl dehydrogenase, mitochondrial	174,212	0,003928	1.44E-43	1
FAM117B	Protein FAM117B	174,195	0,0102959	1.46E-43	1
NDUFS8	NADH dehydrogenase [ubiquinone] iron-sulfur protein 8, mitochondrial	17,413	0,00871891	1.59E-43	1
ACAD9	Complex I assembly factor ACAD9, mitochondrial	173,957	0,000269792	1.97E-43	1
CCDC120	Coiled-coil domain-containing protein 120	173,925	5.52E-13	2.04E-43	1
PFDN1	Prefoldin subunit 1	173,673	0,0100948	2.79E-43	1
GTPBP10	GTP-binding protein 10	173,584	0,00506977	3.11E-43	1
EVC	EvC complex member EVC	173,573	0,00506274	3.15E-43	1
EEFSEC	Selenocysteine-specific elongation factor	173,322	0,00473049	4.29E-43	1
ATP7A	Copper-transporting ATPase 1	171,427	0,000269338	4.35E-43	1
SUPV3L1	ATP-dependent RNA helicase SUPV3L1, mitochondrial	172,916	0,00508377	7.07E-43	1
ATG2A	Autophagy-related protein 2 homolog A	172,879	0,000269745	7.40E-43	1
KIAA1549	UPF0606 protein KIAA1549	172,703	0,00507245	9.17E-43	1
GPRC5C	G-protein coupled receptor family C group 5 member C	172,644	0,00476643	9.86E-43	1
PARP4	Protein mono-ADP-ribosyltransferase PARP4	172,602	2.01E-15	1.04E-42	1
SKA3	Spindle and kinetochore-associated protein 3	172,574	6.98E-12	1.07E-42	1
FKBP8	Peptidyl-prolyl cis-trans isomerase FKBP8	172,494	1.65E-07	1.18E-42	1
SH3PXD2 A	SH3 and PX domain-containing protein 2A	170,577	0,00770823	1.22E-42	1
TTC9C	Tetratricopeptide repeat protein 9C	172,385	1.52E-12	1.35E-42	1
WDR47	WD repeat-containing protein 47	172,297	0,000269706	1.51E-42	1
SLC4A7	Sodium bicarbonate cotransporter 3	17,187	7.01E-13	2.54E-42	1
PPP1R35	Protein phosphatase 1 regulatory subunit 35	169,911	2.40E-13	2.72E-42	1
SLC25A10	Mitochondrial dicarboxylate carrier	171,539	0,00943688	3.80E-42	1
SPA17	Sperm surface protein Sp17	171,531	0,00506268	3.83E-42	1

Journal of Cell Science • Supplementary information

ALDH6A1	Methylmalonate-semialdehyde dehydrogenase [acylating], mitochondrial	171,482	0,000270683	4.07E-42	1
KLHDC7A	Kelch domain-containing protein 7A	171,344	0,00507056	4.81E-42	1
INPP5F	Phosphatidylinositide phosphatase SAC2	171,107	5.20E-15	6.42E-42	1
NHERF2	Na(+)/H(+) exchange regulatory cofactor NHE-RF2	169,174	0,0101329	6.60E-42	1
RUSC2	AP-4 complex accessory subunit RUSC2	170,815	0,00506385	9.14E-42	1
PHAF1	Phagosome assembly factor 1	170,559	2.71E-07	1.25E-41	1
NCK1	Cytoplasmic protein NCK1	168,634	0,000272302	1.26E-41	1
SPATA2	Spermatogenesis-associated protein 2	170,488	0,00375263	1.36E-41	1
NPHP4	Nephrocystin-4	170,472	1.46E-15	1.38E-41	1
NUP88	Nuclear pore complex protein Nup88	170,398	0,00954468	1.51E-41	1
PNPLA8	Calcium-independent phospholipase A2-gamma	170,329	0,00264549	1.64E-41	1
CEP152	Centrosomal protein of 152 kDa	170,285	0,00366591	1.73E-41	1
NCOA7	Nuclear receptor coactivator 7	170,181	0,0033395	1.97E-41	1
RPGR	X-linked retinitis pigmentosa GTPase regulator	17,014	1.34E-16	2.07E-41	1
MDM1	Nuclear protein MDM1	169,991	0,000202908	2.47E-41	1
TAB1	TGF-beta-activated kinase 1 and MAP3K7-binding protein 1	169,924	4.65E-12	2.68E-41	1
RBSN	Rabenosyn-5	169,491	0,00506967	4.51E-41	1
NCKAP1	Nck-associated protein 1	169,429	0,000268929	4.86E-41	1
FAM83G	Protein FAM83G	169,409	0,00027027	4.98E-41	1
MAP3K11	Mitogen-activated protein kinase kinase kinase 11	169,138	0,000271148	6.89E-41	1
ARHGAP35	Rho GTPase-activating protein 35	168,846	0,00329553	9.78E-41	1
ACADSB	Short/branched chain specific acyl-CoA dehydrogenase, mitochondrial	168,796	0,0001586	1.04E-40	1
WWOX	WW domain-containing oxidoreductase	168,759	0,000270574	1.09E-40	1
CDC20	Cell division cycle protein 20 homolog	168,564	0,00423591	1.37E-40	1
SSX2IP	Afadin- and alpha-actinin-binding protein	168,442	8.62E+00	1.59E-40	1
RGS12	Regulator of G-protein signaling 12	168,247	0,0077155	2.00E-40	1
ERC1	ELKS/Rab6-interacting/CAST family member 1	168,091	0,000314406	2.41E-40	1
MAPRE2	Microtubule-associated protein RP/EB family member 2	166,142	0,00390687	2.43E-40	1
EVC2	Limbin	166,025	5.10E-14	2.79E-40	1
NAPG	Gamma-soluble NSF attachment protein	167,892	0,000144301	3.06E-40	1
CNKSR3	Connector enhancer of kinase suppressor of ras 3	165,933	0,00253898	3.11E-40	1
PLCH1	1-phosphatidylinositol 4,5-bisphosphate phosphodiesterase eta-1	167,794	0,000184068	3.43E-40	1
USP43	Ubiquitin carboxyl-terminal hydrolase 43	167,684	7.03E-12	3.91E-40	1
RPRD1A	Regulation of nuclear pre-mRNA domain-containing protein 1A	167,536	0,0050705	4.67E-40	1
PARD3B	Partitioning defective 3 homolog B	167,526	0,000277957	4.72E-40	1
ASCC2	Activating signal cointegrator 1 complex subunit 2	165,353	8.58E-16	6.13E-40	1
CASKIN2	Caskin-2	167,249	0,00760149	6.56E-40	1
DLG5	Disks large homolog 5	167,173	0,00937564	7.18E-40	1

PFDN6	Prefoldin subunit 6	165,217	0,00507161	7.20E-40	1
METTL13	eEF1A lysine and N-terminal methyltransferase	167,107	0,00035993	7.76E-40	1
CEMIP2	Cell surface hyaluronidase	166,893	1.40E-11	1.00E-39	1
TIMM21	Mitochondrial import inner membrane translocase subunit Tim21	164,815	4.07E-14	1.15E-39	1
OXR1	Oxidation resistance protein 1	166,728	0,0037175	1.22E-39	1
TBC1D31	TBC1 domain family member 31	164,685	0,00414125	1.34E-39	1
SCHIP1	Schwannomin-interacting protein 1	166,639	0,00506414	1.35E-39	1
RAP1GAP	Rap1 GTPase-activating protein 1	166,597	0,00506689	1.42E-39	1
SH3GLB2	Endophilin-B2	16,607	3.00E-12	2.64E-39	1
DCTN6	Dynactin subunit 6	165,838	0,00753446	3.47E-39	1
STK25	Serine/threonine-protein kinase 25	165,581	0,000269439	4.69E-39	1
MIA2	Melanoma inhibitory activity protein 2	165,543	0,0100166	4.91E-39	1
CEP290	Centrosomal protein of 290 kDa	165,332	0,0022782	6.29E-39	1
ANKFY1	Rabankyrin-5	165,269	0,000241958	6.77E-39	1
OCRL	Inositol polyphosphate 5-phosphatase OCRL	165,172	0,00024105	7.59E-39	1
PPP1R13B	Apoptosis-stimulating of p53 protein 1	164,961	0,00252585	9.71E-39	1
RAPGEF2	Rap guanine nucleotide exchange factor 2	164,931	2.64E-17	1.01E-38	1
TOR1AIP2	Torsin-1A-interacting protein 2	164,909	0,00506023	1.03E-38	1
TUT4	Terminal uridylyltransferase 4	164,763	0,00864372	1.22E-38	1
NFKB1	Nuclear factor NF-kappa-B p105 subunit	164,704	0,00868904	1.31E-38	1
MAGI3	Membrane-associated guanylate kinase, WW and PDZ domain-containing protein 3	16,457	0,00258383	1.53E-38	1
FECH	Ferrochelatase, mitochondrial	162,251	3.10E-11	2.25E-38	1
HARS2	Histidine--tRNA ligase, mitochondrial	163,754	0,00868296	3.96E-38	1
VPS26C	Vacuolar protein sorting-associated protein 26C	16,37	0,00791949	4.22E-38	1
PPP1R12C	Protein phosphatase 1 regulatory subunit 12C	163,587	0,00453214	4.81E-38	1
TRIM47	E3 ubiquitin-protein ligase TRIM47	163,583	0,00745289	4.83E-38	1
IQCE	IQ domain-containing protein E	163,408	0,0050791	5.92E-38	1
TSC2	Tuberin	163,361	0,00370307	6.25E-38	1
DENND6A	Protein DENND6A	163,298	4.98E-11	6.72E-38	1
DENND1A	DENN domain-containing protein 1A	162,935	0,0028928	1.02E-37	1
ANKLE2	Ankyrin repeat and LEM domain-containing protein 2	160,764	0,000235108	1.24E-37	1
CEP112	Centrosomal protein of 112 kDa	16,076	0,00025089	1.24E-37	1
RIN3	Ras and Rab interactor 3	16,273	0,00416742	1.30E-37	1
RPS6KB1	Ribosomal protein S6 kinase beta-1	162,649	0,00379501	1.42E-37	1
ATPAF1	ATP synthase mitochondrial F1 complex assembly factor 1	162,538	0,00506107	1.62E-37	1
MTSS1	Protein MTSS 1	162,307	0,0108172	2.11E-37	1
ULK1	Serine/threonine-protein kinase ULK1	162,207	0,000201323	2.37E-37	1
RASSF7	Ras association domain-containing protein 7	162,117	0,00760879	2.63E-37	1
EPN2	Epsin-2	161,967	0,0042558	3.12E-37	1
METTL17	Methyltransferase-like protein 17, mitochondrial	161,744	0,00508658	4.03E-37	1
DIP2A	Disco-interacting protein 2 homolog A	161,533	0,000269511	5.14E-37	1

Journal of Cell Science • Supplementary information

CAMSAP2	Calmodulin-regulated spectrin-associated protein 2	161,384	0,0088624	6.09E-37	1
CELSR2	Cadherin EGF LAG seven-pass G-type receptor 2	161,089	6.85E-13	8.53E-37	1
IFT81	Intraflagellar transport protein 81 homolog	160,909	8.23E-14	1.05E-36	1
PLA2G4A	Cytosolic phospholipase A2	160,558	0,000269415	1.56E-36	1
STXBP5	Syntaxin-binding protein 5	158,207	0,000271942	2.23E-36	1
WDR62	WD repeat-containing protein 62	160,184	0,00507015	2.39E-36	1
ALMS1	Centrosome-associated protein ALMS1	159,996	0,000270287	2.96E-36	1
P4HA2	Prolyl 4-hydroxylase subunit alpha-2	159,855	0,000272618	3.48E-36	1
ZW10	Centromere/kinetochore protein zw10 homolog	159,708	0,00506752	4.10E-36	1
CCDC88A	Girdin	159,672	0,00736939	4.28E-36	1
SEMA4C	Semaphorin-4C	15,75	0,00506352	4.92E-36	1
ARL8A	ADP-ribosylation factor-like protein 8A	159,042	0,000272724	8.71E-36	1
LRRC1	Leucine-rich repeat-containing protein 1	15,89	0,00389505	1.02E-35	1
MADD	MAP kinase-activating death domain protein	158,837	0,000271547	1.10E-35	1
TBC1D8B	TBC1 domain family member 8B	158,792	0,00508057	1.15E-35	1
WDR59	GATOR complex protein WDR59	158,758	0,00875436	1.20E-35	1
MACF1	Microtubule-actin cross-linking factor 1, isoforms 6/7	158,741	0,0038481	1.22E-35	1
NUDCD3	NudC domain-containing protein 3	158,696	0,000269997	1.29E-35	1
ECSIT	Evolutionarily conserved signaling intermediate in Toll pathway, mitochondrial	158,469	0,00316592	1.66E-35	1
TSC1	Hamartin	158,262	0,000275151	2.10E-35	1
CDH6	Cadherin-6	158,202	0,00506874	2.24E-35	1
STX18	Syntaxin-18	157,985	0,00419383	2.86E-35	1
ITPKC	Inositol-trisphosphate 3-kinase C	157,138	0,00731778	7.37E-35	1
LETM1	Mitochondrial proton/calcium exchanger protein	156,568	0,00510509	1.39E-34	1
ADGRG6	Adhesion G-protein coupled receptor G6	156,525	0,000269252	1.46E-34	1
RDX	Radixin	155,684	0,00403503	3.70E-34	1
NFU1	NFU1 iron-sulfur cluster scaffold homolog, mitochondrial	155,479	0,00510098	4.64E-34	1
DHX29	ATP-dependent RNA helicase DHX29	155,189	0,0050627	6.38E-34	1
SEC23IP	SEC23-interacting protein	155,085	0,00027122	7.16E-34	1
EBAG9	Receptor-binding cancer antigen expressed on SiSo cells	154,618	0,00506489	1.19E-33	1
AMOT	Angiomotin	154,415	0,00506557	1.49E-33	1
VPS45	Vacuolar protein sorting-associated protein 45	154,405	0,00767401	1.51E-33	1
GLI3	Transcriptional activator GLI3	154,381	2.73E-13	1.55E-33	1
ARHGAP23	Rho GTPase-activating protein 23	154,379	1.32E-13	1.55E-33	1
NBR1	Next to BRCA1 gene 1 protein	153,097	0,00438547	6.28E-33	1
PIK3R4	Phosphoinositide 3-kinase regulatory subunit 4	152,623	0,00508465	1.05E-32	1
MYO1E	Unconventional myosin-Ie	152,403	0,00518346	1.33E-32	1
AJUBA	LIM domain-containing protein ajuba	151,618	0,00976319	3.11E-32	1
TWF2	Twinfilin-2	151,565	0,000270079	3.29E-32	1
TRAPPC9	Trafficking protein particle complex subunit 9	151,173	0,0050907	5.01E-32	1

Journal of Cell Science • Supplementary information

GRCC10	Protein C10	150,934	0,00506579	6.48E-32	1
PRKCI	Protein kinase C iota type	150,076	0,000271452	1.62E-31	1
EPB41L4A	Band 4.1-like protein 4A	149,529	1.02E-12	2.90E-31	1
ITPRID2	Protein ITPRID2	14,828	0,00925516	1.08E-30	1
EFL1	Elongation factor-like GTPase 1	147,921	0,0247269	1.58E-30	1
LSR	Lipolysis-stimulated lipoprotein receptor	147,768	0,00630371	1.85E-30	1
GMIP	GEM-interacting protein	146,666	0,000228735	5.86E-30	1
IQSEC1	IQ motif and SEC7 domain-containing protein 1	146,311	0,00854	8.48E-30	1
HDAC4	Histone deacetylase 4	144,559	0,00506031	5.17E-29	1
HMGCR	3-hydroxy-3-methylglutaryl-coenzyme A reductase	142,905	0,0246704	2.79E-28	1
RGL2	Ral guanine nucleotide dissociation stimulator-like 2	142,687	0,00507884	3.49E-28	1
VWA8	von Willebrand factor A domain-containing protein 8	140,848	7.78E-10	2.22E-27	1
PIGK	GPI-anchor transamidase	139,586	0,0247615	7.79E-27	1
FAM13B	Protein FAM13B	138,808	0,000269786	1.68E-26	1
ATPSCKMT	ATP synthase subunit C lysine N-methyltransferase	138,544	0,00963088	2.18E-26	1
OFD1	Centriole and centriolar satellite protein OFD1	137,829	4.25E-14	4.40E-26	1
TTC1	Tetratricopeptide repeat protein 1	119,615	0,00624656	7.52E-19	1
URI1	Unconventional prefoldin RPB5 interactor	333,092	0,0118836	0,00312783	1
CEP250	Centrosome-associated protein CEP250	236,159	0,130067	1.11E-82	2
MAP3K2	Mitogen-activated protein kinase kinase kinase 2	232,893	0,142859	2.53E-81	2
PRDM16	Histone-lysine N-methyltransferase PRDM16	20,878	0,0458926	6.08E-64	2
AFAP1L1	Actin filament-associated protein 1-like 1	188,653	0,0476036	1.24E-51	2
VPS36	Vacuolar protein-sorting-associated protein 36	186,763	0,141245	1.53E-50	2
MACROH2A1	Core histone macro-H2A.1	182,836	0,0470209	2.61E-48	2
OCIAD1	OCIA domain-containing protein 1	182,575	0,0478396	3.66E-48	2
RSRC1	Serine/Arginine-related protein 53	180,692	0,146424	4.14E-47	2
TOM1L2	TOM1-like protein 2	180,391	0,0423789	6.08E-47	2
REPS1	RalBP1-associated Eps domain-containing protein 1	180,375	0,0415437	6.21E-47	2
AVEN	Cell death regulator Aven	179,738	0,0475319	1.40E-46	2
AGAP1	Arf-GAP with GTPase, ANK repeat and PH domain-containing protein 1	177,989	0,0415496	1.28E-45	2
SMARCD2	SWI/SNF-related matrix-associated actin-dependent regulator of chromatin subfamily D member 2	177,412	0,137159	2.66E-45	2
TRIP10	Cdc42-interacting protein 4	176,234	0,0395173	1.16E-44	2
GMFB	Glia maturation factor beta	174,148	0,142861	1.55E-44	2
CCDC50	Coiled-coil domain-containing protein 50	176,001	0,0429891	1.56E-44	2
DNM3	Dynamin-3	175,332	0,122903	3.58E-44	2
ERI2	ERI1 exoribonuclease 2	174,308	0,131609	1.27E-43	2
EIF6	Eukaryotic translation initiation factor 6	174,292	0,046302	1.30E-43	2
TRANK1	TPR and ankyrin repeat-containing protein 1	173,798	0,149728	2.39E-43	2

PRKRA	Interferon-inducible double-stranded RNA-dependent protein kinase activator A	173,156	0,041851	5.26E-43	2
RAB8A	Ras-related protein Rab-8A	171,848	0,289579	2.60E-42	2
ACTR2	Actin-related protein 2	171,762	0,141297	2.89E-42	2
GPD1L	Glycerol-3-phosphate dehydrogenase 1-like protein	170,965	0,0433535	7.62E-42	2
TRIOBP	TRIO and F-actin-binding protein	170,867	0,127831	8.58E-42	2
PPOX	Protoporphyrinogen oxidase	170,714	0,140994	1.03E-41	2
NT5C3B	7-methylguanosine phosphate-specific 5'-nucleotidase	170,692	0,143546	1.06E-41	2
HARS1	Histidine--tRNA ligase, cytoplasmic	170,521	0,0466739	1.30E-41	2
NDC1	Nucleoporin NDC1	170,082	0,0464077	2.22E-41	2
MX1	Interferon-induced GTP-binding protein Mx1	170,044	0,300582	2.32E-41	2
OLA1	Obg-like ATPase 1	16,792	0,054942	2.96E-41	2
ARF4	ADP-ribosylation factor 4	169,132	0,14973	6.94E-41	2
OGA	Protein O-GlcNAcase	168,727	0,154857	1.13E-40	2
CIAPIN1	Anamorsin	168,442	0,118067	1.59E-40	2
DCTN4	Dynactin subunit 4	168,344	0,0460559	1.78E-40	2
TBC1D4	TBC1 domain family member 4	168,203	0,138442	2.11E-40	2
PIMREG	Protein PIMREG	168,026	0,143315	2.60E-40	2
ALKBH5	RNA demethylase ALKBH5	167,998	0,150468	2.69E-40	2
OXA1L	Mitochondrial inner membrane protein OXA1L	167,895	0,147629	3.05E-40	2
NDUFA5	NADH dehydrogenase [ubiquinone] 1 alpha subcomplex subunit 5	167,852	0,0392775	3.20E-40	2
YARS2	Tyrosine--tRNA ligase, mitochondrial	167,753	0,0468829	3.61E-40	2
SPATA2L	Spermatogenesis-associated protein 2-like protein	167,386	0,0264744	5.58E-40	2
FAM162A	Protein FAM162A	167,124	0,290238	7.61E-40	2
BCL2L13	Bcl-2-like protein 13	166,926	0,15593	9.62E-40	2
GPS1	COP9 signalosome complex subunit 1	166,699	0,0461245	1.26E-39	2
LEMD2	LEM domain-containing protein 2	164,739	0,0435211	1.26E-39	2
KRT6A;KRT6B	Keratin, type II cytoskeletal 6A	165,697	0,308032	4.09E-39	2
SPCS2	Signal peptidase complex subunit 2	165,565	0,0438879	4.79E-39	2
	UPF0688 protein C1orf174 homolog	165,319	0,051945	6.38E-39	2
PSME2	Proteasome activator complex subunit 2	164,865	0,136839	1.09E-38	2
IFT43	Intraflagellar transport protein 43 homolog	164,591	0,0426846	1.50E-38	2
CDC42BPG	Serine/threonine-protein kinase MRCK gamma	164,204	0,131585	2.35E-38	2
SLC9A1	Sodium/hydrogen exchanger 1	164,203	0,0460065	2.35E-38	2
GRK2	Beta-adrenergic receptor kinase 1	163,511	0,0441231	5.26E-38	2
RNH1	Ribonuclease inhibitor	163,254	0,129999	7.07E-38	2
PHF5A	PHD finger-like domain-containing protein 5A	163,071	0,0461368	8.75E-38	2
EMC4	ER membrane protein complex subunit 4	163,008	0,159726	9.41E-38	2
ARPC5L	Actin-related protein 2/3 complex subunit 5-like protein	162,386	0,297875	1.93E-37	2
CCDC142	Coiled-coil domain-containing protein 142	162,324	0,0508367	2.07E-37	2
RAB12	Ras-related protein Rab-12	162,062	0,142417	2.80E-37	2
PGP	Glycerol-3-phosphate phosphatase	162,034	0,0450682	2.89E-37	2

Journal of Cell Science • Supplementary information

PRC1	Protein regulator of cytokinesis 1	160,928	0,133689	1.03E-36	2
SLC25A11	Mitochondrial 2-oxoglutarate/malate carrier protein	160,815	0,162513	1.17E-36	2
PSMD12	26S proteasome non-ATPase regulatory subunit 12	160,668	0,0460016	1.38E-36	2
CLTA	Clathrin light chain A	160,601	0,309693	1.49E-36	2
TROAP	Tastin	160,258	0,152688	2.20E-36	2
DCP1A	mRNA-decapping enzyme 1A	159,936	0,137911	3.17E-36	2
TACC1	Transforming acidic coiled-coil-containing protein 1	159,932	0,0387071	3.19E-36	2
GNG12	Guanine nucleotide-binding protein G(I)/G(S)/G(O) subunit gamma-12	159,609	0,0385663	4.59E-36	2
PIKFYVE	1-phosphatidylinositol 3-phosphate 5-kinase	159,569	0,0424001	4.81E-36	2
ATAT1	Alpha-tubulin N-acetyltransferase 1	15,941	0,144023	5.75E-36	2
MAP4K2	Mitogen-activated protein kinase kinase kinase kinase 2	159,248	0,151419	6.91E-36	2
PALM3	Paralemmin-3	158,586	0,0422453	1.46E-35	2
CDKN2A	Cyclin-dependent kinase inhibitor 2A	15,853	0,297767	1.55E-35	2
CEP68	Centrosomal protein of 68 kDa	15,847	0,0450077	1.66E-35	2
ZFP62	Zinc finger protein 62	156,279	0,048507	1.92E-35	2
PPP6R1	Serine/threonine-protein phosphatase 6 regulatory subunit 1	158,318	0,0450406	1.97E-35	2
PPP6R3	Serine/threonine-protein phosphatase 6 regulatory subunit 3	158,197	0,054737	2.25E-35	2
PDE4C	cAMP-specific 3',5'-cyclic phosphodiesterase 4C	157,955	0,132667	2.96E-35	2
PLEKHH2	Pleckstrin homology domain-containing family H member 2	157,622	0,131262	4.29E-35	2
RASSF8	Ras association domain-containing protein 8	156,632	0,128082	1.29E-34	2
FCF1	rRNA-processing protein FCF1 homolog	156,496	0,0469729	1.50E-34	2
BOLA1	BolA-like protein 1	156,111	0,156335	2.31E-34	2
ECI2;ECI3	Enoyl-CoA delta isomerase 3, peroxisomal	155,191	0,125792	6.37E-34	2
ZC3H3	Zinc finger CCCH domain-containing protein 3	155,127	0,0436113	6.83E-34	2
GLIPR2	Golgi-associated plant pathogenesis-related protein 1	155,009	0,147038	7.78E-34	2
CLPTM1	Putative lipid scramblase CLPTM1	151,838	0,0482461	2.45E-33	2
ANXA6	Annexin A6	153,582	0,0500165	3.71E-33	2
ZNF24	Zinc finger protein 24	15,353	0,0425403	3.93E-33	2
GALNT2	Polypeptide N-acetylgalactosaminyltransferase 2	153,176	0,292805	5.77E-33	2
GINS3	DNA replication complex GINS protein PSF3	153,124	0,128566	6.11E-33	2
EMC3	ER membrane protein complex subunit 3	152,807	0,307625	8.61E-33	2
GCAT	2-amino-3-ketobutyrate coenzyme A ligase, mitochondrial	152,591	0,127774	1.09E-32	2
PPID	Peptidyl-prolyl cis-trans isomerase D	152,222	0,129824	1.62E-32	2
NOP53	Ribosome biogenesis protein NOP53	152,185	0,160028	1.69E-32	2
PSMA6	Proteasome subunit alpha type-6	151,884	0,131036	2.34E-32	2
GABPB2	GA-binding protein subunit beta-2	151,045	0,14725	5.75E-32	2
ARF5	ADP-ribosylation factor 5	150,924	0,305672	6.55E-32	2
AFG3L1	AFG3-like protein 1	150,775	0,300013	7.68E-32	2
SDHAF2	Succinate dehydrogenase assembly factor 2, mitochondrial	150,681	0,147664	8.49E-32	2
KIF20B	Kinesin-like protein KIF20B	150,087	0,150851	1.60E-31	2

Journal of Cell Science • Supplementary information

AP3D1	AP-3 complex subunit delta-1	148,914	0,127242	5.55E-31	2
RRAGC	Ras-related GTP-binding protein C	14,69	0,104804	4.60E-30	2
USP9X	Probable ubiquitin carboxyl-terminal hydrolase FAF-X	14,637	0,153976	7.98E-30	2
OGFR	Opioid growth factor receptor	14,511	0,141423	2.94E-29	2
STX12	Syntaxin-12	144,398	0,0247989	6.10E-29	2
ECD	Protein ecdysoneless homolog	141,498	0,0481099	1.16E-27	2
IDH1	Isocitrate dehydrogenase [NADP] cytoplasmic	140,711	0,115751	2.54E-27	2
RAD21	Double-strand-break repair protein rad21 homolog	140,137	0,0374965	4.51E-27	2
KIFC2	Kinesin-like protein KIFC2	139,808	0,124499	6.25E-27	2
SEC62	Translocation protein SEC62	139,172	0,0450312	1.17E-26	2
SGTA	Small glutamine-rich tetratricopeptide repeat-containing protein alpha	138,442	0,135579	2.41E-26	2
MAP3K1	Mitogen-activated protein kinase kinase kinase 1	137,776	0,043602	4.63E-26	2
ZNF276	Zinc finger protein 276	137,558	0,150146	5.73E-26	2
GEMIN5	Gem-associated protein 5	13,55	0,0515402	4.21E-25	2
SPIRE2	Protein spire homolog 2	134,703	0,0831959	9.05E-25	2
DKC1	H/ACA ribonucleoprotein complex subunit DKC1	133,042	0,0502854	4.39E-24	2
ATL2	Atlastin-2	132,412	0,140091	7.95E-24	2
APP	Amyloid-beta precursor protein	132,095	0,0446246	1.07E-23	2
OAT	Ornithine aminotransferase, mitochondrial	130,534	0,120772	4.59E-23	2
WASHC1	WASH complex subunit 1	324,016	0,259406	0,00393447	2

Journal of Cell Science • Supplementary information
