# Supplementary material for: The EH-binding protein EHBP1 operates in a ciliary functional module affected by INPP5E dysfunction
Source: J Cell Sci. 2026 Jul 21;139(14):jcs264091. doi: 10.1242/jcs.264091 (PMC13450894; doi:10.1242/jcs.264091)
Supplement: Supplementary file 2 [file joces-139-264091_review_history.pdf]

Journal of Cell Science

The EH-binding protein EHBP1 operates in a ciliary functional module affected by INPP5E dysfunction

Kae R. Whiting, Mariam Aslanyan, Imke Peters, Stef Letterboer, Ideke J. C. Lamers, Mohamed-Ali Jarboui, Tina Beyer, Katrin Dahlke, Karsten Boldt and Ronald Roepman
DOI: 10.1242/jcs.264091

Editor: Pleasantine Mill

Review timeline

Original submission:	16 April 2025
Editorial decision:	10 June 2025
First revision received:	4 September 2025
Editorial decision:	12 September 2025
Second revision received:	16 February 2026
Accepted:	22 February 2026

Original submission

First decision letter

MS ID#: jcs.264091

MS TITLE: The EH Binding protein EHBP1 operates in a ciliary functional module affected by INPP5E dysfunction

AUTHORS: Kae R Whiting; Mariam G Aslanyan; Imke Peters; Stef J F Letterboer; Mohamed-Ali Jarboui; Tina Beyer; Katrin Dahlke; Karsten Boldt; Ronald Roepman

ARTICLE TYPE: Research Article

Dear Ronald and team,

Thank you for submitting to our Special Issue on Cilia and Flagella for the Journal of Cell Science- we have now reached a decision on your manuscript.

To see the reviewers' reports and a copy of this decision letter, please go to:

This represents a novel and intriguing non-biased approach to understand interactions, including transient ones, of the the key phosphoinositide content regulator INPP5E targeting the ciliary compartment. Here Whiting et al find top hits including an actin and phosphoinosite binding protein EHBP1 which mediates membrane tubulation with either RAB8 and RAB10, exocytic RAB GTPases both linked to ciliary membrane trafficking and exocyst dependent release. However, EHBP1 itself has never been linked directly to cilia, which makes this study novel and broadly interesting even without clear function.

Both reviewers and this editor raise points around two key features that will need addressing prior to publication in JCS: (1) specificity of the staining/lack of staining in IMCD3 cells where target was identified as ciliary interactor of INPP5E and that it is dynamic; (2) toning down claims in photoreceptor organoids that this relates to retinal function as no function for EHBP1 is shown in this manuscript. In addition to the list of comments and edits that should be addressed from both reviewers, I have some suggestions editorially:

© 2026. Published by The Company of Biologists under the terms of the Creative Commons Attribution License (https://creativecommons.org/licenses/by/4.0/).

(1) Introduction line 47-48: consider ease of reading by changing to 'multi-organ syndromic conditions including Joubert and MORM syndromes'.
(2) Introduction line 53: delete 'along the gene' superfluous.
(3) Introduction line 54: consider changing 'phenotypes' to 'presentations'.
(4) Introduction line 72: Cite the paper where the iPSCs from the Theil lab were generated.
(5) Methods line 84: where were hTERT-RPE-1 cells from? Were all cell lines shown to be mycoplasm free?
(6) Methods line 94 and throughout- italicize knock-in cell line- are they protein null? They are referred to as knock-out, but are homozygous for a missense?
(7) Methods line 161: missing details for protein lysis and loading conditions (buffers, gels etc) for WB used in the manuscript.
(8) Methods line 213: How where images acquired- blinded on DAPI? On cilia/ARL13B+- before manually assessing basal body vs cilia compartment. EHBP1 is largely described in the field as being endomenbranous, including by Proteintech who makes the antibody.
(9) Methods line 214: Expand in the text what is meant by biological and techinical replicates in your experimental design.
(10) Results line 237-239 (and throughout): All mouse proteins should be ALL CAPs here in text, Figure 1 and its legends.
(11) Results line 255-257: should also describe roles described with another cilia-associated RAB RAB10 https://doi.org/10.1083/jcb.202410003; DOI: 10.1152/ajprenal.00198.2010; https://doi.org/10.1371/journal.pgen.1006093.
(12) Results line 259-274 and Figure 2: given this is the first description of EHBP-1 at basal body/cilia versus its predominantly cited endomenbranous localization, it is important to show levels at basal bodies and cilia compared to other cellular compartments. Specificity of the antibody- KD by IF or tagged O/E would be very helpful here. Expanded discussion is needed to explain the IMCD3 results- it could be that in fact the antibody does not recognize mouse EHBP-1 by IF- line 271 as it stands is concerning as INPP5E biotinylates EHBP1 presumably at the cilium in Figure 1 of this paper in IMCD3 cells.
(13) Figure legends Figure 2 Line 603: Detail is needed to explain how cells with both basal body and axoneme are scored, as you have examples of those with just basal body or just cilia. Similarly no localization (line 606) to the cilium- this means both BB and axoneme? Would benefit from clarification.
(14) Throughout- watch italics for the patient alleles throughout the text, figure labels and legends.
(15) Figure S1- why are the cilia so different in A and B in terms of length- both IMCD3 cells? Clarify that constructs in D are human EHBP1 or mouse? Does the GFP-tagged construct localize to centrosome and cilia- see point 12.

(16) Data accessibility- we strongly recommend that the dataset for the proximity proteomics be uploaded to PRIDE or other databases and an accession number provided helps maximize impact of your work and reuse for others.

As you will see, there are several concerns from reviewers that prevent me from accepting the paper at this stage. They all feel, however, that a revised version would be acceptable, if you can address their concerns. If you think that you can deal satisfactorily with the criticisms on revision, I would be pleased to see a revised manuscript. We would then return it to the reviewers.

Reviewer 1

Advance summary and potential significance to field

Summary

In the manuscript by Whiting et al., proximity-labeled proteomics was used to identify new INPP5E-associated proteins. The manuscript focuses on endocytic adaptor EH domain binding protein (EHBP1), a protein found predominantly in the basal body in ciliated cultured cells, but some cell type differences in localization. The authors extend their analysis of EHBP1 to retinal organoids differentiated from induced pluripotent stem cells. Disrupting INPP5E leads to a minor localization change in primary cilia and disrupts the immunofluorescence signal in organoid cells. The characterization of EHBP1 as a new ciliary protein related to INPP5E is interesting, but there was no

© 2026. Published by The Company of Biologists under the terms of the Creative Commons Attribution License (https://creativecommons.org/licenses/by/4.0/).

function for EHBP1 found. The authors attempt to use retinal organoids to assign some possible photoreceptor function to EHBP1, but these results are unconvincing and do not have any relevance to photoreceptor neurons that develop and function in in vivo retinas. Conclusions from the data are often overstated, and critically, many of the performed measurements and analyses are poorly described and difficult to interpret. The manuscript is judged to be incomplete. Any publication with this data would require significant edits that more accurately describe and interpret the results.

Major comments

The authors assume that EHBP1 passages from the BB, where it is predominantly found, and into the cilium; however, this was not shown. The authors should test this experimentally. Similarly, the authors describe EHBP1 as a dynamic ciliary protein (line #15). But dynamic is a vague term that needs clarification.

The "Quantification and statistical analysis" section of the Methods section is insufficient. All analyses and measures should be completely described. For example, the area measurement parameters in retinal organoids presented in Fig. 4 are not described. Another example: Is Fig. 2D data percentages of BB fluorescence signal/total signal? Or is this a percent of cilia with these localization scores? Since there are some "no localization" results, one can guess the latter. Does this mean that in BB localization instances there is no EHBP1 at all along the axoneme? Another example: In Fig. 3D, how were the intensity measurements calculated? How was the BB region determined?

Figure 4 describes "outer segments" in retinal organoids, but these are not outer segments. Outer segments are a specialized cilium with stacks of membrane discs containing phototransduction proteins. In line 386 the term "outer membrane" is used, which is more appropriate.

Minor comments

The title is not accessible and should be reconsidered. Specifically, the term "ciliary functional module" is another vague term. The term again in line 79, where it is again confusing and possibly misleading.

It is unclear if the particular INPP5E-KD (knockdown) retinal organoids used in this study were generated in prior studies. If not, the authors should validate the knockdown/knockout here.

In line 20 of the abstract, the authors should not suggest that EHBP1 functions in photoreceptor cilia, since they do not provide enough evidence for this suggestion. Instead, they could conclude that EHBP1 may have some role in photoreceptor cells (broadly) and save speculation about the CC and OS for other sections.

Line 55 - The point about most proteins forming a complex is unnecessary and should be removed.

Line 75 states that EHBP1 was localized to the outer segment membrane of photoreceptors, which is inaccurate. Similar: Line 303 - the title of the section "EHBP1 localizes to the outer segment membrane..." is inaccurate since there are no outer segments here.

In Fig. 2A-C it is difficult to see the localization of EHBP1 immunostaining due to the green and cyan overlay. The authors should add just the EHBP1 channel + DAPI

Can the authors clarify the point of the Fig. 2E and Fig. 2F analyses? EHBP1 fluorescence intensity relative to a standard ciliary/basal body marker is needed for the comparison.

Line 309 states that "control" retinal organoids were used to determine the localization of EHBP1 in the developing retina and photoreceptors. What are control retinal organoids, and is there literature evidence that proves that they contain human-like photoreceptors? Then, on line 311, "mature" organoids containing "maturing" photoreceptors are described. This needs clarification with literature support. Are the outermost cells in retinal organoids equivalent to developing photoreceptor neurons?

© 2026. Published by The Company of Biologists under the terms of the Creative Commons Attribution License (https://creativecommons.org/licenses/by/4.0/).

Fig. S4 A, B: Why would EHBP1, a BB protein, be so diffusely localized in organoid cells? Pericentrin is not clearly shown in these images. Why weren't PCTN and ARL13B immunostaining used in "mature" organoids?

Line 325 - The authors conclude that there is a mild trafficking defect in INPP5E mutant fibroblasts based on Fig. 2C,D because slightly fewer cilia had EHBP1 along the axoneme. EHBP1 is predominantly at the BB in all conditions, so it is hard to understand the significance of this conclusion.

Line 299 is a long sentence that should be rewritten for readability.

Line 392 - The authors make an excessive stretch by suggesting a potential role for EHBP1 in OS disc phagocytosis. None of their data is close to supporting this suggestion. It should be removed.

Line 414 describes data that are not shown. The authors must explain why this data was not included.

Reviewer 2

Advance summary and potential significance to field

This manuscript investigates the localization and regulation of EHBP1 in the context of INPP5E function by combining proximity labeling proteomics with immunofluorescence-based localization studies across cellular and organoid-based models. The authors identify EHBP1 as a novel, previously uncharacterized proximity interactor of INPP5E using miniTurbo-based biotinylation in IMCD3 cells, and subsequently assess EHBP1 localization in human RPE cells, fibroblasts, and iPSC-derived retinal organoids through immunofluorescence microscopy. They explore how actin cytoskeleton disruption and INPP5E deficiency affect EHBP1 positioning at the basal body, within the primary cilium, and in photoreceptor outer segment-like structures. Together, these data suggest that EHBP1 may participate in a trafficking module influenced by INPP5E function and actin organization. However, the retinal organoid data, which are central to the manuscript's claim that EHBP1 is regulated by INPP5E in photoreceptors, are less conclusive. Several key aspects of the organoid analysis require additional validation, particularly concerning INPP5E knockout status, the identification of outer segment structures, and the specificity of EHBP1 mis localization. These concerns, if addressed, would significantly strengthen the manuscript's message.

Major Points
EHBP1 was identified as the most significantly enriched interactor in the INPP5E proximity proteomics screen conducted in IMCD3 Flp-In cells, yet the authors report no detectable ciliary localization of EHBP1 in IMCD3 cells by immunofluorescence. This discrepancy warrants further clarification. Could EHBP1 be interacting with a non-ciliary pool of INPP5E, or engaging transiently during trafficking events not captured by fixed-cell imaging? A brief discussion of this point would help reconcile the proteomic and localization data.
Another concern is that the authors do not demonstrate that INPP5E is actually knocked out in the retinal organoids described throughout Figure 4 and Figure S4. No protein or transcript-level validation of INPP5E loss is provided. Without this, the interpretation of EHBP1 mis localization or changes in expression cannot be confidently attributed to INPP5E deficiency. This is a critical omission, as the central premise of the retinal organoid analysis rests on the comparison between control and INPP5EKD.
A second major issue arises from the appearance of photoreceptor outer segments in INPP5EKD organoids at d230. The structures interpreted as outer segments in Figure 4 appear more prominent or expanded in knockdown than in controls. This contradicts existing in vivo data from INPP5E knockout mouse models, which consistently show very rapid outer segment disorganization or degeneration. Before concluding that EHBP1 fails to localize to photoreceptor outer segments, the authors must first confirm that these structures are indeed bona fide OS. This could be achieved by immunostaining for other canonical OS markers. Without this, the observed absence of EHBP1 in the OS could reflect a loss or alteration of outer segment identity, rather than a trafficking defect.

© 2026. Published by The Company of Biologists under the terms of the Creative Commons Attribution License (https://creativecommons.org/licenses/by/4.0/).

In several organoid images (e.g., Figure S4), EHBP1 appears reduced in the OS layer of INPP5EKD organoids but is increased in deeper retinal layers. However, no effort is made to identify which retinal cell types are expressing EHBP1 in these locations. Without co-staining for cell-type markers such as CRX (photoreceptors), CHX10 (retinal progenitors), or BRN3A (ganglion cells), the interpretation that EHBP1 is mis localized within photoreceptors remains speculative. The increase in EHBP1 signal in the inner retina may reflect a shift in expression to another cell type or a structural reorganization within the tissue.
Lastly, there appears to be a qualitative reduction in ARL13B staining in INPP5EKD organoids, raising the possibility that cilia formation or stability is impaired more broadly (Fig S4). This should be quantified by measuring ARL13B-positive cilia frequency, length, and intensity across photoreceptor cell layers. Without this information, it remains unclear whether EHBP1 loss from the cilium is a specific trafficking defect or part of a more general ciliary disruption.

Minor Points
The manuscript uses the term "trafficking defect" to describe EHBP1 behavior in retinal organoids. This interpretation should be stated more cautiously, particularly since the structural presence of outer segments and cilia has not yet been confirmed in INPP5EKD conditions.
In Figure 3, the cell line used for Cytochalasin D treatment is not clearly stated in the figure legend and should be explicitly identified as human RPE. For consistency and clarity, the biological system used in each figure should be clearly labeled.
Several figures, especially those involving retinal organoids, would benefit from improved side-by-side comparisons. In some cases, differences in image brightness or focal depth complicate the interpretation of morphological differences between control and knockout samples.
The authors report changes in EHBP1 signal across different retinal layers, but these observations are not quantified. Including mean fluorescence intensity measurements or area-based quantification for both outer and inner layers would add rigor to the interpretation.

First revision

Author response to reviewers' comments

Point-by-point responses to the Reviewers' comments

First of all, we would like to warmly thank the Editor and the reviewers for their feedbacks and appreciate their comments and suggestions. These gave us the chance to improve our manuscript, include critical data and improve our discussion. All these points are addressed below.

Several of the comments are answered by a recent BioRxiv pre-print:

"Loss of INPP5E affects photoreceptor outer segment membrane biogenesis in iPSC-derived human retinal organoids." Whiting, K.R., Aslanyan, M.G., Beyer, T., Dahlke, K., Boldt, K., and Roepman, R. (**BIORXIV/2025/672060**).

From the Editor :

Both reviewers and this editor raise points around two key features that will need addressing prior to publication in JCS: (1) specificity of the staining/lack of staining in IMCD3 cells where target was identified as ciliary interactor of INPP5E and that it is dynamic; (2) toning down claims in photoreceptor organoids that this relates to retinal function as no function for EHBP1 is shown in this manuscript.

Response:
(1) This is an important point that we have addressed in the reviewers and editors comments. Briefly, western blot analysis of IMCD3 cells indicates less specific binding of the EHBP1 antibody in mouse cells compared to human cells, highlighting the possible reason no ciliary staining in IMCD3 cells.

© 2026. Published by The Company of Biologists under the terms of the Creative Commons Attribution License (https://creativecommons.org/licenses/by/4.0/).

(2) The claims of EHBP1 function within the retinal organoids and photoreceptors have been toned down throughout the text.

In addition to the list of comments and edits that should be addressed from both reviewers, I have some suggestions editorially:

(1) Introduction line 47-48: consider ease of reading by changing to 'multi-organ syndromic conditions including Joubert and MORM syndromes'.

Response:
The text has been adjusted as suggested (Lines 47-49)

(2) Introduction line 53: delete 'along the gene' superfluous.

Response:
The text has been adjusted as suggested (Line 52)

(3) Introduction line 54: consider changing 'phenotypes' to 'presentations'.

Response:
The text has been adjusted as suggested (Line 54)

(4) Introduction line 72: Cite the paper where the iPSCs from the Theil lab were generated.

Response:
Citations to the papers have been added (Line 71)

(5) Methods line 84: where were hTERT-RPE-1 cells from? Were all cell lines shown to be mycoplasm free?

Response:

hTERT-RPE-1 cells were bought from ATCC (Line 83). All cell lines used in this study were tested for mycoplasm and confirmed to be mycoplasm free. This is now indicated in the text (Line 99).

(6) Methods line 94 and throughout- italicize knock-in cell line- are they protein null? They are referred to as knock-out, but are homozygous for a missense?

Response:
Cell line has been italicized throughout. For clarification, the description of the iPSC line has been updated as "iPSC lines containing a homozygous D477N mutation in INPP5E, an enzymatically null loss-of-function mutation hereby referred to as *INPP5E*^{KD}" (Lines 93-94).

(7) Methods line 161: missing details for protein lysis and loading conditions (buffers, gels etc) for WB used in the manuscript.

Response:
Methods section has been updated to include a more thorough explanation of cell lysis and western blot (Lines 163-178)

(8) Methods line 213: How where images acquired- blinded on DAPI? On cilia/ARL13B+- before manually assessing basal body vs cilia compartment. EHBP1 is largely described in the field as being endomembranous, including by Proteintech who makes the antibody.

Response:
Images were acquired based on cilia marker to ensure at least 10 cilia were in each field of view before manual assessment of EHBP1 expression at the basal body or within the ciliary compartment. The methods section for image acquisition and quantification has been rewritten to

© 2026. Published by The Company of Biologists under the terms of the Creative Commons Attribution License (https://creativecommons.org/licenses/by/4.0/).

reflect this (Lines 228-232).

(9) Methods line 214: Expand in the text what is meant by biological and technical replicates in your experimental design.

Response:
Experimental setup of biological and technical replicates is clarified for cell lines (lines 229-230) and retinal organoid experiments (lines 237-238).

(10) Results line 237-239 (and throughout): All mouse proteins should be ALL CAPs here in text, Figure 1 and its legends.

Response:
All mouse protein names have been updated to be in all caps in text and in figures.

(11) Results line 255-257: should also describe roles described with another cilia-associated RAB RAB10 https://doi.org/10.1083/jcb.202410003; DOI: 10.1152/ajprenal.00198.2010; https://doi.org/10.1371/journal.pgen.1006093.

Response:
Thank you for the suggestion. The role of RAB10 has been included (lines 281-282) and the proposed papers have been cited.

(12) Results line 259-274 and Figure 2: given this is the first description of EHBP-1 at basal body/cilia versus its predominantly cited endomembranous localization, it is important to show levels at basal bodies and cilia compared to other cellular compartments. Specificity of the antibody- KD by IF or tagged O/E would be very helpful here. Expanded discussion is needed to explain the IMCD3 results- it could be that in fact the antibody does not recognize mouse EHBP-1 by IF- line 271 as it stands is concerning as INPP5E biotinylates EHBP1 presumably at the cilium in Figure 1 of this paper in IMCD3 cells

Response:
To better understand the lack of cilia specific staining in the IMCD3 cells, we performed a western blot analysis on hTERT-RPE-1 cells and IMCD3 cells using the EHBP1 antibody (Figure S1E. A faint band can be seen in the IMCD3 cells at 150 kDa, however compared to RPE cells and HEK293T (Fig S1D) there is an increase in non-specific bands in IMCD3 cells. This highlights the possibility that the EHBP1 antibody shows less specificity in mouse IMCD3 cells than in human cells, which is in line with the non-specific membrane staining in the IMCD3 cells.

(13) Figure legends Figure 2 Line 603: Detail is needed to explain how cells with both basal body and axoneme are scored, as you have examples of those with just basal body or just cilia. Similarly no localization (line 606) to the cilium- this means both BB and axoneme? Would benefit from clarification.

Response:
All quantification was completed manually. The quantification and statistical analysis has been updated to include a more thorough explanation of how images were quantified (Lines 227-27). Additionally, the ciliary cutouts of Figure 2 have been adjusted to include examples of basal body only, ciliary compartment, and no ciliary localization.

(14) Throughout- watch italics for the patient alleles throughout the text, figure labels and legends.

Response:
Figures and text referring to the patient fibroblasts have been checked and adjusted when required throughout the text.

(15) Figure S1- why are the cilia so different in A and B in terms of length- both IMCD3 cells? Clarify that constructs in D are human EHBP1 or mouse? Does the GFP-tagged construct localize to centrosome and cilia- see point 12.

© 2026. Published by The Company of Biologists under the terms of the Creative Commons Attribution License (https://creativecommons.org/licenses/by/4.0/).

Response:
Cilia in IMCD3 cells generally have a large variety in length, and this can be further affected by the confluency of cells when serum starvation occurs, the confluency of cells in each field of view imaged, and how the cilia are positioned within the field of view. These are representative images of the staining, however, so this variety is not always as clear.

The construct in S1D is human. The figure legend of Figure S1 has been adjusted accordingly (Line 685).

IMCD3 cells transfected with GFP-EHBP1, as well as Flag- and HA-tagged EHBP1, all have a low transfection efficiency and have non-specific expression throughout the cellular cytoplasm. This indicates issues with transient overexpression of this recombinant protein. We show these data below for your review, but do not think it is informative for the paper therefore we did not include the data (see below).

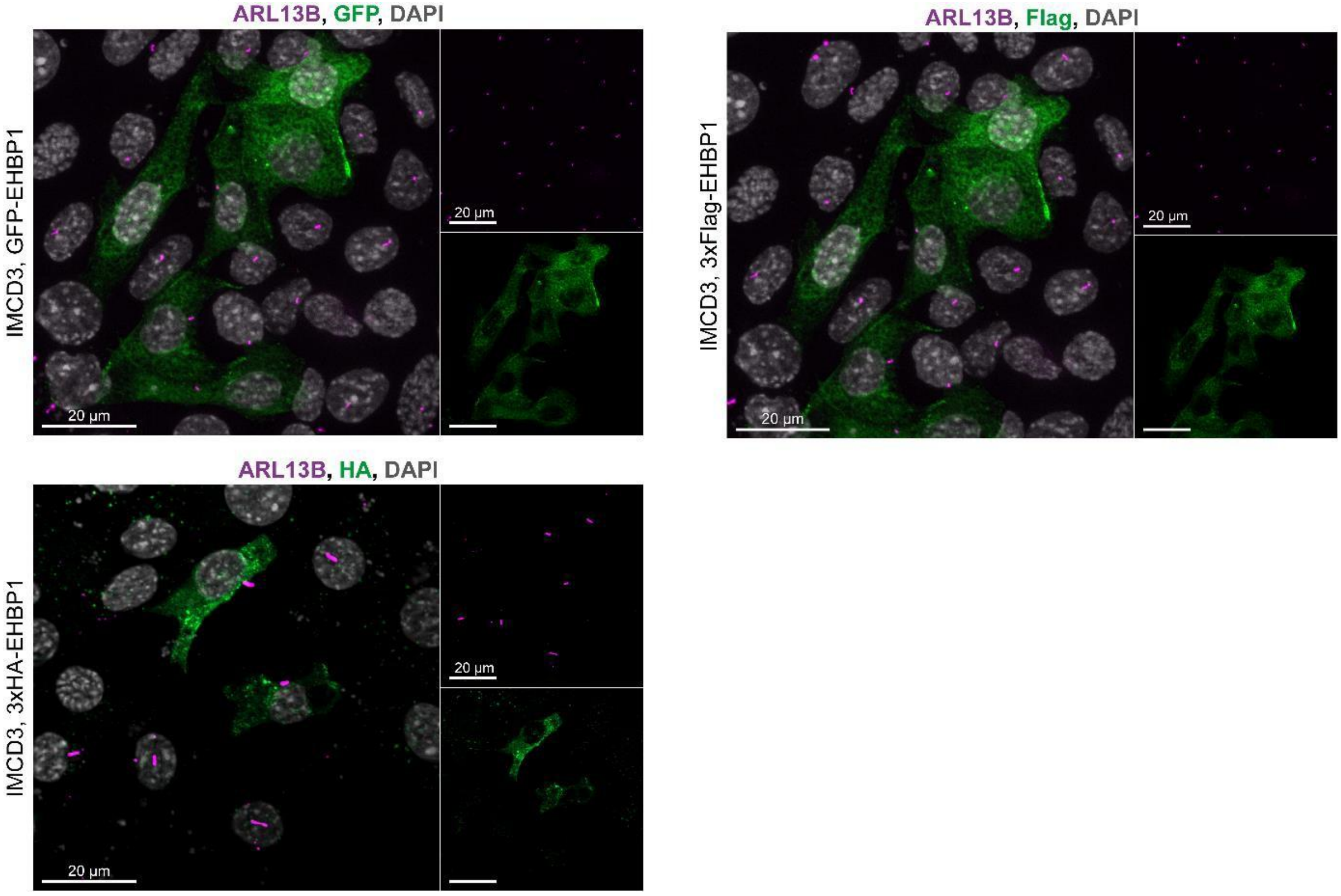

(16) Data accessibility- we strongly recommend that the dataset for the proximity proteomics be uploaded to PRIDE or other databases and an accession number provided helps maximize impact of your work and reuse for others.

Response:
When accepted for publication, proximity proteomics data will be uploaded to PRIDE, as recommended.

From Reviewer 1:

Major comments:
The authors assume that EHBP1 passages from the BB, where it is predominantly found, and into the cilium; however, this was not shown. The authors should test this experimentally. Similarly, the authors describe EHBP1 as a dynamic ciliary protein (line #15). But dynamic is a vague term that needs clarification.

Response:
The reviewer brings up an important point. Unfortunately due to a lack of resources to perform live-

© 2026. Published by The Company of Biologists under the terms of the Creative Commons Attribution License (https://creativecommons.org/licenses/by/4.0/).

cell imaging, and due to non-specific localization of overexpressed GFP-EHBP1 construct in cells (see: point 15 above), we are unable to verify the movement of EHBP1 from the basal body to the cilium. Instead, we have adjusted the wording when describing EHBP1 localization at the cilium to reflect this. We have also removed the use of dynamic in line 15.

The "Quantification and statistical analysis" section of the Methods section is insufficient. All analyses and measures should be completely described. For example, the area measurement parameters in retinal organoids presented in Fig. 4 are not described. Another example: Is Fig. 2D data percentages of BB fluorescence signal/total signal? Or is this a percent of cilia with these localization scores? Since there are some "no localization" results, one can guess the latter. Does this mean that in BB localization instances there is no EHBP1 at all along the axoneme? Another example: In Fig. 3D, how were the intensity measurements calculated? How was the BB region determined?

Response:
The quantification and statistical analysis has been rewritten to include a more detailed description for each analysis and measurement performed (lines 227-247)

Figure 4 describes "outer segments" in retinal organoids, but these are not outer segments. Outer segments are a specialized cilium with stacks of membrane discs containing phototransduction proteins. In line 386 the term "outer membrane" is used, which is more appropriate.

Response:
As recommended, we have adjusted the use of “outer segments” to “photoreceptor outer membrane” throughout the text.

Minor comments:

The title is not accessible and should be reconsidered. Specifically, the term "ciliary functional module" is another vague term. The term again in line 79, where it is again confusing and possibly misleading.

Response:
The use of functional module is used frequently when discussing protein-protein interactions, protein networks and protein complexes. As INPP5E and EHBP1 do not directly interact (shown by co-IP in Figure S1F), but EHBP1 is affected when INPP5E is affected, describing their relationship as one working in a similar functional module is the most accurate term.

It is unclear if the particular INPP5E-KD (knockdown) retinal organoids used in this study were generated in prior studies. If not, the authors should validate the knockdown/knockout here.

Response:
The INPP5E^{KD} cells were generated previously by the lab of Dr. Thomas Theil at the University of Edinburgh, Edinburgh, Scotland. They were validated and characterized previously (see: https: // doi.o rg /1 0 .1 01 6/ j.celrep.20 22 .11 08 11). This is clearly referenced in lines 96.

In line 20 of the abstract, the authors should not suggest that EHBP1 functions in photoreceptor cilia, since they do not provide enough evidence for this suggestion. Instead, they could conclude that EHBP1 may have some role in photoreceptor cells (broadly) and save speculation about the CC and OS for other sections.

Response:
The text has been adjusted to tone down the claims of EHBP1 within the retina and photoreceptor cells.

Line 55 - The point about most proteins forming a complex is unnecessary and should be removed.

© 2026. Published by The Company of Biologists under the terms of the Creative Commons Attribution License (https://creativecommons.org/licenses/by/4.0/).

Response:
This point has been adjusted and rewritten, removing this point (Lines 54-55)

Line 75 states that EHBP1 was localized to the outer segment membrane of photoreceptors, which is inaccurate. Similar: Line 303 - the title of the section "EHBP1 localizes to the outer segment membrane..." is inaccurate since there are no outer segments here.

Response:
The use of outer segment has been adjusted to variations on photoreceptor outer membrane throughout the text (lines 74, 332)

In Fig. 2A-C it is difficult to see the localization of EHBP1 immunostaining due to the green and cyan overlay. The authors should add just the EHBP1 channel + DAPI.

Response:
Images in Figure 2 have been adjusted so cilia cutouts are more clear. However, color usage and larger images remain unaffected.

Can the authors clarify the point of the Fig. 2E and Fig. 2F analyses? EHBP1 fluorescence intensity relative to a standard ciliary/basal body marker is needed for the comparison.

Response:
Analysis of the EHBP1 localization has been updated to show intensity of EHBP1 relative to the basal body or ciliary marker. This is represented in the graphs and explained further in the updated quantification and statistical analysis portion of the methods.

Line 309 states that "control" retinal organoids were used to determine the localization of EHBP1 in the developing retina and photoreceptors. What are control retinal organoids, and is there literature evidence that proves that they contain human-like photoreceptors? Then, on line 311, "mature" organoids containing "maturing" photoreceptors are described. This needs clarification with literature support. Are the outermost cells in retinal organoids equivalent to developing photoreceptor neurons?

Response:
Control retinal organoids were generated from iPSCs derived from healthy control cells (referenced in line 75). In depth analysis of retinal organoid generation and confirmation of photoreceptor generation in these organoids can be seen in our recent pre-print on BioRxiv (**B I ORXI V/ 20 25 /6 72 060**). Outer segments on retinal organoids are rudimentary and do not reflect the same structure of fully mature photoreceptors found in humans or mice. The text has been adjusted for clarification (**376-379).**

Fig. S4 A, B: Why would EHBP1, a BB protein, be so diffusely localized in organoid cells? Pericentrin is not clearly shown in these images. Why weren't PCTN and ARL13B immunostaining used in "mature" organoids?

Response:
While it is unclear why EHBP1 is so diffusely localized in the organoid cell, it's possible that the multiple cell types present in retinal organoids increases background expression, however EHBP1 could, on rare occasions, be seen in the ciliary compartment of internal cilia similar to our observations in human ciliated cells (Figure 2).

ARL13B expression in mature retinal organoids is affected by the knockout of INPP5E (as described in **B I ORXI V/2 02 5/6 72 06 0**and immunofluorescent analysis of EHBP1 and ARL13B in the organoids showed no co-localization. For this reason, we did not include the images in the original publication. However, we have now added the images, and further analysis of EHBP1 expression in the outer and inner nuclear layers, in figure S5. Due to tissue and data availability, though, only ARL13B and EHBP1 are shown.

Line 325 - The authors conclude that there is a mild trafficking defect in INPP5E mutant fibroblasts based on Fig. 2C,D because slightly fewer cilia had EHBP1 along the axoneme. EHBP1

© 2026. Published by The Company of Biologists under the terms of the Creative Commons Attribution License (https://creativecommons.org/licenses/by/4.0/).

is predominantly at the BB in all conditions, so it is hard to understand the significance of this conclusion.

Response:
For clarity, the use of trafficking defects has been changed to localization defects (line 397). The slight, but significant defect in the localization of EHBP1 in patient fibroblasts highlights the likelihood that INPP5E is affecting the localization of EHBP1 in these cells.

Line 299 is a long sentence that should be rewritten for readability.

Response:
The sentence has been rewritten.

Line 392 - The authors make an excessive stretch by suggesting a potential role for EHBP1 in OS disc phagocytosis. None of their data is close to supporting this suggestion. It should be removed.

Response:
This claim has been removed.

Line 414 describes data that are not shown. The authors must explain why this data was not included.

Response:
The data was previously unpublished, but has recently been uploaded to bioRxiv (**BIORXIV/2025/672060)**.

From Reviewer 2:

Major Points

EHBP1 was identified as the most significantly enriched interactor in the INPP5E proximity proteomics screen conducted in IMCD3 Flp-In cells, yet the authors report no detectable ciliary localization of EHBP1 in IMCD3 cells by immunofluorescence. This discrepancy warrants further clarification. Could EHBP1 be interacting with a non-ciliary pool of INPP5E, or engaging transiently during trafficking events not captured by fixed-cell imaging? A brief discussion of this point would help reconcile the proteomic and localization data.

Response:
It is likely that the EHBP1 antibody is human specific on IF, as the membrane staining in the IMCD3 lines is not seen in any human-specific cell lines. Western blot analysis shows a faint band in IMCD3 cells below the expected height of EHBP1 (Figure S1E), however with more non-specific bands than the comparable human cell lines (RPE and HEK293T). While it is possible that EHBP1 was interacting with non-ciliary pools of EHBP1 in the IMCD3 cells, it is less likely as miniTurbo-INPP5E reliably expresses at the cilium and biotinylates proteins in the cilium with high specificity (Figure S1A). We have included discussion points regarding this in the discussion to prevent confusion (Lines 407-411).

Another concern is that the authors do not demonstrate that INPP5E is actually knocked out in the retinal organoids described throughout Figure 4 and Figure S4. No protein or transcript-level validation of INPP5E loss is provided. Without this, the interpretation of EHBP1 mis localization or changes in expression cannot be confidently attributed to INPP5E deficiency. This is a critical omission, as the central premise of the retinal organoid analysis rests on the comparison between control and INPP5EKD.

Response:
The knockout of INPP5E has been verified previously by the lab that generated the cell line (see: https: // do i.o rg /1 0 .1 01 6/ j.celrep.20 22 .11 08 11 **), which has been referenced in the text (line 96)**. Additionally, the loss of INPP5E in retinal organoids is confirmed in our recently published pre-print (**B I ORXI V/ 20 25 /6 72 060**) which describes the phenotype of the $INPP5E^{KD}$ organoids

© 2026. Published by The Company of Biologists under the terms of the Creative Commons Attribution License (https://creativecommons.org/licenses/by/4.0/).

in great detail.

A second major issue arises from the appearance of photoreceptor outer segments in INPP5EKD organoids at d230. The structures interpreted as outer segments in Figure 4 appear more prominent or expanded in knockdown than in controls. This contradicts existing in vivo data from INPP5E knockout mouse models, which consistently show very rapid outer segment disorganization or degeneration. Before concluding that EHBP1 fails to localize to photoreceptor outer segments, the authors must first confirm that these structures are indeed bona fide OS. This could be achieved by immunostaining for other canonical OS markers. Without this, the observed absence of EHBP1 in the OS could reflect a loss or alteration of outer segment identity, rather than a trafficking defect.

Response:
This is an understandable concern. We have quantified the morphology and phenotype of the outer segments in the INPP5E^{KD} organoids extensively in a recently uploaded pre-print (**B I ORXI V/ 20 25 /6 72 060**). This includes staining for the canonical outer segment markers rhodopsin and opsin R/G. Additionally, we discuss the possible reasonings for why the OS in our organoids are longer, contrary to what was seen in mouse models.

Response:
In several organoid images (e.g., Figure S4), EHBP1 appears reduced in the OS layer of INPP5EKD organoids but is increased in deeper retinal layers. However, no effort is made to identify which retinal cell types are expressing EHBP1 in these locations. Without co-staining for cell-type markers such as CRX (photoreceptors), CHX10 (retinal progenitors), or BRN3A (ganglion cells), the interpretation that EHBP1 is mis localized within photoreceptors remains speculative. The increase in EHBP1 signal in the inner retina may reflect a shift in expression to another cell type or a structural reorganization within the tissue.

Response:
Analysis of EHBP1 expression in the deeper organoid sublayers (ONL and INL) has been completed and show no significant differences in EHBP1 expression between control and INPP5E^{KD} organoids.
We have included these data as Supplemental figures 4 and 5.

As the focus of the retinal organoid section of the manuscript is on INPP5E - which localizes in the cilium of d35 organoids and connecting cilium of d230 organoids - we focused our analysis on cilia and photoreceptors. Future work could study cell-type specific expression of EHBP1.

Lastly, there appears to be a qualitative reduction in ARL13B staining in INPP5EKD organoids, raising the possibility that cilia formation or stability is impaired more broadly (Fig S4). This should be quantified by measuring ARL13B-positive cilia frequency, length, and intensity across photoreceptor cell layers. Without this information, it remains unclear whether EHBP1 loss from the cilium is a specific trafficking defect or part of a more general ciliary disruption.

Response:
This is a valid point, and the structure and frequency of cilia in control versus INPP5E^{KD} organoids has been quantified and expanded upon extensively in our recently published pre-print on BioRxiv (**B I ORXI V/ 20 25 /6 72 060**)

Minor Points

The manuscript uses the term "trafficking defect" to describe EHBP1 behavior in retinal organoids. This interpretation should be stated more cautiously, particularly since the structural presence of outer segments and cilia has not yet been confirmed in INPP5EKD conditions.

Response:
The use of “trafficking defect” has been adjusted to “localization defect” throughout the text to avoid false speculation. However, the structural presence of outer segments has been confirmed in

© 2026. Published by The Company of Biologists under the terms of the Creative Commons Attribution License (https://creativecommons.org/licenses/by/4.0/).

the INPP5E^{KD} condition (**BIORXIV/2025/672060**), so the hypothesis that INPP5E is regulating trafficking fits the hypothesis proposed both in that paper and in the Gupta et al. (2025) paper (referenced).

In Figure 3, the cell line used for Cytochalasin D treatment is not clearly stated in the figure legend and should be explicitly identified as human RPE. For consistency and clarity, the biological system used in each figure should be clearly labeled.

Response:
Cell line information has been added to the relevant figures and figure legends for clarification.

Several figures, especially those involving retinal organoids, would benefit from improved side-by-side comparisons. In some cases, differences in image brightness or focal depth complicate the interpretation of morphological differences between control and knockout samples.

Response:
The retinal organoid figures have been adjusted as proposed to improve side-by-side comparisons.

The authors report changes in EHBP1 signal across different retinal layers, but these observations are not quantified. Including mean fluorescence intensity measurements or area-based quantification for both outer and inner layers would add rigor to the interpretation.

Response:
Quantification of EHBP1 expression in the outer and inner nuclear layer of retinal organoids at both d35 and d230 show no significant changes between control and knock-out organoids, and that the only significant change in EHBP1 localization occurs in the outer segment membrane of d230 organoids. These data have been added to the manuscript (Fig S4 E-F; Fig. S5 C-D) and the text has been adjusted accordingly.

Additional Notes:

A co-immunoprecipitation of INPP5E and EHBP1 was performed and results added to Figure S1F.

Second decision letter

MS ID#: jcs.264091R1

MS TITLE: The EH Binding protein EHBP1 operates in a ciliary functional module affected by INPP5E dysfunction

AUTHORS: Kae R Whiting; Mariam G Aslanyan; Imke Peters; Stef J F Letterboer; Mohamed-Ali Jarboui; Tina Beyer; Katrin Dahlke; Karsten Boldt; Ronald Roepman

ARTICLE TYPE: Research Article

Dear Dr Roepman,

We have now reached a decision on the above manuscript.

To see the reviewers' reports and a copy of this decision letter, please go to:

As you will see, the reviewers continue raise a number of substantial criticisms that prevent me from accepting the revised paper. It was felt that the data remains too preliminary and western data to be weak validation for the IF staining inconsistencies proposed to be species-based, especially with a reagent which has been published by others to work in mouse (i.e. doi: 10.1038/s41467-022-34958-3). Comparison to another commercial antibody with

© 2026. Published by The Company of Biologists under the terms of the Creative Commons Attribution License (https://creativecommons.org/licenses/by/4.0/).

immunofluorescence in IMCD3 cells was proposed by both reviewers, alternately an endogenous tag or overexpression; this would be a minimum. We acknowledge this had been attempted in the new data provided in the point-by-point responses, but here the GFP and FLAG images are the same but slightly shifted in field-of-view. Localization is important to resolve, as much of the literature has EHBP1 at various endomembrane compartments and involved in vesicle architecture/curvature independent of cilia, but as we know there is a lot of overlap and dynamic exchange and seems more fitting to the broader localization you see in retinal organoids. Finally, toning down claims of function is important for clarity on the paper's message, as no functional work on EHBP1 has been included on how it impacts ciliary behaviour and the title was found to be misleading. A revised version might prove acceptable, if you can address their concerns. If you think that you can deal satisfactorily with the criticisms on revision, I would be pleased to see a revised manuscript. We would then return it to the reviewers.

Reviewer 1

Advance summary and potential significance to field

The revised manuscript by Whiting et al. titled "The EH Binding protein EHBP1 operates in a ciliary functional module affected by INPP5E dysfunction," is an improved manuscript due to the more reasonably described conclusions.

Uncertainty remains about EHBP1 localization in IMCD3 cells, which were the cells used in the key miniTurbo-INPP5e screen. New westerns suggest "less specific" binding of the anti-EHBP1 antibody in mouse cells compared to human cells as a possible reason why EHBP1 is not localized in IMCD3 cell primary cilia. As additional validation, transfected IMCD3 microscopy data were presented to the reviewer that were deemed not informative for inclusion in the manuscript. Here, IMCD3 cells were transfected for expression of either GFP-hEHBP1, 3xFlag-EHBP1, or 3xHA-EHBP1 fusions. The microscopy images representing the GFP-hEHBP1 and 3xFlag-EHBP1 conditions are the exact same; the FOVs are just slightly shifted. The authors are asked to provide the correct images for a proper evaluation. Nevertheless, can the authors clarify how the cytoplasmic expression of all of these fusions represents non-specific expression? If they had specific expression, would the localization pattern be ciliary, or would it look like anti-EHBP1 staining in Supplemental Figure 1B? In the results, the authors state that EHBP1 is expressed at the cell membrane but also indicate that IMCD3 cells have non-specific binding (of the EHBP1 antibody presumably) (lines 299-303). The general vagueness around this antibody issue should be directly addressed, since major retinal organoid conclusions are based on immunolocalization data using this same reagent.

Reviewer 2

Advance summary and potential significance to field

Comments for the author

Major comments [Please request additional experiments only if they are essential for supporting the conclusions; authors should be encouraged to highlight any claims that are preliminary or speculative, or to discuss any pitfalls or alternative interpretations in a 'Limitations' section]
The title continues to overstate the findings of the paper. No functional data is presented so the word "operates" is not justified. EHBP1 being affected by INPP5E dysfunction is not convincing as the differences in EHBP1 in control v. patient fibroblasts are minimal (limited to a small difference in panel 2D) and the organoids are not characterized well enough to understand what the difference in staining means. The associated paper in bioRXve does not resolve the uncertainty as the staining with Rho and RG Opsin antibodies in that work do not look anything like the PNA staining shown in this paper.

The lack of staining in IMCD3 cells with the antibody is plausible, but I am surprised that the authors did not attempt to identify an alternative antibody. The staining in the organoids is not of high enough quality to understand what is being labeled and as such, the interpretation of the

© 2026. Published by The Company of Biologists under the terms of the Creative Commons Attribution License (https://creativecommons.org/licenses/by/4.0/).

results is completely speculative. Much of the uncertainty of the work could have been resolved with an antibody against mouse protein used to stain control and INPP5E mutant retinas.

Minor comments

Second revision

Author response to reviewers' comments

Rebuttal for reviewers:

From Editor:

It was felt that the data remains too preliminary and western data to be weak validation for the IF staining inconsistencies proposed to be species-based, especially with a reagent which has been published by others to work in mouse (i.e. doi: 10.1038/s41467-022-34958-3). Comparison to another commercial antibody with immunofluorescence in IMCD3 cells was proposed by both reviewers, alternately an endogenous tag or overexpression; this would be a minimum.

Response:

To further specify this request, and in light of a lack of valiated antibodies against EHBP1, discussing this issue with the editor resulted in the consensus that a downregulation of EHBP1 in human cells would also be a suitable test for specificity of the EHBP1 antibodies used in this manuscript.

To that end, we have used siRNA knock-down of EHBP1 in hTERT-RPE1 cells (siPOOLS from siTOOLS Biotech), which showed a strong reduction in EHBP1 expression on western blot analysis, as well as a significant decrease in EHBP1 expression at both the basal body and ciliary compartment on immunofluorescence (Results section, lines 314-317). We believe this confirms the specificity of the EHBP1 antibody on IF in human cells (See figure below and Supplemental figure 2).

© 2026. Published by The Company of Biologists under the terms of the Creative Commons Attribution License (https://creativecommons.org/licenses/by/4.0/).

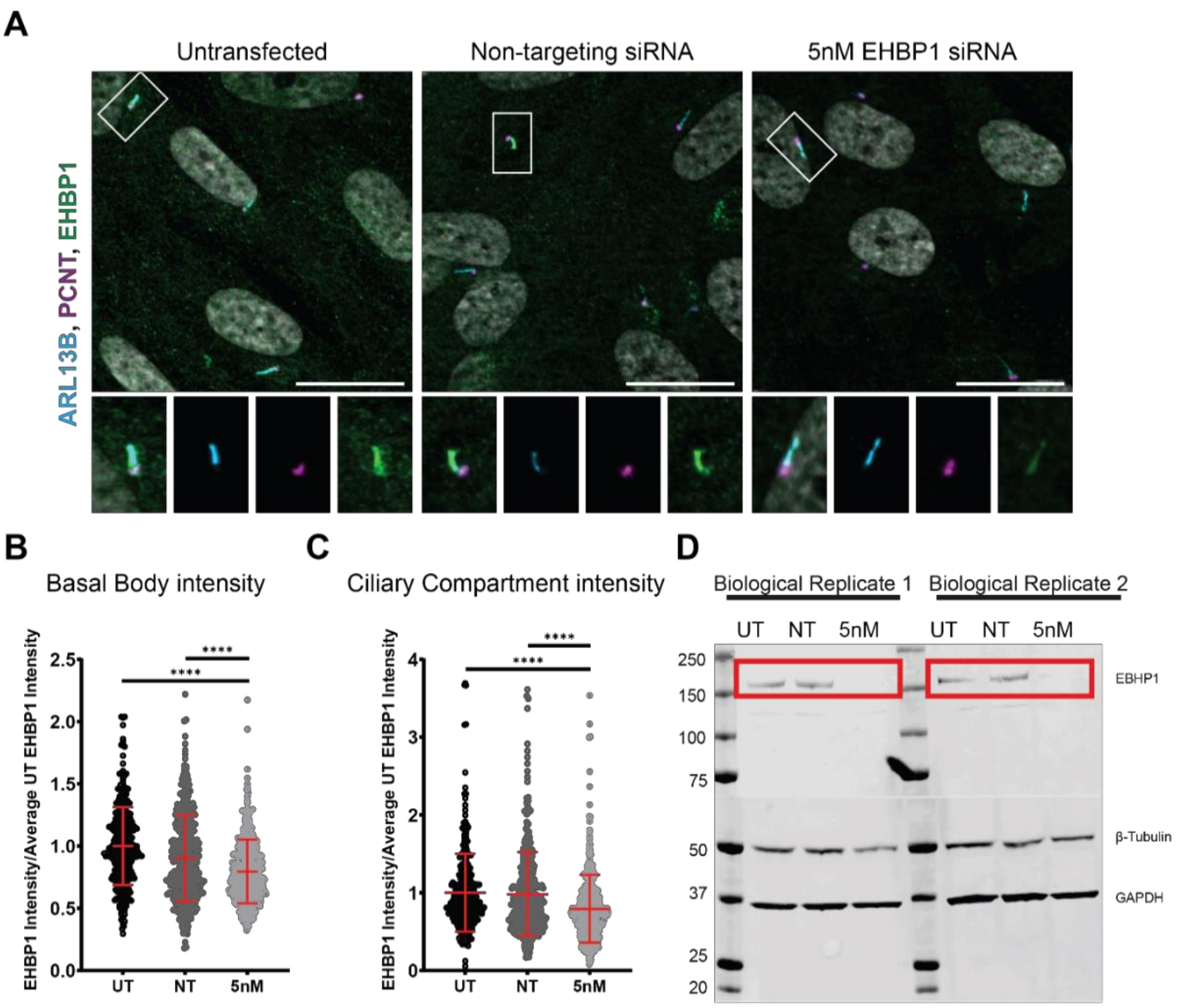

[Comment from reviewers and editor]

We acknowledge this had been attempted in the new data provided in the point-by-point responses, but here the GFP and FLAG images are the same but slightly shifted in field-of-view.

Response: The image reuse was a copy/paste oversight on our part for which we deeply apologize. We have provided the correct version of the intended figure and are happy to provide additional example images per the editor's request.

© 2026. Published by The Company of Biologists under the terms of the Creative Commons Attribution License (https://creativecommons.org/licenses/by/4.0/).

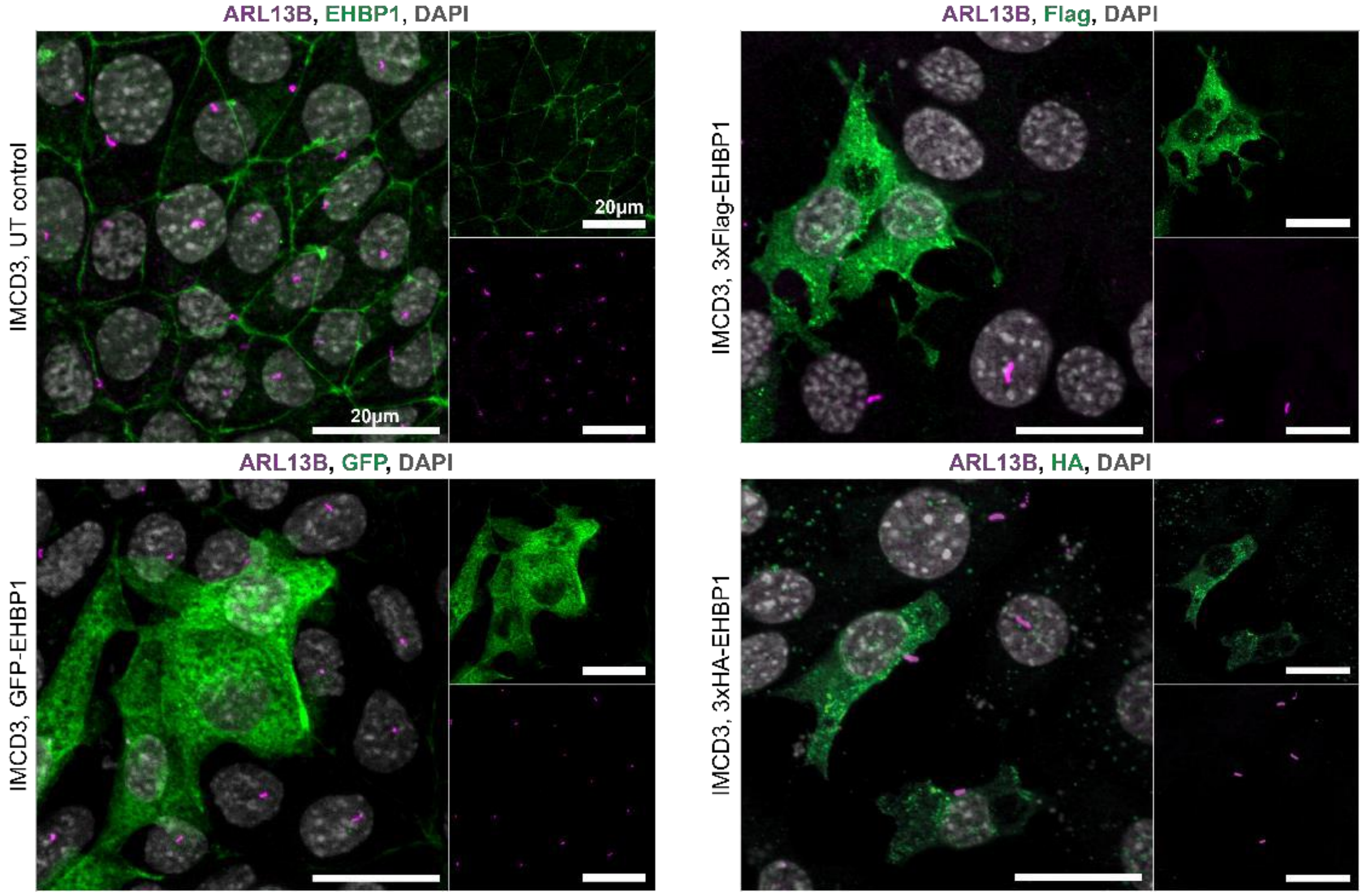

[Comment from reviewers and editor]

Localization is important to resolve, as much of the literature has EHBP1 at various endomembrane compartments and involved in vesicle architecture/curvature independent of cilia, but as we know there is a lot of overlap and dynamic exchange and seems more fitting to the broader localization you see in retinal organoids.

Response: We agree that localization is important in the context of the article. Therefore we have attempted to resolve the localization of EHBP1 in a variety of in-vitro models (hTERT-RPE1, human fibroblasts, human retinal organoids and mouse IMCD3 cells) using both an antibody and recombinant fusion proteins. Variation in the performance of antibodies across different species is a known limitation, and since our work was mostly focused on the relevance of the EHBP1 protein in human cells, we did not focus on studying the protein localization in mouse cells. The recombinant fusion proteins that were used were also human, which also limits our interpretation of their localization in the mouse IMCD3 cell model.

Finally, toning down claims of function is important for clarity on the paper's message, as no functional work on EHBP1 has been included on how it impacts ciliary behavior and the title was found to be misleading

Response:

Our title, **"The EH Binding protein EHBP1 operates in a ciliary functional module affected by INPP5E dysfunction"** to our opinion does coin our main findings, with the following rationale:

EHBP1 was identified prominently in the proximity proteome of INPP5E, suggesting it is part of a shared functional module (reflected by our proximiy proteomics results), but our in vitro association studies did not confirm it as a direct binding partner. Therefore, with "functional module", a term commonly used to pinpoint results from affinity- and proximity proteomics, we don't overclaim a direct mechanistic association (for example as in a protein complex), which indeed we don't show. The fact that INPP5E loss affects EHPB1 behaviour/localisation in the cilia

© 2026. Published by The Company of Biologists under the terms of the Creative Commons Attribution License (https://creativecommons.org/licenses/by/4.0/).

of cells and retinal organoids confirms the relationship between EHBP1 and INPP5E, without pinpointing an exact pathway. “Functional module” in this respect is the best term to coin these findings.

Nevertheless, if the editor does not agree with this rationale, we are open to discuss how best to adjust our phrasing.

Reviewer 1:

The microscopy images representing the GFP-hEHBP1 and 3xFlag-EHBP1 conditions are the exact same; the FOVs are just slightly shifted. The authors are asked to provide the correct images for a proper evaluation.

Response: See above. This was a mistake on our part, and corrected images are provided.

Nevertheless, can the authors clarify how the cytoplasmic expression of all of these fusions represents non-specific expression? If they had specific expression, would the localization pattern be ciliary, or would it look like anti-EHBP1 staining in Supplemental Figure 1B? In the results, the authors state that EHBP1 is expressed at the cell membrane but also indicate that IMCD3 cells have non-specific binding (of the EHBP1 antibody presumably) (lines 299-303). The general vagueness around this antibody issue should be directly addressed, since major retinal organoid conclusions are based on immunolocalization data using this same reagent.

Response: Referring to the non-specific expression pertaining to the localization of the recombinant EHBP1 proteins: transient overexpression is known to result in artefacts and non-specific expression of proteins which deviates from their endogenous localization. This is a particularly common problem with proteins which localize to the cilium. We have addressed the issues surrounding the antibody extensively above. Furthermore, we do not doubt the validity of the antibody we have used in our human based models due to high consistency of results across different models and experiments. Additionally, we do indeed see EHBP1 localizing in the cilium of developing retinal organoids, however due to the lack of tissue availability and the low prevalence of cilia in the outer and inner nuclear layers of the retinal organoids, where ciliary EHBP1 expression was seen, we kept this as a supplemental figure (Supplemental Figure 4A (white arrows)).

Reviewer 2

The title continues to overstate the findings of the paper. No functional data is presented so the word "operates" is not justified. EHBP1 being affected by INPP5E dysfunction is not convincing as the differences in EHBP1 in control v. patient fibroblasts are minimal (limited to a small difference in panel 2D) and the organoids are not characterized well enough to understand what the difference in staining means.

Response: We have discussed our rationale of using the term “functional module” in the title above. It can be argued that when a protein is part of a functional module, it operates in that module: we don’t consider this overstating its functionality, as indeed loss of INPP5E results in loss of ciliary localisation of EHBP1. We also argue that characterisation of the organoids was performed at current standards, which unfortunately provide less details than analysis of mouse retinae, but warrant our conclusions. Future research will be aimed at understanding the observed differences and mechanistically dissecting the function of EHBP1 in the cilia of different tissues and organs during development. Teaming up with the scientists that published the mouse model would then be a logical step.

The associated paper in bioRXve does not resolve the uncertainty as the staining with Rho and RG Opsin antibodies in that work do not look anything like the PNA staining shown in this paper.

Response: PNA stains all outer segment membranes (including both rod and cone photoreceptors), while rhodopsin and opsin stain for proteins within the outer segments of either rod and cone photoreceptors (respectively). It is therefore logical that the stainings will not be identical. However PNA is often used as a marker for outer segment membranes in previously published

© 2026. Published by The Company of Biologists under the terms of the Creative Commons Attribution License (https://creativecommons.org/licenses/by/4.0/).

papers using retinal organoids (Achberger, K., 2019, DOI: 10.7554/eLife.46188; Berber, P., 2021, DOI: 10.20517/jtgg.2021.35; Buille-dit-Bille, E. 2025, DOI: 10.1038/s41598-024-83164-2). We have provided images of both control and INPP5E^{KD} organoids triple stained with Rho, Opsin, and PNA, which show that opsin and PNA markers co-localize but only to a certain degree.

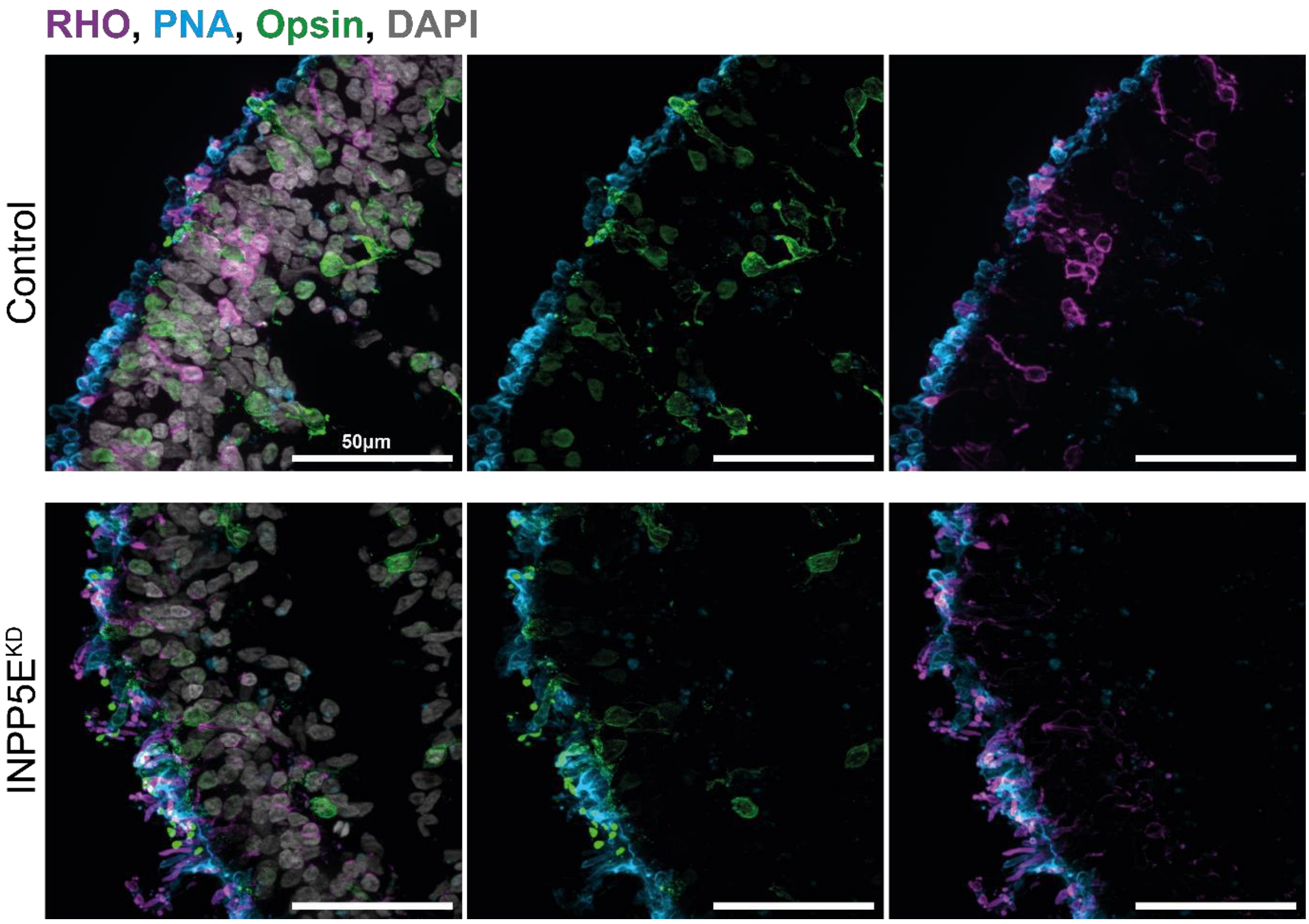

The lack of staining in IMCD3 cells with the antibody is plausible, but I am surprised that the authors did not attempt to identify an alternative antibody

Response: Since our interest focuses on the localization and function of EHBP1 in human cells, where the currently used EHBP1 antibody worked reliably, we chose not to focus on IMCD3 cells which were not of primary interest for this work. To address this concern of both reviewers and validate the specificity of the anti-EHBP1 antibodies used in this manuscript, we have now included siRNA knock-down of EHBP1 in hTERT-RPE1 cells, shown in supplemental figure 2 and indicated in more detail above.

The staining in the organoids is not of high enough quality to understand what is being labeled and as such, the interpretation of the results is completely speculative.

Response: It would be helpful to know which staining the reviewer is referring to. If they are referring to the EHBP1 staining at d35, this is image was intentionally kept as a supplemental. It is a potentially interesting observation that EHBP1 localized to the cilium at that stage in retinal organoid differentiation. However, given the low frequency of cilia at d35 we refrained from drawing conclusions from this and have not overstated it in the main text of the manuscript.

Much of the uncertainty of the work could have been resolved with an antibody against mouse protein used to stain control and INPP5E mutant retinas.

Response: We assume the reviewer is referring to INPP5E mutant retinas derived from mice, however this is not clearly stated. Assuming that is the case, we do not have easy access to wild type and INPP5E mouse mutant retinas and consider this outside the scope of this work.

© 2026. Published by The Company of Biologists under the terms of the Creative Commons Attribution License (https://creativecommons.org/licenses/by/4.0/).

Furthermore, our paper focuses on furthering our knowledge of EHBP1 at the cilium in human-derived model systems. While further work will help elucidate the precise function of EHBP1 in relation to INPP5E at the cilium, we believe our manuscript provides conclusive evidence that EHBP1 localizes to the ciliary compartment in human cells and photoreceptors in retinal organoids.

Third decision letter

MS ID#: jcs.264091R2

MS Title: The EH Binding protein EHBP1 operates in a ciliary functional module affected by INPP5E dysfunction

Authors: Kae R Whiting; Mariam G Aslanyan; Imke Peters; Stef J F Letterboer; Mohamed-Ali Jarboui; Tina Beyer; Katrin Dahlke; Karsten Boldt; Ronald Roepman

Article Type: Research Article

Dear Ronald and Kae,

I am thrilled to tell you that your manuscript has been accepted for publication in Journal of Cell Science, with a few editorial changes and pending standard publication integrity checks.

© 2026. Published by The Company of Biologists under the terms of the Creative Commons Attribution License (https://creativecommons.org/licenses/by/4.0/).
